# An octa­nuclear nickel(II) pyrazolate cluster with a cubic Ni_8_ core and its methyl- and *n*-octyl-functionalized derivatives

**DOI:** 10.1107/S2056989023010101

**Published:** 2023-11-30

**Authors:** Wisam A. Al Isawi, Matthias Zeller, Gellert Mezei

**Affiliations:** a Western Michigan University, Department of Chemistry, 1903 W. Michigan Ave., Kalamazoo, MI 49008, USA; b Department of Chemistry, Purdue University, 560 Oval Dr., West Lafayette, IN 47907-2084, USA; Università di Parma, Italy

**Keywords:** crystal structure, nickel, pyrazolate complex, octa­nuclear core

## Abstract

The mol­ecular and crystal structure of a discrete [Ni_8_(μ_4_-OH)_6_(μ-4-*R*pz)_12_]^2−^ (*R* = H; pz = pyrazolate anion, C_3_H_3_N_2_^−^) cluster with an unprecedented, perfectly cubic arrangement of its eight Ni centers is reported, along with its lower-symmetry alkyl-functionalized (*R* = methyl and *n*-oct­yl) derivatives. Crystals of the latter two were obtained with two identical counter-ions (Bu_4_N^+^), whereas the crystal of the complex with the parent pyrazole ligand has one Me_4_N^+^ and one Bu_4_N^+^ counter-ion. The methyl derivative incorporates 1,2-di­chloro­ethane solvent mol­ecules in its crystal structure, whereas the other two are solvent-free. The compounds are tetra­butyl­aza­nium tetra­methyl­aza­nium hexa-μ_4_-hydroxido-dodeca-μ_2_-pyrazolato-*hexa­hedro*-octa­nickel, (C_16_H_36_N)(C_4_H_12_N)[Ni_8_(C_3_H_3_N_2_)_12_(OH)_6_] or (Bu_4_N)(Me_4_N)[Ni_8_(μ_4_-OH)_6_(μ-pz)_12_] (**1**), bis­(tetra­butyl­aza­nium) hexa-μ_4_-hydroxido-dodeca-μ_2_-(4-methyl­pyrazolato)-*hexa­hedro*-octa­nickel 1,2-di­chloro­ethane 7.196-solvate, (C_16_H_36_N)_2_[Ni_8_(C_4_H_5_N_2_)_12_(OH)_6_]·7.196C_2_H_4_Cl_2_ or (Bu_4_N)_2_[Ni_8_(μ_4_-OH)_6_(μ-4-Mepz)_12_]·7.196(ClCH_2_CH_2_Cl) (**2**), and bis­(tetra­butyl­aza­nium) hexa-μ_4_-hydroxido-dodeca-μ_2_-(4-octylpyrazolato)-*hexa­hedro*-octa­nickel, (C_16_H_36_N)_2_[Ni_8_(C_11_H_19_N_2_)_12_(OH)_6_] or (Bu_4_N)_2_[Ni_8_(μ_4_-OH)_6_(μ-4-^*n*^Octpz)_12_] (**3**). All counter-ions are disordered (with the exception of one Bu_4_N^+^ in **3**). Some of the octyl chains of **3** (the crystal is twinned by non-merohedry) are also disordered. Various structural features are discussed and contrasted with those of other known [Ni_8_(μ_4_-OH)_6_(μ-4-*R*pz)_12_]^2−^ complexes, including extended three-dimensional metal–organic frameworks. In all three structures, the Ni_8_ units are lined up in columns.

## Abstract 2

The mol­ecular and crystal structure of a discrete [Ni_8_(μ_4_-OH)_6_(μ-4-*R*pz)_12_]^2−^ (*R* = H; pz = pyrazolato anion) cluster with an unprecedented, perfectly cubic arrangement of its eight Ni centers is reported, along with its lower-symmetry alkyl-functionalized (*R* = methyl and *n*-oct­yl) derivatives.

## 1. Chemical context

Polynuclear metal–organic complexes display inter­esting properties that allow for numerous applications in fields such as mol­ecular magnetism (Milios & Winpenny, 2015 ▸; Papatriantafyllopoulou *et al.*, 2016 ▸; Zheng *et al.*, 2018 ▸; Li *et al.*, 2022 ▸), luminescence (Yam & Lo, 1999 ▸; Balzani *et al.*, 1996 ▸) and catalysis in organic synthesis (Nath *et al.*, 2020 ▸; Singh *et al.*, 2020 ▸; Wu *et al.*, 2023 ▸). Discrete polynuclear clusters with their metal ions arranged in various different geometries have been reported, including grids (Ruben *et al.*, 2004 ▸), macrocycles (Yang, 2018 ▸; Zaleski, 2022 ▸), and Platonic and Archimedean solids (Luo *et al.*, 2023 ▸). Pyrazole (pzH) is a versatile ligand for the synthesis of polynuclear metal complexes (Halcrow, 2009 ▸; Viciano-Chumillas *et al.*, 2010 ▸; Klingele *et al.*, 2009 ▸). An intriguing class of large polynuclear complexes based on pyrazole, termed nanojars, incorporate up to 36 Cu^II^ ions and have the formula [anion⊂{*cis*-Cu^II^(μ-OH)(μ-pz)}_*n*_]^*m*−^ (anion = CO_3_^2−^, SO_4_^2−^, Cl^−^, *etc*.; *n* = 26–36; *m* = 1 or 2; Mezei *et al.*, 2004 ▸; Ahmed & Mezei, 2016 ▸; Al Isawi *et al.*, 2023 ▸). Although many coord­ination complexes can be obtained with various different metal ions, nanojars could only be obtained so far with Cu^II^ ions. Under identical reaction conditions, Ni^II^ (a metal ion with similar coordination geometries), produces [Ni_8_(μ_4_-OH)_6_(μ-pz)_12_]^2−^ instead of nanojars (Al Isawi *et al.*, 2018 ▸). We originally reported a low-symmetry (monoclinic, *P*2_1_/*c*) solvated crystal structure for this complex. Herein, we report a high-symmetry (tetra­gonal, *I*4/*mmm*), solvent-free structure, (Me_4_N)(Bu_4_N)[Ni_8_(μ_4_-OH)_6_(μ-pz)_12_] (**1**). To expand solu­bility into non-polar solvents, its alkyl (methyl and *n*-oct­yl) derivatives have also been prepared, and the crystal structures of (Bu_4_N)_2_[Ni_8_(μ_4_-OH)_6_(μ-4-Mepz)_12_]·7.196(ClCH_2_CH_2_Cl) (**2**) and (Bu_4_N)_2_[Ni_8_(μ_4_-OH)_6_(μ-4-^*n*^Octpz)_12_] (**3**) are presented here. 
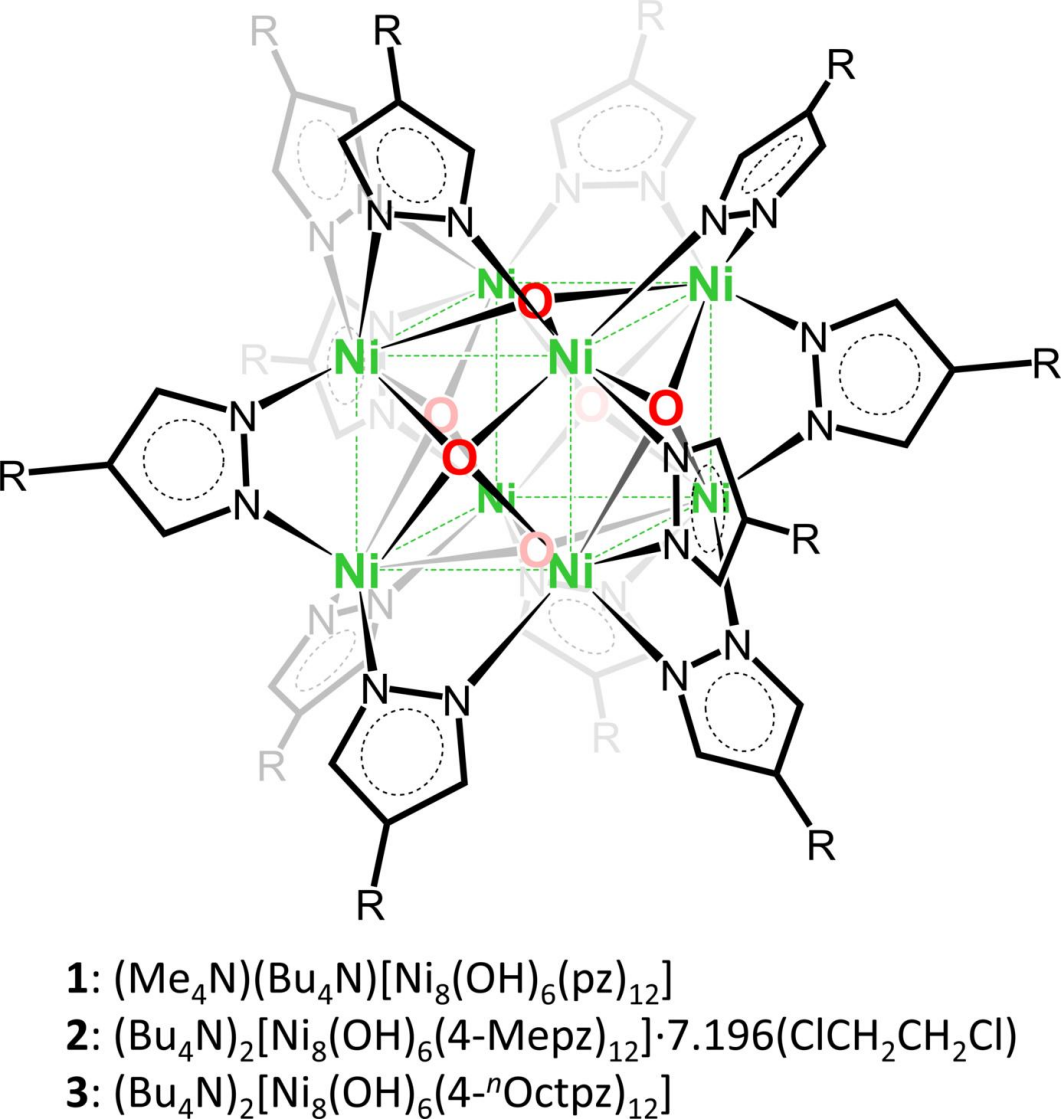


## 2. Structural commentary

**Figure 1:**
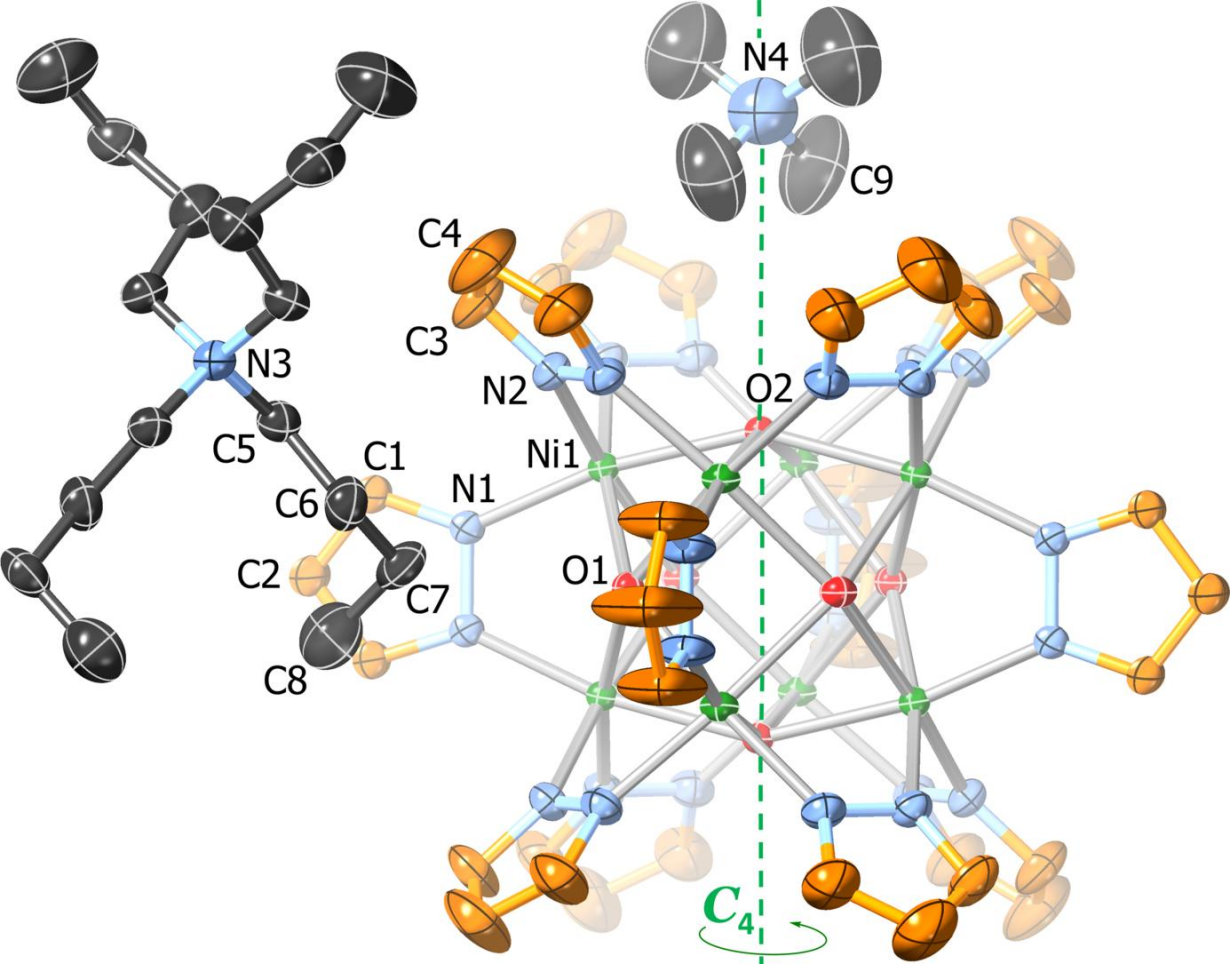
Displacement ellipsoid plot (50% probability) of the crystal structure of [Ni_8_(μ_4_-OH)_6_(μ-pz)_12_]^2−^ (**1**). Only atoms within the asymmetric unit are labeled; counter-ion disorder and H atoms are omitted for clarity.

The anionic cluster of **1** is located on an inversion center, around a *C*_4_ rotation axis at the inter­section of three orthog­onal mirror planes (Fig. 1 ▸). One of its two counter-cations (Me_4_N^+^) is also positioned around the *C*_4_ axis, whereas the other (Bu_4_N^+^) is located at the inter­section of two mirror planes and two *C*_2_ axes. Because their mol­ecular structure is not compatible with these symmetry elements, the counter-cations are extensively disordered (see the *Refinement* section below). The eight Ni atoms have distorted octa­hedral coordination environments [*fac*-NiO_3_N_3_; O—Ni—N angles: 169.54 (15) and 169.89 (11)°] and define a regular cube, with Ni⋯Ni distances of 2.9826 (8) Å (Table 1 ▸). Despite the perfectly cubic symmetry of the Ni_8_ core, the overall crystal symmetry lacks *C*_3_ axes and additional *C*_4_ axes. Consequently, the four pyrazolate rings collinear with the *C*_4_ axis are not identical to the other eight pyrazolate rings that form 45° angles with the *C*_4_ axis, and have Ni—N bond lengths of 2.013 (3) and 2.027 (2) Å, respectively. Similarly, there are two different sets of μ_4_-OH groups that are centered above the Ni_4_ faces of the cube, at distances of 0.515 (3) and 0.513 (3) Å from the Ni_4_ mean planes, and with Ni—O bond lengths of 2.1710 (12) and 2.1742 (9) Å.

**Figure 2:**
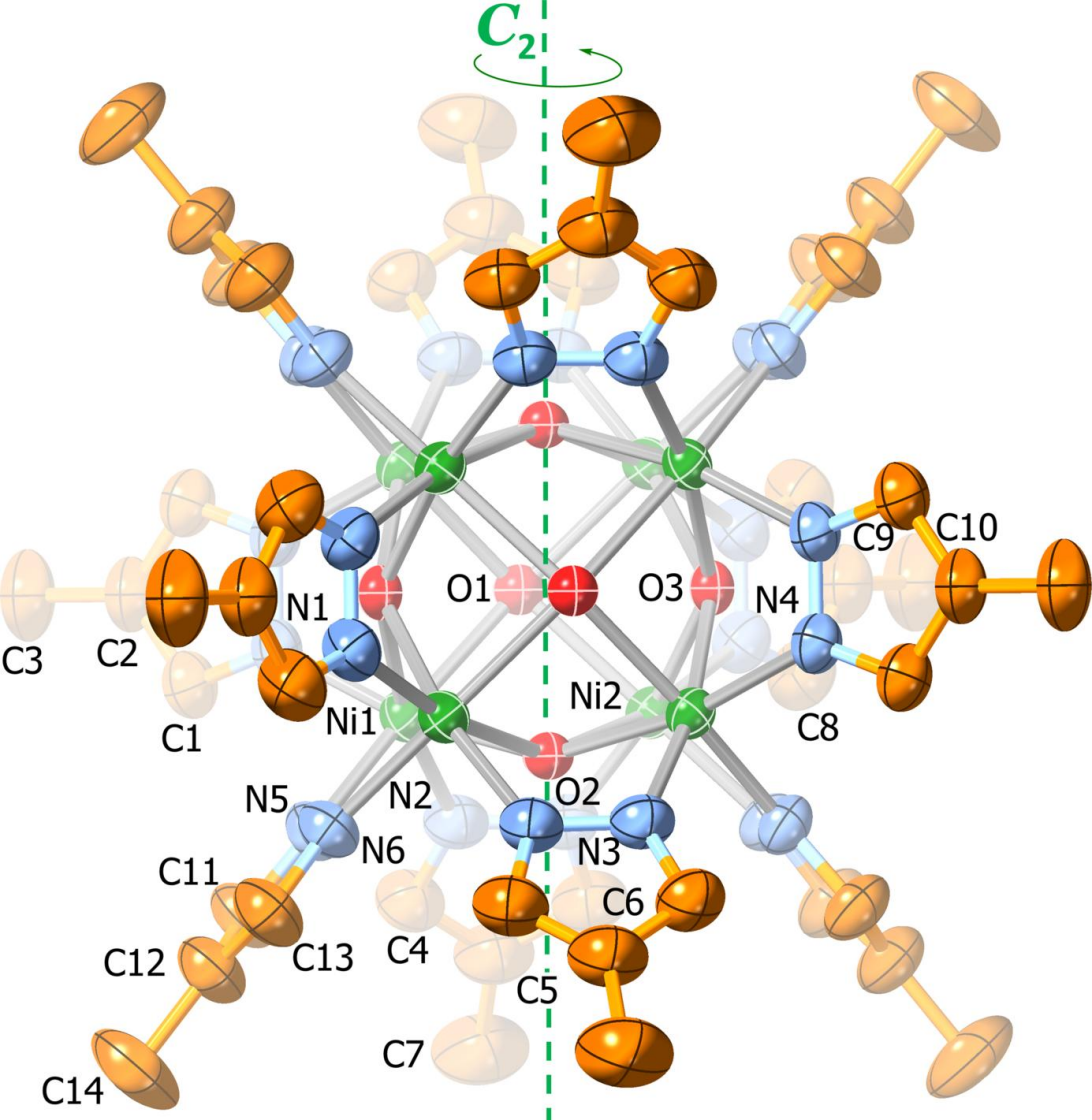
Displacement ellipsoid plot (50% probability) of the crystal structure of [Ni_8_(μ_4_-OH)_6_(μ-4-Mepz)_12_]^2−^ (**2**). Only atoms within the asymmetric unit are labeled.

**Figure 3:**
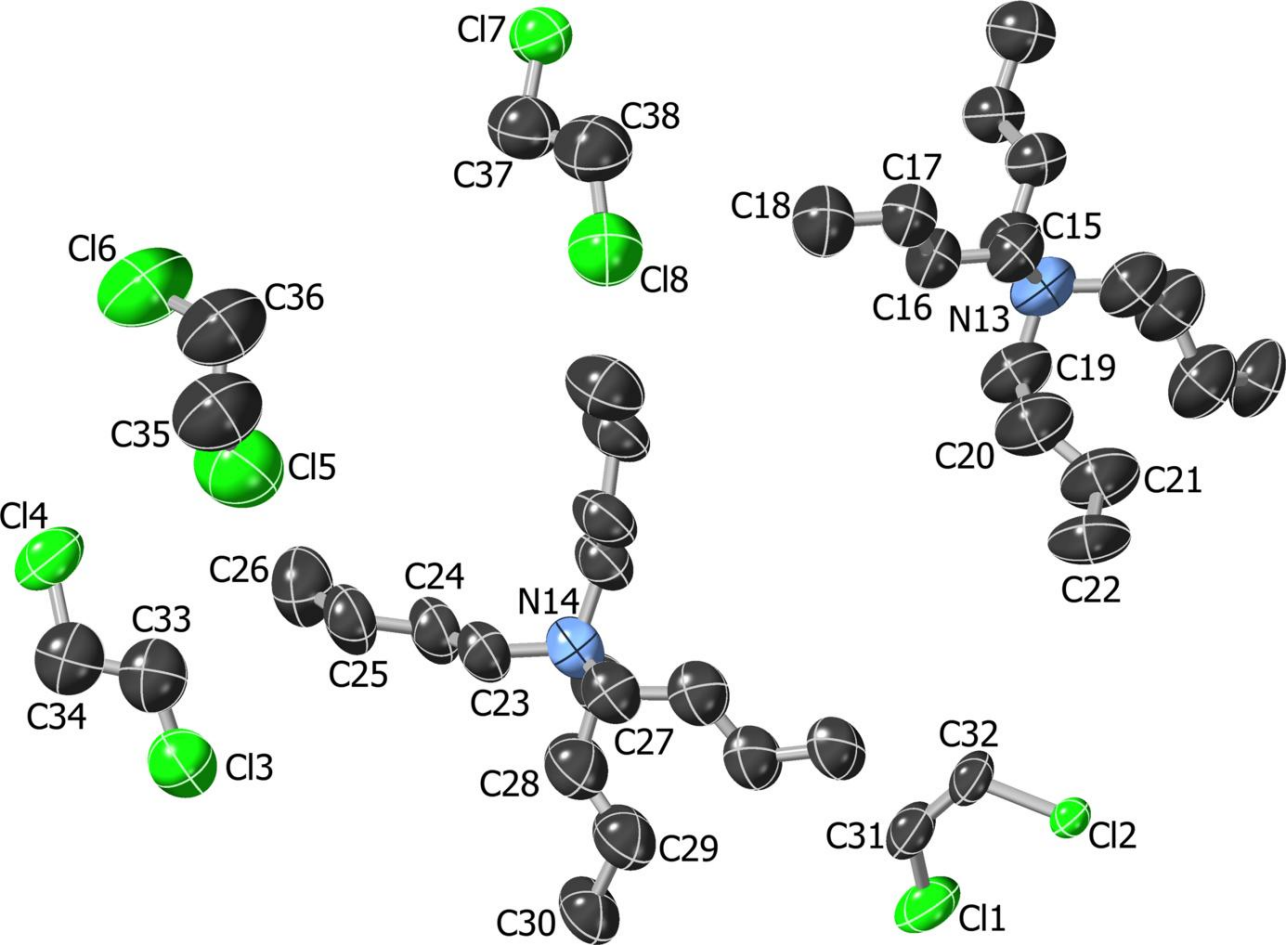
Displacement ellipsoid plot (50% probability) of the Bu_4_N^+^ counter-ions and 1,2-di­chloro­ethane solvent mol­ecules in **2**. Only atoms within the asymmetric unit are labeled; disorder and H atoms are omitted for clarity.

In **2** (ortho­rhom­bic, *Pmna*), the anionic cluster is located on an inversion center around a *C*_2_ axis and a *σ*_h_ mirror plane (Fig. 2 ▸). The two Bu_4_N^+^ cations exhibit twofold rotational symmetry and are disordered across a mirror plane (Fig. 3 ▸). Although not perfectly cubic, the Ni_8_ core displays similar Ni⋯Ni distances of 2.984 (1)–2.999 (1) Å [average: 2.993 (1) Å, slightly larger than with the parent pyrazole] and Ni—Ni—Ni angles very close to 90° [ranging from 89.92 (3) to 90.08 (3)°] (Table 1 ▸). The fold and twist angles between pyrazolate rings on opposite sides of the [Ni(4-Mepz)]_4_ faces range from 78.3 (4) to 90.3 (3)° and from 0.0 (6) to 5.9 (6)°, respectively, instead of having ideal values of 90° and 0° as observed in the case of **1** with a perfectly cubic Ni_8_ core. Other structural features, such as the Ni—O and Ni—N bond lengths, O—Ni—N angles and distances between μ_4_-OH groups and Ni_4_ faces are similar to the ones observed for the parent pyrazolate (Table 1 ▸). The structure also contains four disordered 1,2-di­chloro­ethane solvent mol­ecules (two with partial occupancy).

**Figure 4:**
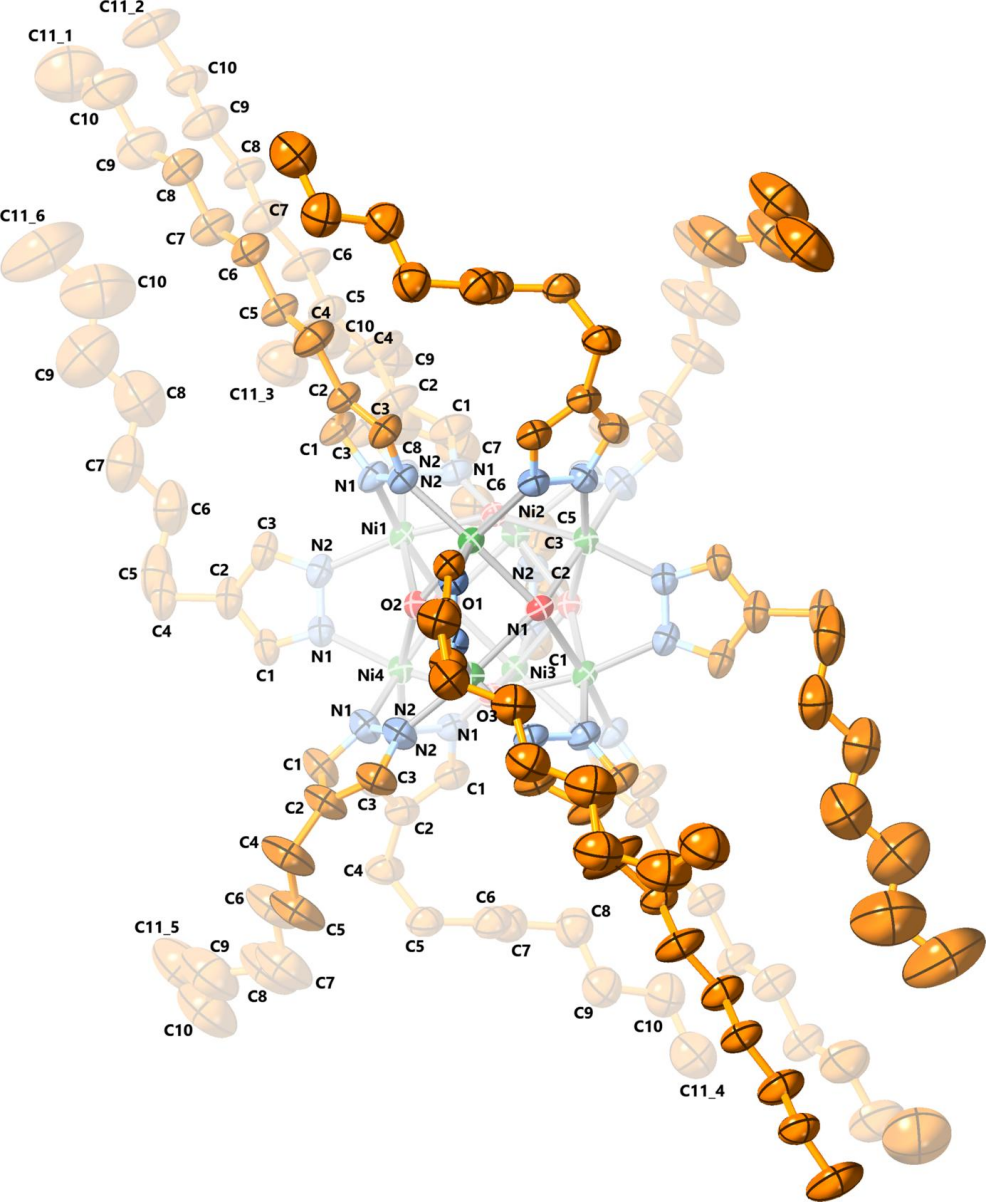
Displacement ellipsoid plot (50% probability) of the crystal structure of one [Ni_8_(μ_4_-OH)_6_(μ-4-^*n*^Octpz)_12_]^2−^ unit of **3**. Only atoms within the asymmetric unit are labeled.

**Figure 6:**
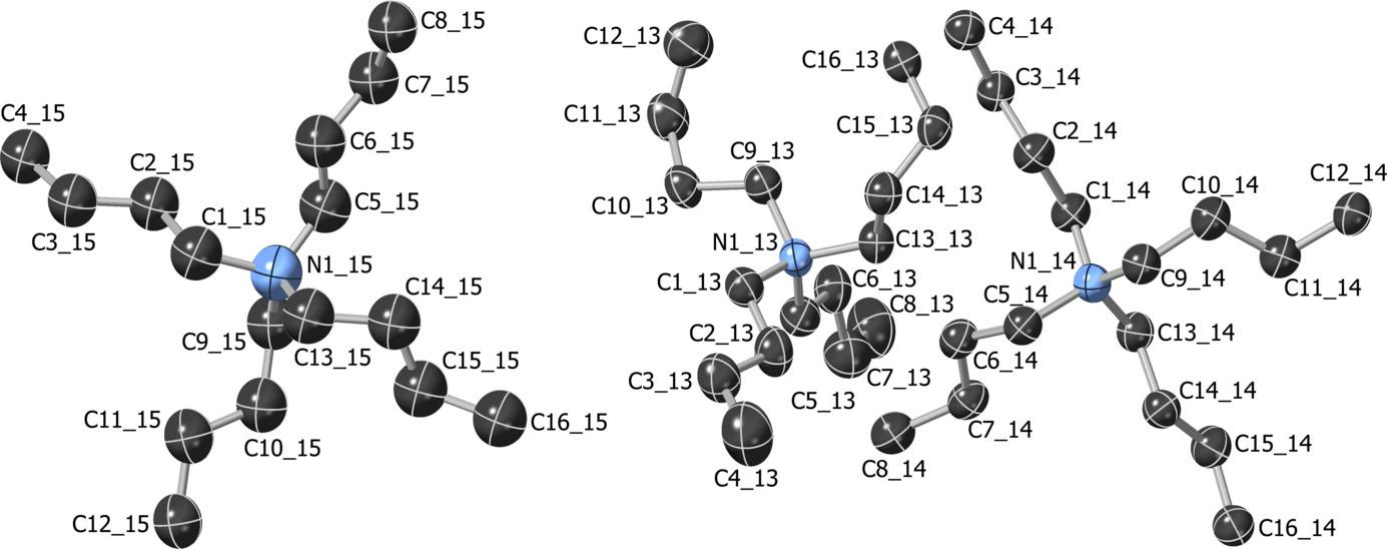
Displacement ellipsoid plot (50% probability) of the Bu_4_N^+^ counter-ions in **3**. Disorder and H atoms are omitted for clarity.

The crystal structure of **3** (triclinic, *P* 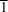 ) contains two crystallographically independent Ni_8_ complexes, both located around inversion centers (Figs. 4 ▸ and 5 ▸). Six and four of the twelve *n*-octyl chains of the two different units are disordered over two positions. The Bu_4_N^+^ cations are disordered around inversion centers, and one of them has additional disorder (Fig. 6 ▸). The Ni_8_ cube has an even lower symmetry than in **1** and **2**, with Ni⋯Ni distances of 2.975 (2)–3.005 (2) Å [average: 2.989 (2) Å] and Ni—Ni—Ni angles ranging between 89.23 (4) and 90.62 (4)° (Table 1 ▸). Pyrazolate rings on opposite sides of [Ni(4-^*n*^Octpz)]_4_ faces are also less symmetrically arranged, with corresponding fold and twist angles ranging from 60.3 (15) to 105.9 (2)° and from 1.7 (3) to 11.9 (4)°, respectively. The Ni—O and Ni—N bond lengths, O—Ni—N angles and distances between μ_4_-OH groups and Ni_4_ faces are again similar to the ones observed in **1** and **2** (Table 1 ▸).

## 3. Supra­molecular features

**Figure 7:**
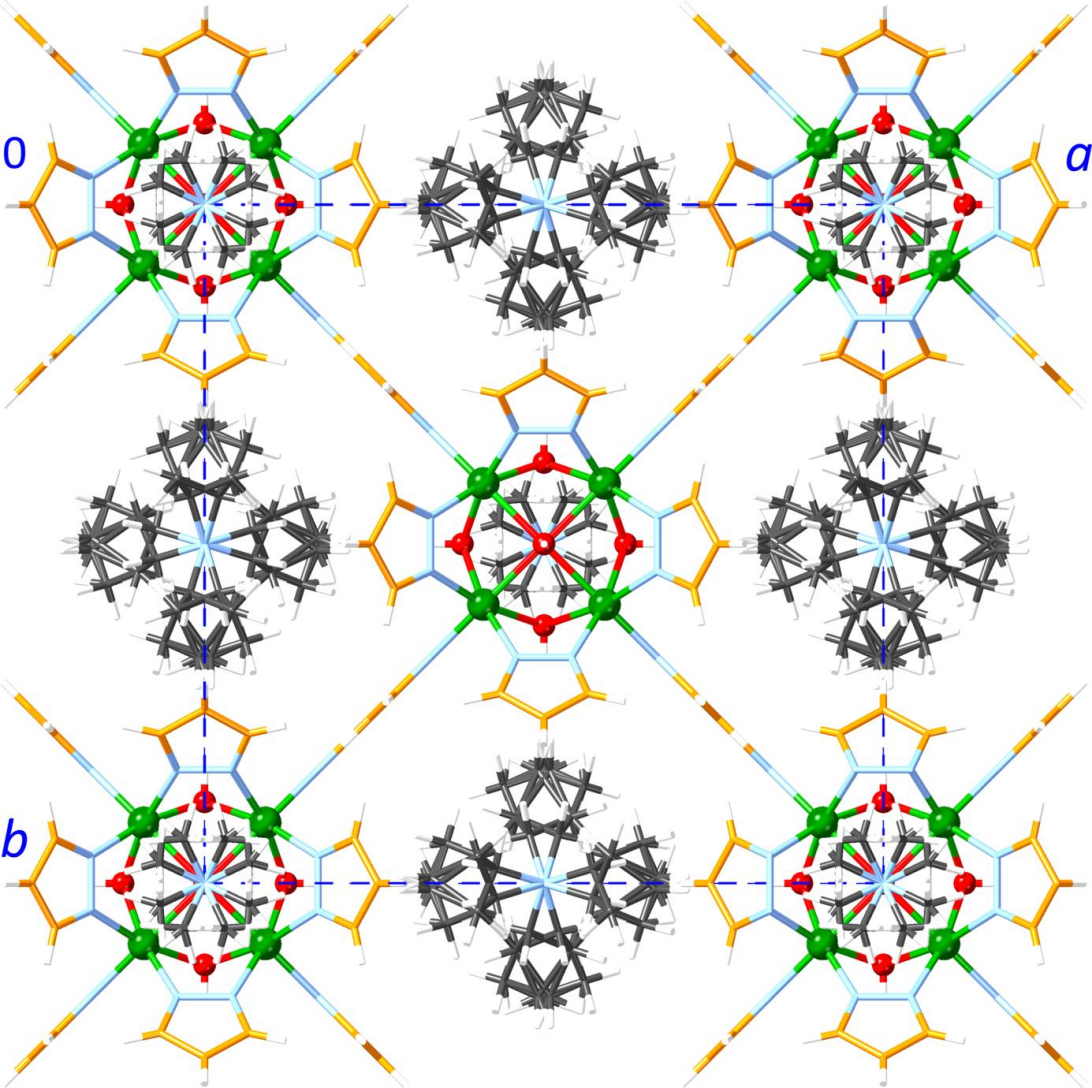
Packing diagram of **1**. Color scheme: green – Ni; red – O; blue – N; orange – C (pyrazolate ligand); black – C (counter-ions); white – H.

**Figure 8:**
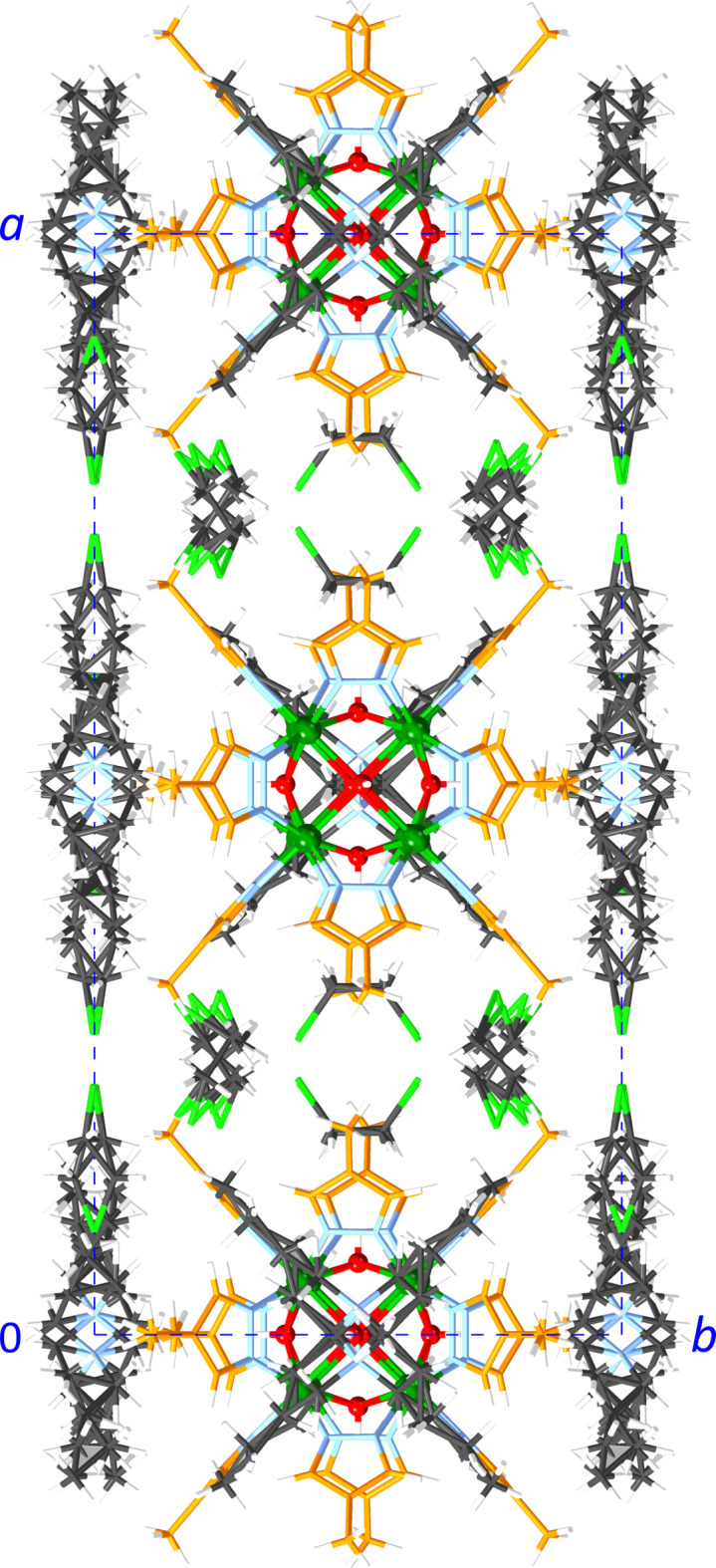
Packing diagram of **2**. Color scheme: green – Ni; red – O; blue – N; orange – C (pyrazolate ligand); black – C (counter-ions); neon-green – Cl; white – H.

**Figure 9:**
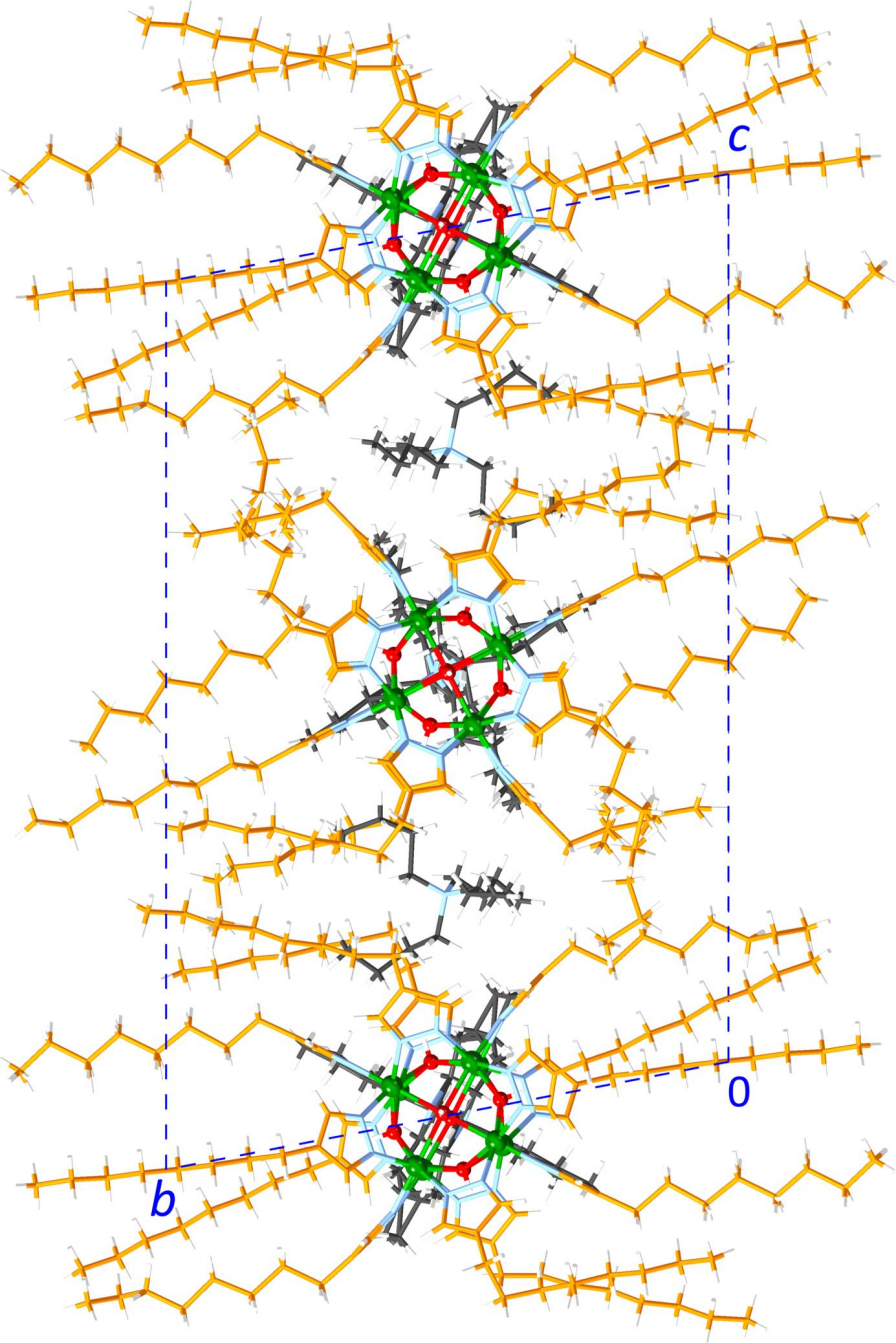
Packing diagram of **3** (disorder omitted). Color scheme: green – Ni; red – O; blue – N; orange – C (pyrazolate ligand); black – C (counter-ions); white – H.

In **1**, the Ni_8_ units are lined up in columns along the *z* axis with Ni_8_⋯Ni_8_ centroid–centroid distances of 12.4112 (4) Å (*i.e*., the *z*-axis length), alternating with the disordered Me_4_N^+^ ions (Fig. 7 ▸). This packing pattern creates channels along the *z* axis, which are filled with the disordered Bu_4_N^+^ ions. In **2**, the Ni_8_ units are also lined up parallel to the *z* axis with Ni_8_⋯Ni_8_ centroid–centroid distances of 14.5141 (9) Å (*z* axis), alternating with one of the two disordered Bu_4_N^+^ ions (Fig. 8 ▸). The Ni_8_ units are similarly lined up parallel to the *y* axis with Ni_8_⋯Ni_8_ centroid–centroid distances of 14.5762 (9) Å (*y* axis), alternating with the other disordered Bu_4_N^+^ ion. The remaining inter­stitial spaces are filled with 1,2-di­chloro­ethane solvent mol­ecules. In **3**, the two crystallographically unique Ni_8_ units are each lined up parallel to the *x* axis with Ni_8_⋯Ni_8_ centroid–centroid distances of 13.973 (8) Å (*x* axis), alternating with disordered Bu_4_N^+^ ions (Fig. 9 ▸). The inter­stitial spaces are filled with the non-disordered Bu_4_N^+^ ions. In all three structures, the Ni_8_ units are sufficiently spaced out by the counter-ions (and solvent mol­ecules in the case of **2**) to prevent any aromatic inter­actions.

## 4. Database survey

Two crystal structures containing the [Ni_8_(μ_4_-OH)_6_(μ-pz)_12_]^2−^ unit have been published previously (Al Isawi *et al.*, 2018 ▸; Xu *et al.*, 2008 ▸). Both have much lower symmetry (inversion center only, in *P*2_1_/*c* and *P* 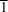 lattices, respectively) than in **1** (*I*4/*mmm*), and have either two Bu_4_N^+^ or an [Ni(bma)(H_2_O)_3_]^2+^ [bma = bis­(2-benzimidazolylmeth­yl)amine] complex as counter-ions. The presence of one Me_4_N^+^ counter-ion in **1**, which is extensively disordered to accommodate the various symmetry elements it is located on (one crystallographically unique methyl group in sixteen symmetry-equivalent positions), might account for achieving such high symmetry. Indeed, none of the other known discrete [Ni_8_(μ_4_-OH)_6_(μ-4-*R*pz)_12_]^2−^ (*R* = Me, ^*n*^Oct, Cl, Br, I) complexes display a perfectly cubic Ni_8_ core in their crystal lattice (Wang *et al.*, 2016 ▸), let alone a perfectly cubic [Ni_8_(μ_4_-OH)_6_(μ-4-*R*pz)_12_]^2−^ unit (Table 1 ▸). Apparently, it is only in three-dimensional metal–organic frameworks, where the individual [Ni_8_(μ_4_-OH)_6_(μ-4-*R*pz)_12_]^2−^ units are fully inter­connected, that full cubic symmetry of the complex can be achieved (Lv *et al.*, 2017 ▸; Quartapelle Procopio *et al.*, 2011 ▸; Masciocchi *et al.*, 2010 ▸). In these cubic lattices (*Pm* 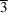 *m* or *Fm* 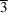 *m*), all crystallographic symmetry elements are imposed onto the metal complex.

A related low-symmetry structure of [Ni_8_(μ_4_-OH)_6_(μ-3(5)-Ph-4-CNpz)_8_(μ-Cl)_4_]^2−^ is also known (*C*_2_ axis only, considering the metal complex alone) (Kromer *et al.*, 2023 ▸). The presence of a bulky phenyl substituent at the pyrazole 3(5)-position apparently prevents all Ni_4_ faces from accommodating four pyrazolate ligands. Therefore, one chloride replaces a pyrazolate ligand on the four faces collinear with the *C*_2_ axis, and two chlorides replace two pyrazolates on the two faces centered on the *C*_2_ axis.

## 5. Synthesis and crystallization

Compounds **1**–**3** were synthesized as their tetra-*n*-butyl­ammonium salts by self-assembly in tetra­hydro­furan at room temperature using Ni(NO_3_)_2_·6H_2_O, pyrazole ligand [4-*R*pzH; *R* = H (**1**), methyl (**2**) or *n*-octyl (**3**)] and Bu_4_NOH (55% in water) in an 8:12:18 molar ratio, according to the published procedure (Al Isawi *et al.*, 2018 ▸). Single-crystals were grown by slow evaporation of a tetra­hydro­furan solution containing a small amount of Me_4_NOH (**1**), from toluene/1,2-di­chloro­ethane (**2**), or toluene/*n*-butanol (**3**).

## 6. Refinement

Crystal data, data collection and structure refinement details are summarized in Table 2 ▸.

**1**: A Bu_4_N^+^ cation is extensively disordered by symmetry. The nitro­gen atom is located at the inter­section of two mirror planes and twofold axes. The C atoms of the single unique butyl chain are located in general positions and are disordered over two alternative sets for each cation. Overlap of the butyl chain with its counterpart from a neighboring cation further reduces the occupancy by one quarter (the entire cation site is half-occupied). The C—C bonds of C6—C7 and C7—C8 were restrained to target values [1.53 (2) and 1.55 (2) Å]. An Me_4_N^+^ ion is disordered over four alternative orientations, with the single unique methyl group being located in sixteen symmetry-equivalent positions. Hydroxyl H-atom positions within the anion were refined and O—H distances were restrained to 0.84 (2) Å.

**2**: The two Bu_4_N^+^ cations exhibit twofold rotational symmetry and are disordered across a crystallographic mirror plane. Equivalent bonds in all cation moieties were restrained to be similar in length (SADI restraints). The two crystallographically independent half cations were also restrained to have similar geometries (SAME restraint). *U*_ij_ components of ADPs for disordered atoms closer to each other than 2.0 Å were restrained to be similar. Some evidence for additional disorder is apparent for the cation of N13, but is not well enough resolved for unambiguous refinement.

Four 1,2-di­chloro­ethane mol­ecules are present in the asymmetric unit. All were refined to be disordered across twofold rotational axes. All disordered moieties were restrained to have similar geometries (SAME restraint) and one of the C—C bonds (C31—C32) was restrained to a target value of 1.55 (2) Å. *U*_ij_ components of ADPs for disordered atoms closer to each other than 2.0 Å were restrained to be similar. Two sites were refined to be partially occupied (of Cl3/Cl4 and Cl5/Cl6). Subject to the above conditions the occupancy rates refined to two times 0.369 (6) and two times 0.430 (5), respectively.

**3**: The crystal under investigation was found to be a non-merohedric twin. The orientation matrices for the two components were identified using the program *CELL_NOW* (Sheldrick, 2008 ▸), with the two components being related by a 180° rotation around the reciprocal *b*-axis. The two components were integrated using *SAINT* and corrected for absorption using *TWINABS* (Sheldrick, 2012 ▸), resulting in the following statistics:

27457 data (13708 unique) involve domain 1 only, mean *I*/σ 8.9

27508 data (13715 unique) involve domain 2 only, mean *I*/σ 8.1

57229 data (24936 unique) involve 2 domains, mean *I*/σ 11.4

1 data (1 unique) involve 3 domains, mean *I*/σ 2.7

The exact twin matrix identified by the integration program was found to be:

–0.99999 0.00004 −0.00000

0.51610 1.00001 0.24406

–0.00001 −0.00019 −1.00002

The structure was solved using dual-space methods using all non-overlapping reflections of both components. The structure was refined using HKLF 5 format data, with all reflections of both components (including overlapping reflections), resulting in a BASF value of 0.4564 (7). The *R*_int_ value given is for all reflections and is based on agreement between observed single and composite intensities and those calculated from refined unique intensities and twin fractions (*TWINABS*).

Some of the alkyl chains were refined as twofold disordered. The geometries of all fully occupied and major moiety pyrazolate ligands were restrained to be similar to each other, and disordered major and minor moieties were restrained to have similar geometries. *U*_ij_ components of ADPs for disordered atoms closer to each other than 2.0 Å were restrained to be similar. Subject to these conditions the occupancy ratio refined to 0.713 (8)/0.287 (8) (C5–C11 residue 3), 0.507 (8)/0.493 (8) (C5–C11 residue 4), 0.518 (9)/0.482 (9) (C5–C11 residue 5), 0.450 (13)/0.550 (13) (C6–C11 residue 7), and 0.524 (11)/0.476 (11) (C5–C11 residue 10). Two Bu_4_N^+^ cations are disordered around inversion centers (residues 14 and 15). One of them is additionally disordered (residue 15). The geometries of all partially occupied Bu_4_N^+^ cations were restrained to be similar to that of the single non-disordered cation (residue 13). All N—C bond lengths in all cations were restrained to be similar to each other. *U*_ij_ components of ADPs for disordered atoms closer to each other than 2.0 Å were restrained to be similar. Subject to these conditions the occupancy ratio for the additionally split cation refined to two times 0.257 (5) and two times 0.243 (5). Hydroxyl H atom positions were refined and O—H distances were restrained to 0.84 (2) Å. All hydroxyl H—O—Ni angles were restrained (*via* the H⋯Ni distances) to be similar to each other.

## Supplementary Material

Crystal structure: contains datablock(s) 1, 2, 3, global. DOI: 10.1107/S2056989023010101/xi2028sup1.cif

Structure factors: contains datablock(s) 1. DOI: 10.1107/S2056989023010101/xi20281sup2.hkl

Structure factors: contains datablock(s) 2. DOI: 10.1107/S2056989023010101/xi20282sup6.hkl

Structure factors: contains datablock(s) 3. DOI: 10.1107/S2056989023010101/xi20283sup4.hkl

Supporting information file. DOI: 10.1107/S2056989023010101/xi20283sup8.cml

CCDC references: 2309528, 2309527, 2309526

Additional supporting information:  crystallographic information; 3D view; checkCIF report

## Figures and Tables

**Figure 5:**
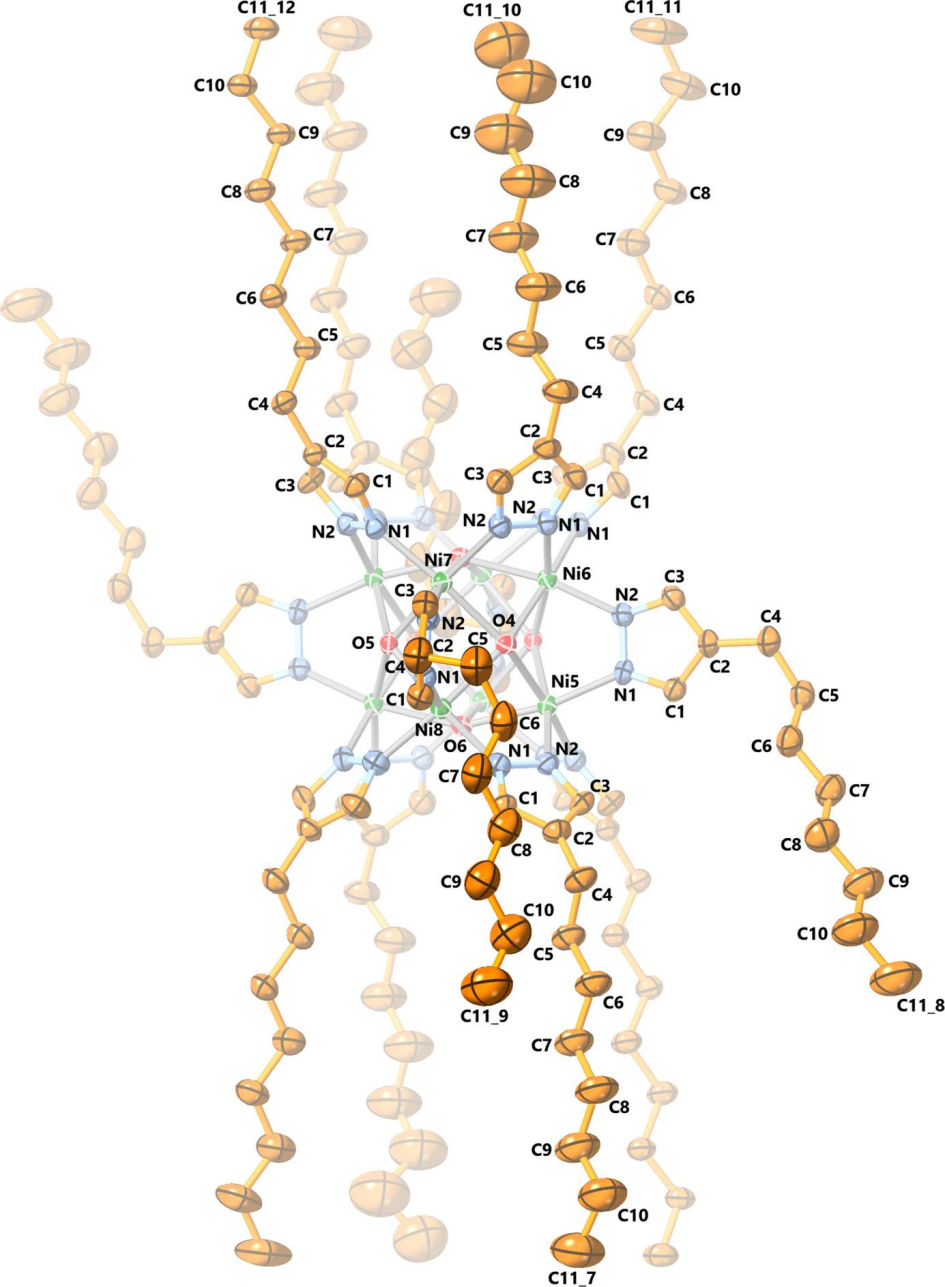
Displacement ellipsoid plot (50% probability) of the crystal structure of the other [Ni_8_(μ_4_-OH)_6_(μ-4-^*n*^Octpz)_12_]^2−^ unit of **3**. Only atoms within the asymmetric unit are labeled.

**Table 1:** Comparison of structural features (Å, °) determined by X-ray diffraction for various [Ni_8_(μ_4_-OH)_6_(μ-4-*R*pz)_12_]^2−^ species, as well as the [Ni_8_(μ-OH)_6_(μ-3(5)-Ph-4-CNpz)_8_(μ-Cl)_4_]^2−^ analog Refcodes are shown for previously published compounds (CSD version 2023.2.0; last update November 2023; Groom *et al.*, 2016 ▸). Similarly to **3**, TAXKOV contains two crystallographically independent complexes in the asymmetric unit. Only symmetry elements that apply to the metal complex are shown. TPP = tetra­phenyl­porphyrin; PMDI = pyromellitic di­imide.

Ni_8_ complex	Space group	Symmetry elements	Ni—O	Ni—N	Ni···Ni	Ni—Ni—Ni	O—Ni—N	O···(Ni_4_ plane)
pz (**1**)	*I*4/*mmm*	*C*_4_,*C*_2_,4⊥*C*_2_,*S*_4_,*σ*_h_,2*σ*_v_,2*σ*_d_,*i*	2.1710 (12), 2.1742 (9) ave: 2.173 (1)	2.013 (3), 2.027 (2) ave: 2.022 (2)	2.9826 (8)	90	169.54 (15), 169.89 (11) ave: 169.8 (2)	0.513 (3), 0.515 (3) ave: 0.514 (3)
4-Mepz (**2**)	*Pmna*	*C*_2_,*σ*_h_,*i*	2.1735 (12)–2.181 (3) ave: 2.177 (3)	2.019 (4)–2.031 (4) ave: 2.026 (4)	2.9837 (12)–2.9988 (10) ave: 2.993 (1)	89.92 (3)–90.08 (3) ave: 90.00	168.91 (16)–169.78 (17) ave: 169.3 (2)	0.491 (4)–0.519 (4) ave: 0.509 (4)
4-*^*n*^* Octpz (**3**)	**P* 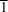 *	*i*	2.153 (3)–2.197 (3) ave: 2.174 (3) 2.156 (3)–2.187 (3) ave: 2.175 (3)	2.013 (4)–2.042 (5) ave: 2.024 (5) 2.012 (4)–2.042 (4) ave: 2.026 (4)	2.975 (2)–3.0049 (17) ave: 2.987 (2) 2.986 (2)–3.0013 (16) ave: 2.990 (2)	89.23 (4)–90.62 (4) ave: 90.00 89.35 (3)–90.55 (3) ave: 90.00	169.23 (14)–170.33 (14) ave: 169.7 (2) 169.19 (13)–170.41 (14) ave: 169.7 (2)	0.512 (3)–0.518 (3) ave: 0.516 (3) 0.498 (3)–0.527 (3) ave: 0.513 (3)
pz (TAXKOV)	*P*2_1_/*c*	*i*	2.1466 (7)–2.2031 (7) ave: 2.1724 (7) 2.1479 (7)–2.1919 (7) ave: 2.1730 (7)	2.0051 (9)–2.0355 (9) ave: 2.0244 (9) 2.0126 (9)–2.0315 (9) ave: 2.0244 (9)	2.9628 (6)–3.0138 (6) ave: 2.9881 (6) 2.9725 (7)–3.0043 (6) ave: 2.9890 (6)	88.59 (2)–91.36 (2) ave: 90.00 89.41 (2)–90.62 (2) ave: 90.00	167.50 (4)–169.47 (4) ave: 168.58 (4) 167.08 (4)–169.39 (4) ave: 168.64 (4)	0.4942 (6)–0.5123 (8) ave: 0.5053 (7) 0.4790 (6)–0.5210 (7) ave: 0.5044 (7)
pz (TOPGEL)	**P* 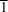 *	*i*	2.148 (3)–2.193 (4) ave: 2.172 (4)	2.009 (6)–2.041 (5) ave: 2.024 (6)	2.9576 (11)–3.0017 (11) ave: 2.986 (1)	88.77 (3)–91.43 (3) ave: 90.00	167.81 (16)–171.0 (2) ave: 169.3 (2)	0.506 (4)–0.513 (3) ave: 0.509 (4)
4-Clpz (ELUPUY)	*P*4/*mnc*	*C*_4_,*C*_2_,4⊥*C*_2_,*S*_4_,*σ*_h_,*i*	2.166 (3)–2.1842 (13) ave: 2.177 (3)	2.028 (3)–2.045 (3) ave: 2.034 (3)	2.9799 (11)–3.0006 (8) ave: 2.994 (1)	90.00	169.60 (14), 169.98 (15) ave: 169.7 (2)	0.506 (4), 0.519 (5) ave: 0.510 (5)
4-Brpz (ELUQAF)	*Pbcn*	*C*_2_	2.137 (8)–2.218 (9) ave: 2.165 (9)	1.987 (11)–2.043 (11) ave: 2.022 (11)	2.958 (3)–2.989 (3) ave: 2.977 (3)	88.85 (7)–91.27 (7) ave: 90.00	167.5 (4)–171.6 (4) ave: 169.4 (4)	0.485 (7)–0.527 (9) ave: 0.505 (9)
4-Ipz (ELUQEJ)	*P*2_1_/*c*	*i*	2.104 (15)–2.234 (13) ave: 2.169 (15)	1.961 (19)–2.050 (19) ave: 2.017 (19)	2.953 (4)–3.005 (4) ave: 2.986 (4)	88.02 (10)–92.29 (11) ave: 90.00	165.6 (6)–171.2 (7) ave: 168.7 (7)	0.452 (12)–0.543 (18) ave: 0.493 (14)
TPP(pz)_4_ (BALNAG)	*Pm 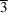 m*	3*C*_4_,4*C*_3_,6*C*_2_,4*S*_6_,3*S*_4_,3*σ*_h_,6*σ*_d_,*i*	2.20 (3)	2.07 (2)	2.798 (13)	90.00	174.1 (19)	0.97 (7)
(4-C≡Cpz)_2_ (HAFTOZ)	*Fm 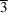 m*	3*C*_4_,4*C*_3_,6*C*_2_,4*S*_6_,3*S*_4_,3*σ*_h_,6*σ*_d_,*i*	2.239 (12)	2.074 (3)	2.825 (6)	90.00	173.8 (6)	1.01 (3)
(4-C_6_H_4_pz)_2_ (OKERAY)	*Fm 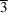 m*	3*C*_4_,4*C*_3_,6*C*_2_,4*S*_6_,3*S*_4_,3*σ*_h_,6*σ*_d_,*i*	2.155 (11)	2.093 (5)	2.921 (8)	90.00	174.8 (9)	0.62 (4)
PMDI(pz)_2_ (OKEREC)	*Fm 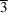 m*	3*C*_4_,4*C*_3_,6*C*_2_,4*S*_6_,3*S*_4_,3*σ*_h_,6*σ*_d_,*i*	2.082 (10)	2.006 (6)	2.885 (11)	90.00	169.6 (11)	0.41 (4)
3(5)-Ph-4-CNpz (VITHEP)	*C*2/*c*	*C*_2_	2.0723 (13)–2.224 (3) ave: 2.137 (3)	1.975 (5)–2.084 (5) ave: 2.025 (5)	2.8112 (10)–2.9878 (12) ave: 2.926 (1)	87.45 (3)–92.22 (3) ave: 90.00	168.82 (15)–170.70 (18) ave: 169.8 (2)	0.494 (5)–0.553 (4) ave: 0.531 (5)

**Table 2:** Experimental details

	(1)	(2)	(3)
Crystal data			
Chemical formula	(C_16_H_36_N)(C_4_H_12_N)[Ni_8_(C_3_H_3_N_2_)_12_(OH)_6_]	(C_16_H_36_N)_2_[Ni_8_(C_4_H_5_N_2_)_12_(OH)_6_]·7.196C_2_H_4_Cl_2_	(C_16_H_36_N)_2_[Ni_8_(C_11_H_19_N_2_)_12_(OH)_6_]
*M*_r_	1693.21	2739.84	3208.01
Crystal system, space group	Tetragonal, *I*4/*m* *m* *m*	Orthorhombic, *P* *m* *n* *a*	Triclinic, *P* 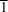
Temperature (K)	150	150	150
*a*, *b*, *c* (Å)	16.6921 (4), 16.6921 (4), 12.4112 (4)	30.3900 (18), 14.5762 (9), 14.5141 (9)	13.973 (8), 20.765 (10), 31.873 (14)
α, β, γ (°)	90, 90, 90	90, 90, 90	99.896 (17), 94.798 (17), 99.10 (2)
*V* (Å^3^)	3458.09 (18)	6429.3 (7)	8938 (8)
*Z*	2	2	2
Radiation type	Cu *K*α	Cu *K*α	Cu *K*α
μ (mm^−1^)	2.86	4.44	1.33
Crystal size (mm)	0.25 × 0.23 × 0.19	0.36 × 0.33 × 0.26	0.22 × 0.08 × 0.03

Data collection			
Diffractometer	Bruker AXS D8 Quest with PhotonIII_C14 charge-integrating and photon counting pixel array detector	Bruker AXS D8 Quest with PhotonIII_C14 charge-integrating and photon counting pixel array detector	Bruker AXS D8 Quest with PhotonIII_C14 charge-integrating and photon counting pixel array detector
Absorption correction	Multi-scan (*SADABS*; Krause *et al.*, 2015 ▸)	Multi-scan (*SADABS*; Krause *et al.*, 2015 ▸)	Multi-scan (*TWINABS*; Sheldrick, 2012 ▸)
*T*_min_, *T*_max_	0.597, 0.754	0.624, 0.754	0.594, 0.753
No. of measured, independent and observed [*I* > 2σ(*I*)] reflections	10921, 1094, 1052	73571, 7078, 6512	110848, 52094, 37936
*R*_int_	0.052	0.045	0.071
(sin θ/λ)_max_ (Å^−1^)	0.638	0.638	0.612

Refinement			
*R*[*F* ^2^ > 2σ(*F* ^2^)], *wR*(*F* ^2^), *S*	0.044, 0.113, 1.12	0.081, 0.254, 1.10	0.066, 0.185, 1.05
No. of reflections	1094	7078	52094
No. of parameters	109	517	2507
No. of restraints	4	271	5486
H-atom treatment	H atoms treated by a mixture of independent and constrained refinement	H atoms treated by a mixture of independent and constrained refinement	H atoms treated by a mixture of independent and constrained refinement
Δρ_max_, Δρ_min_ (e Å^−3^)	0.56, −0.40	1.14, −0.89	0.73, −0.52

Computer programs: *APEX4* (Bruker, 2021 ▸), *APEX3* and *SAINT* (Bruker, 2020 ▸), SHELXT (Sheldrick, 2015*a* ▸), *SHELXL2018/3* (Sheldrick, 2015*b* ▸), *ShelXle* (Hübschle *et al.*, 2011 ▸), and *CrystalMaker* (Palmer, 2007 ▸).

## supplementary crystallographic information

### Tetrabutylazanium tetramethylazanium hexa-µ4-hydroxido-dodeca-µ2-pyrazolato-hexahedro-octanickel (1) Crystal data

**Table:**

(C_16_H_36_N)(C_4_H_12_N)[Ni_8_(C_3_H_3_N_2_)_12_(OH)_6_]	*D*_x_ = 1.626 Mg m^−^^3^
*M**_r_* = 1693.21	Cu *K*α radiation, λ = 1.54178 Å
Tetragonal, *I*4/*m**m**m*	Cell parameters from 8487 reflections
*a* = 16.6921 (4) Å	θ = 6.9–79.5°
*c* = 12.4112 (4) Å	µ = 2.86 mm^−^^1^
*V* = 3458.09 (18) Å^3^	*T* = 150 K
*Z* = 2	Block, blue
*F*(000) = 1760	0.25 × 0.23 × 0.19 mm

### Tetrabutylazanium tetramethylazanium hexa-µ4-hydroxido-dodeca-µ2-pyrazolato-hexahedro-octanickel (1) Data collection

**Table:**

Bruker AXS D8 Quest diffractometer with PhotonIII_C14 charge-integrating and photon counting pixel array detector	1094 independent reflections
Radiation source: I-mu-S microsource X-ray tube	1052 reflections with *I* > 2σ(*I*)
Laterally graded multilayer (Goebel) mirror monochromator	*R*_int_ = 0.052
Detector resolution: 7.4074 pixels mm^-1^	θ_max_ = 79.6°, θ_min_ = 6.9°
ω and phi scans	*h* = −21→21
Absorption correction: multi-scan (SADABS; Krause *et al.*, 2015)	*k* = −19→15
*T*_min_ = 0.597, *T*_max_ = 0.754	*l* = −15→15
10921 measured reflections	

### Tetrabutylazanium tetramethylazanium hexa-µ4-hydroxido-dodeca-µ2-pyrazolato-hexahedro-octanickel (1) Refinement

**Table:**

Refinement on *F*^2^	Secondary atom site location: difference Fourier map
Least-squares matrix: full	Hydrogen site location: mixed
*R*[*F*^2^ > 2σ(*F*^2^)] = 0.044	H atoms treated by a mixture of independent and constrained refinement
*wR*(*F*^2^) = 0.113	*w* = 1/[σ^2^(*F*_o_^2^) + (0.0406*P*)^2^ + 14.3393*P*] where *P* = (*F*_o_^2^ + 2*F*_c_^2^)/3
*S* = 1.12	(Δ/σ)_max_ < 0.001
1094 reflections	Δρ_max_ = 0.56 e Å^−^^3^
109 parameters	Δρ_min_ = −0.40 e Å^−^^3^
4 restraints	Extinction correction: *SHELXL2018/3* (Sheldrick, 2015b), Fc^*^=kFc[1+0.001xFc^2^λ^3^/sin(2θ)]^-1/4^
Primary atom site location: dual	Extinction coefficient: 0.00094 (14)

### Tetrabutylazanium tetramethylazanium hexa-µ4-hydroxido-dodeca-µ2-pyrazolato-hexahedro-octanickel (1) Special details

**Table:**

Geometry. All esds (except the esd in the dihedral angle between two l.s. planes) are estimated using the full covariance matrix. The cell esds are taken into account individually in the estimation of esds in distances, angles and torsion angles; correlations between esds in cell parameters are only used when they are defined by crystal symmetry. An approximate (isotropic) treatment of cell esds is used for estimating esds involving l.s. planes.
Refinement. A Bu_4_N^+^ cation is extensively disordered by symmetry. The nitrogen atom is located at the intersection of two mirror planes and two-fold axes. The C atoms of the single unique butyl chain are located in general positions and are disordered over two alternative sets for each cation. Overlap of the butyl chain with is counterpart from a neighboring cation further reduces the occupancy by one quarter (the entire cation site is half-occupied). The C–C bonds of C6–C7 and C7–C8 were restrained to target values (1.53 (2) and 1.55 (2) Å). A Me_4_N^+^ ion is disordered over four alternative orientations, with the single unique methyl group being located in sixteen symmetry-equivalent positions. Hydroxyl H atom positions within the anion were refined and O–H distances were restrained to 0.84 (2) Å.

### Tetrabutylazanium tetramethylazanium hexa-µ4-hydroxido-dodeca-µ2-pyrazolato-hexahedro-octanickel (1) Fractional atomic coordinates and isotropic or equivalent isotropic displacement parameters (Å^2^)

**Table:**

	*x*	*y*	*z*	*U*_iso_*/*U*_eq_	Occ. (<1)
Ni1	0.41066 (2)	0.41066 (2)	0.12052 (4)	0.0213 (3)	
O1	0.500000	0.3798 (2)	0.000000	0.0209 (7)	
H1	0.500000	0.3298 (12)	0.000000	0.031*	
O2	0.500000	0.500000	0.1620 (4)	0.0181 (9)	
H2	0.500000	0.500000	0.2297 (17)	0.027*	
N1	0.33287 (13)	0.33287 (13)	0.0541 (2)	0.0353 (8)	
N2	0.45968 (14)	0.33272 (13)	0.22698 (17)	0.0299 (5)	
C1	0.27880 (19)	0.27880 (19)	0.0875 (4)	0.0668 (19)	
H1A	0.266967	0.266967	0.160683	0.080*	
C2	0.2431 (3)	0.2431 (3)	0.000000	0.094 (4)	
H2A	0.202853	0.202853	0.000000	0.113*	
C3	0.4349 (2)	0.2795 (2)	0.3003 (3)	0.0540 (9)	
H3	0.380575	0.267830	0.316641	0.065*	
C4	0.500000	0.2442 (4)	0.3482 (5)	0.079 (2)	
H4	0.500001	0.204215	0.402647	0.095*	
N3	0.500000	0.000000	0.250000	0.031 (3)	0.5
C5	0.4783 (6)	0.0721 (7)	0.1826 (9)	0.036 (3)	0.25
H5A	0.468859	0.117955	0.231542	0.043*	0.25
H5B	0.427289	0.060735	0.144993	0.043*	0.25
C6	0.5403 (9)	0.0970 (8)	0.0990 (12)	0.049 (3)	0.25
H6A	0.552850	0.050796	0.051982	0.058*	0.25
H6B	0.590225	0.113460	0.135851	0.058*	0.25
C7	0.509 (2)	0.1665 (8)	0.0300 (11)	0.051 (6)	0.25
H7A	0.554558	0.188280	−0.012533	0.062*	0.25
H7B	0.490304	0.209624	0.078433	0.062*	0.25
C8	0.4406 (15)	0.1440 (12)	−0.0483 (16)	0.092 (7)	0.25
H8A	0.428498	0.189868	−0.094870	0.137*	0.25
H8B	0.392763	0.129637	−0.006811	0.137*	0.25
H8C	0.457069	0.098365	−0.092701	0.137*	0.25
N4	0.500000	0.500000	0.500000	0.087 (5)	
C9	0.4686 (14)	0.5660 (12)	0.4394 (18)	0.127 (13)	0.25
H9A	0.482964	0.616355	0.474958	0.191*	0.25
H9B	0.410149	0.561458	0.435149	0.191*	0.25
H9C	0.491223	0.565160	0.366504	0.191*	0.25

### Tetrabutylazanium tetramethylazanium hexa-µ4-hydroxido-dodeca-µ2-pyrazolato-hexahedro-octanickel (1) Atomic displacement parameters (Å^2^)

**Table:**

	*U*^11^	*U*^22^	*U*^33^	*U*^12^	*U*^13^	*U*^23^
Ni1	0.0237 (3)	0.0237 (3)	0.0164 (4)	−0.0053 (2)	0.00026 (15)	0.00026 (15)
O1	0.0300 (18)	0.0152 (15)	0.0174 (15)	0.000	0.000	0.000
O2	0.0188 (13)	0.0188 (13)	0.017 (2)	0.000	0.000	0.000
N1	0.0427 (12)	0.0427 (12)	0.0204 (16)	−0.0218 (16)	−0.0004 (9)	−0.0004 (9)
N2	0.0372 (12)	0.0268 (11)	0.0257 (11)	−0.0035 (9)	0.0009 (9)	0.0067 (9)
C1	0.087 (3)	0.087 (3)	0.026 (2)	−0.068 (4)	0.0009 (13)	0.0009 (13)
C2	0.126 (7)	0.126 (7)	0.030 (3)	−0.107 (7)	0.000	0.000
C3	0.054 (2)	0.057 (2)	0.0513 (19)	−0.0084 (17)	0.0072 (17)	0.0307 (17)
C4	0.076 (4)	0.085 (5)	0.076 (4)	0.000	0.000	0.059 (4)
N3	0.032 (4)	0.032 (4)	0.029 (6)	0.000	0.000	0.000
C5	0.042 (8)	0.035 (5)	0.031 (5)	−0.001 (4)	−0.005 (4)	0.001 (4)
C6	0.056 (8)	0.034 (7)	0.055 (8)	−0.007 (6)	−0.003 (7)	0.006 (6)
C7	0.076 (18)	0.034 (5)	0.044 (7)	0.000 (11)	0.018 (15)	0.011 (4)
C8	0.14 (2)	0.054 (12)	0.080 (13)	−0.004 (13)	−0.021 (15)	0.031 (11)
N4	0.090 (8)	0.090 (8)	0.081 (12)	0.000	0.000	0.000
C9	0.13 (3)	0.080 (14)	0.18 (2)	−0.004 (14)	−0.005 (16)	0.050 (16)

### Tetrabutylazanium tetramethylazanium hexa-µ4-hydroxido-dodeca-µ2-pyrazolato-hexahedro-octanickel (1) Geometric parameters (Å, º)

**Table:**

Ni1—N1	2.013 (3)	C3—H3	0.9500
Ni1—N2	2.027 (2)	C4—H4	0.9500
Ni1—N2^i^	2.027 (2)	N3—C5	1.509 (11)
Ni1—O2	2.1710 (12)	C5—C6	1.523 (18)
Ni1—O1^ii^	2.1742 (9)	C5—H5A	0.9900
Ni1—O1	2.1742 (9)	C5—H5B	0.9900
Ni1—Ni1^iii^	2.9826 (8)	C6—C7	1.531 (15)
Ni1—Ni1^iv^	2.9826 (8)	C6—H6A	0.9900
Ni1—Ni1^v^	2.9916 (11)	C6—H6B	0.9900
O1—H1	0.83 (2)	C7—C8	1.55 (2)
O2—H2	0.84 (2)	C7—H7A	0.9900
N1—C1	1.342 (5)	C7—H7B	0.9900
N1—N1^v^	1.343 (6)	C8—H8A	0.9800
N2—C3	1.337 (4)	C8—H8B	0.9800
N2—N2^vi^	1.346 (5)	C8—H8C	0.9800
C1—C2	1.375 (6)	N4—C9	1.433 (19)
C1—H1A	0.9500	C9—H9A	0.9800
C2—H2A	0.9500	C9—H9B	0.9800
C3—C4	1.371 (5)	C9—H9C	0.9800
			
N1—Ni1—N2	96.51 (9)	Ni1^viii^—O2—H2	103.72 (12)
N1—Ni1—N2^i^	96.51 (9)	Ni1^iv^—O2—H2	103.72 (12)
N2—Ni1—N2^i^	95.35 (13)	Ni1^iii^—O2—H2	103.72 (12)
N1—Ni1—O2	169.54 (15)	Ni1—O2—H2	103.72 (12)
N2—Ni1—O2	90.51 (10)	C1—N1—N1^v^	108.0 (2)
N2^i^—Ni1—O2	90.51 (10)	C1—N1—Ni1	137.8 (3)
N1—Ni1—O1^ii^	90.44 (9)	N1^v^—N1—Ni1	114.18 (9)
N2—Ni1—O1^ii^	169.90 (11)	C3—N2—N2^vi^	108.0 (2)
N2^i^—Ni1—O1^ii^	91.11 (8)	C3—N2—Ni1	138.2 (2)
O2—Ni1—O1^ii^	81.65 (11)	N2^vi^—N2—Ni1	113.81 (6)
N1—Ni1—O1	90.44 (9)	N1—C1—C2	109.8 (4)
N2—Ni1—O1	91.11 (8)	N1—C1—H1A	125.1
N2^i^—Ni1—O1	169.90 (11)	C2—C1—H1A	125.1
O2—Ni1—O1	81.65 (11)	C1^v^—C2—C1	104.4 (6)
O1^ii^—Ni1—O1	81.48 (13)	C1^v^—C2—H2A	127.8
N1—Ni1—Ni1^iii^	130.17 (3)	C1—C2—H2A	127.8
N2—Ni1—Ni1^iii^	66.19 (6)	N2—C3—C4	109.6 (3)
N2^i^—Ni1—Ni1^iii^	129.93 (6)	N2—C3—H3	125.2
O2—Ni1—Ni1^iii^	46.61 (3)	C4—C3—H3	125.2
O1^ii^—Ni1—Ni1^iii^	103.71 (9)	C3^vi^—C4—C3	104.8 (5)
O1—Ni1—Ni1^iii^	46.69 (2)	C3^vi^—C4—H4	127.6
N1—Ni1—Ni1^iv^	130.17 (3)	C3—C4—H4	127.6
N2—Ni1—Ni1^iv^	129.93 (6)	N3—C5—C6	115.7 (9)
N2^i^—Ni1—Ni1^iv^	66.19 (6)	N3—C5—H5A	108.4
O2—Ni1—Ni1^iv^	46.61 (3)	C6—C5—H5A	108.4
O1^ii^—Ni1—Ni1^iv^	46.69 (2)	N3—C5—H5B	108.4
O1—Ni1—Ni1^iv^	103.71 (9)	C6—C5—H5B	108.4
Ni1^iii^—Ni1—Ni1^iv^	90.0	H5A—C5—H5B	107.4
N1—Ni1—Ni1^v^	65.82 (9)	C5—C6—C7	111.1 (15)
N2—Ni1—Ni1^v^	130.68 (6)	C5—C6—H6A	109.4
N2^i^—Ni1—Ni1^v^	130.69 (6)	C7—C6—H6A	109.4
O2—Ni1—Ni1^v^	103.72 (12)	C5—C6—H6B	109.4
O1^ii^—Ni1—Ni1^v^	46.53 (2)	C7—C6—H6B	109.4
O1—Ni1—Ni1^v^	46.53 (2)	H6A—C6—H6B	108.0
Ni1^iii^—Ni1—Ni1^v^	90.0	C6—C7—C8	114.6 (15)
Ni1^iv^—Ni1—Ni1^v^	90.0	C6—C7—H7A	108.6
Ni1^iii^—O1—Ni1^v^	152.57 (17)	C8—C7—H7A	108.6
Ni1^iii^—O1—Ni1^vii^	86.94 (5)	C6—C7—H7B	108.6
Ni1^v^—O1—Ni1^vii^	86.61 (5)	C8—C7—H7B	108.6
Ni1^iii^—O1—Ni1	86.61 (5)	H7A—C7—H7B	107.6
Ni1^v^—O1—Ni1	86.94 (5)	C7—C8—H8A	109.5
Ni1^vii^—O1—Ni1	152.57 (17)	C7—C8—H8B	109.5
Ni1^iii^—O1—H1	103.72 (9)	H8A—C8—H8B	109.5
Ni1^v^—O1—H1	103.72 (8)	C7—C8—H8C	109.5
Ni1^vii^—O1—H1	103.72 (9)	H8A—C8—H8C	109.5
Ni1—O1—H1	103.71 (8)	H8B—C8—H8C	109.5
Ni1^viii^—O2—Ni1^iv^	86.77 (5)	N4—C9—H9A	109.5
Ni1^viii^—O2—Ni1^iii^	86.77 (5)	N4—C9—H9B	109.5
Ni1^iv^—O2—Ni1^iii^	152.6 (2)	H9A—C9—H9B	109.5
Ni1^viii^—O2—Ni1	152.6 (2)	N4—C9—H9C	109.5
Ni1^iv^—O2—Ni1	86.78 (5)	H9A—C9—H9C	109.5
Ni1^iii^—O2—Ni1	86.78 (5)	H9B—C9—H9C	109.5
			
N1^v^—N1—C1—C2	0.000 (1)	Ni1—N2—C3—C4	−179.9 (4)
Ni1—N1—C1—C2	180.000 (1)	N2—C3—C4—C3^vi^	0.3 (8)
N1—C1—C2—C1^v^	0.000 (1)	N3—C5—C6—C7	175.7 (11)
N2^vi^—N2—C3—C4	−0.2 (5)	C5—C6—C7—C8	−70 (2)

Symmetry codes: (i) *y*, *x*, *z*; (ii) *y*, −*x*+1, −*z*; (iii) −*y*+1, *x*, *z*; (iv) *y*, −*x*+1, *z*; (v) *x*, *y*, −*z*; (vi) −*x*+1, *y*, *z*; (vii) −*y*+1, *x*, −*z*; (viii) −*x*+1, −*y*+1, *z*.

### Bis(tetrabutylazanium) hexa-µ4-hydroxido-dodeca-µ2-(4-methylpyrazolato)-hexahedro-octanickel 1,2-dichloroethane 7.196-solvate (2) Crystal data

**Table:**

(C_16_H_36_N)_2_[Ni_8_(C_4_H_5_N_2_)_12_(OH)_6_]·7.196C_2_H_4_Cl_2_	*D*_x_ = 1.415 Mg m^−^^3^
*M**_r_* = 2739.84	Cu *K*α radiation, λ = 1.54178 Å
Orthorhombic, *P**m**n**a*	Cell parameters from 9416 reflections
*a* = 30.3900 (18) Å	θ = 3.0–79.2°
*b* = 14.5762 (9) Å	µ = 4.44 mm^−^^1^
*c* = 14.5141 (9) Å	*T* = 150 K
*V* = 6429.3 (7) Å^3^	Block, blue
*Z* = 2	0.36 × 0.33 × 0.26 mm
*F*(000) = 2860	

### Bis(tetrabutylazanium) hexa-µ4-hydroxido-dodeca-µ2-(4-methylpyrazolato)-hexahedro-octanickel 1,2-dichloroethane 7.196-solvate (2) Data collection

**Table:**

Bruker AXS D8 Quest diffractometer with PhotonIII_C14 charge-integrating and photon counting pixel array detector	7078 independent reflections
Radiation source: I-mu-S microsource X-ray tube	6512 reflections with *I* > 2σ(*I*)
Laterally graded multilayer (Goebel) mirror monochromator	*R*_int_ = 0.045
Detector resolution: 7.4074 pixels mm^-1^	θ_max_ = 79.7°, θ_min_ = 5.2°
ω and phi scans	*h* = −30→38
Absorption correction: multi-scan (SADABS; Krause *et al.*, 2015)	*k* = −18→18
*T*_min_ = 0.624, *T*_max_ = 0.754	*l* = −18→14
73571 measured reflections	

### Bis(tetrabutylazanium) hexa-µ4-hydroxido-dodeca-µ2-(4-methylpyrazolato)-hexahedro-octanickel 1,2-dichloroethane 7.196-solvate (2) Refinement

**Table:**

Refinement on *F*^2^	Primary atom site location: structure-invariant direct methods
Least-squares matrix: full	Secondary atom site location: difference Fourier map
*R*[*F*^2^ > 2σ(*F*^2^)] = 0.081	Hydrogen site location: mixed
*wR*(*F*^2^) = 0.254	H atoms treated by a mixture of independent and constrained refinement
*S* = 1.10	*w* = 1/[σ^2^(*F*_o_^2^) + (0.1127*P*)^2^ + 18.8031*P*] where *P* = (*F*_o_^2^ + 2*F*_c_^2^)/3
7078 reflections	(Δ/σ)_max_ = 0.001
517 parameters	Δρ_max_ = 1.14 e Å^−^^3^
271 restraints	Δρ_min_ = −0.89 e Å^−^^3^

### Bis(tetrabutylazanium) hexa-µ4-hydroxido-dodeca-µ2-(4-methylpyrazolato)-hexahedro-octanickel 1,2-dichloroethane 7.196-solvate (2) Special details

**Table:**

Geometry. All esds (except the esd in the dihedral angle between two l.s. planes) are estimated using the full covariance matrix. The cell esds are taken into account individually in the estimation of esds in distances, angles and torsion angles; correlations between esds in cell parameters are only used when they are defined by crystal symmetry. An approximate (isotropic) treatment of cell esds is used for estimating esds involving l.s. planes.
Refinement. The two Bu_4_N^+^ cations exhibit twofold rotational symmetry and are disordered across a crystallographic mirror plane. Equivalent bonds in all cation moieties were restrained to be similar in length (SADI restraints). The two crystallographically independent half cations were also restrained to have similar geometries (SAME restraint). U_ij_ components of ADPs for disordered atoms closer to each other than 2.0 Å were restrained to be similar. Some evidence for additional disorder is apparent for the cation of N13, but is not well enough resolved for unambiguous refinement.Four 1,2-dichloroethane molecules are present in the asymmetric unit. All were refined to be disordered across twofold rotational axes. All disordered moieties were restrained to have similar geometries (SAME restraint) and one of the C–C bonds (C31–C32) was restrained to a target value of 1.55 (2) Å. U_ij_ components of ADPs for disordered atoms closer to each other than 2.0 Å were restrained to be similar. Two sites were refined to be partially occupied (of Cl3/Cl4 and Cl5/Cl6). Subject to the above conditions the occupancy rates refined to two times 0.369 (6) and two times 0.430 (5), respectively.

### Bis(tetrabutylazanium) hexa-µ4-hydroxido-dodeca-µ2-(4-methylpyrazolato)-hexahedro-octanickel 1,2-dichloroethane 7.196-solvate (2) Fractional atomic coordinates and isotropic or equivalent isotropic displacement parameters (Å^2^)

**Table:**

	*x*	*y*	*z*	*U*_iso_*/*U*_eq_	Occ. (<1)
Ni1	0.54913 (2)	0.60933 (5)	0.90395 (5)	0.0392 (2)	
Ni2	0.54909 (2)	0.59595 (5)	1.10979 (5)	0.0395 (2)	
O1	0.500000	0.6382 (3)	1.0090 (3)	0.0365 (8)	
H1O	0.500000	0.694 (2)	1.025 (5)	0.055*	
O2	0.56528 (13)	0.500000	1.000000	0.0365 (8)	
H2O	0.5929 (7)	0.500000	1.000000	0.055*	
O3	0.500000	0.4912 (3)	1.1385 (3)	0.0377 (8)	
H3O	0.500000	0.481 (5)	1.1961 (17)	0.057*	
N1	0.52200 (13)	0.7036 (3)	0.8182 (3)	0.0490 (9)	
N2	0.59131 (13)	0.6965 (3)	0.9663 (3)	0.0535 (10)	
N3	0.59201 (13)	0.6892 (3)	1.0584 (3)	0.0500 (9)	
N4	0.52186 (14)	0.6789 (3)	1.2059 (3)	0.0515 (9)	
N5	0.59369 (14)	0.5578 (3)	0.8134 (3)	0.0517 (9)	
N6	0.59282 (14)	0.4667 (3)	0.8058 (3)	0.0539 (10)	
C1	0.5358 (2)	0.7678 (4)	0.7590 (4)	0.0609 (13)	
H1	0.565706	0.780895	0.745405	0.073*	
C2	0.500000	0.8126 (5)	0.7202 (5)	0.0587 (18)	
C3	0.500000	0.8891 (6)	0.6506 (6)	0.079 (3)	
H3A	0.518668	0.872145	0.598226	0.118*	0.5
H3B	0.511448	0.945162	0.679167	0.118*	0.5
H3C	0.469884	0.899900	0.628993	0.118*	0.5
C4	0.62233 (19)	0.7576 (4)	0.9404 (5)	0.0657 (14)	
H4	0.628685	0.774661	0.878628	0.079*	
C5	0.6437 (2)	0.7921 (4)	1.0187 (5)	0.0703 (16)	
C6	0.62351 (18)	0.7461 (4)	1.0898 (5)	0.0652 (14)	
H6	0.630845	0.753535	1.152979	0.078*	
C7	0.6816 (3)	0.8593 (5)	1.0213 (7)	0.101 (3)	
H7A	0.683008	0.892650	0.962700	0.151*	
H7B	0.709188	0.825921	1.030975	0.151*	
H7C	0.677125	0.902956	1.071780	0.151*	
C8	0.5352 (2)	0.7387 (4)	1.2703 (4)	0.0666 (15)	
H8	0.565145	0.752606	1.283057	0.080*	
C9	0.500000	0.7775 (5)	1.3156 (5)	0.064 (2)	
C10	0.500000	0.8480 (6)	1.3918 (6)	0.086 (3)	
H10A	0.490217	0.907187	1.367269	0.129*	0.5
H10B	0.529821	0.854159	1.416715	0.129*	0.5
H10C	0.479962	0.828269	1.440855	0.129*	0.5
C11	0.6266 (2)	0.5890 (4)	0.7610 (4)	0.0692 (16)	
H11	0.634535	0.651797	0.754985	0.083*	
C12	0.6477 (2)	0.5164 (5)	0.7165 (5)	0.0735 (17)	
C13	0.6252 (2)	0.4411 (4)	0.7477 (4)	0.0703 (16)	
H13	0.631512	0.379548	0.730789	0.084*	
C14	0.6864 (3)	0.5184 (7)	0.6512 (7)	0.121 (4)	
H14A	0.710561	0.482164	0.677086	0.181*	
H14B	0.696028	0.581884	0.642415	0.181*	
H14C	0.677558	0.492285	0.591770	0.181*	
N13	0.4815 (5)	0.000000	1.000000	0.100 (4)	0.5
C15	0.4537 (5)	−0.0397 (11)	0.9222 (11)	0.090 (3)	0.5
H15A	0.473817	−0.075332	0.882295	0.109*	0.5
H15B	0.433185	−0.084241	0.950553	0.109*	0.5
C16	0.4280 (7)	0.0177 (15)	0.8628 (14)	0.089 (4)	0.5
H16A	0.448658	0.055337	0.825940	0.107*	0.5
H16B	0.411012	0.060362	0.902100	0.107*	0.5
C17	0.3975 (6)	−0.0255 (11)	0.7995 (13)	0.094 (4)	0.5
H17A	0.377538	−0.065837	0.835037	0.113*	0.5
H17B	0.414346	−0.064764	0.756454	0.113*	0.5
C18	0.3708 (8)	0.0388 (14)	0.7456 (16)	0.125 (6)	0.5
H18A	0.389807	0.072315	0.702648	0.188*	0.5
H18B	0.356366	0.082298	0.787231	0.188*	0.5
H18C	0.348496	0.004734	0.710954	0.188*	0.5
C19	0.5107 (5)	0.0733 (13)	0.9596 (11)	0.120 (5)	0.5
H19A	0.492025	0.127130	0.945372	0.144*	0.5
H19B	0.531809	0.092416	1.007810	0.144*	0.5
C20	0.5356 (6)	0.0507 (19)	0.8774 (13)	0.137 (5)	0.5
H20A	0.518928	0.001007	0.846555	0.165*	0.5
H20B	0.533222	0.104983	0.836720	0.165*	0.5
C21	0.5809 (8)	0.023 (3)	0.8740 (17)	0.135 (6)	0.5
H21A	0.583240	−0.041386	0.894383	0.162*	0.5
H21B	0.597919	0.061308	0.917907	0.162*	0.5
C22	0.6008 (7)	0.0319 (18)	0.7816 (13)	0.124 (6)	0.5
H22A	0.579926	0.010047	0.735044	0.186*	0.5
H22B	0.627737	−0.004991	0.778724	0.186*	0.5
H22C	0.607966	0.096364	0.769627	0.186*	0.5
N14	0.4764 (7)	0.500000	0.500000	0.126 (5)	0.5
C23	0.4479 (6)	0.5758 (13)	0.4587 (14)	0.119 (4)	0.5
H23A	0.466686	0.610878	0.415569	0.143*	0.5
H23B	0.424930	0.545448	0.420988	0.143*	0.5
C24	0.4258 (8)	0.6419 (16)	0.5169 (16)	0.126 (5)	0.5
H24A	0.448155	0.683712	0.543148	0.151*	0.5
H24B	0.411954	0.608600	0.568878	0.151*	0.5
C25	0.3920 (8)	0.6972 (18)	0.4715 (15)	0.128 (6)	0.5
H25	0.387774	0.696225	0.406695	0.154*	0.5
C26	0.3652 (9)	0.7541 (18)	0.5326 (17)	0.151 (7)	0.5
H26A	0.353377	0.806238	0.497887	0.226*	0.5
H26B	0.383368	0.776639	0.583504	0.226*	0.5
H26C	0.340833	0.717454	0.557193	0.226*	0.5
C27	0.5046 (9)	0.4566 (12)	0.4254 (13)	0.139 (5)	0.5
H27A	0.484816	0.434219	0.376207	0.167*	0.5
H27B	0.523235	0.505332	0.398239	0.167*	0.5
C28	0.5333 (6)	0.3812 (14)	0.452 (2)	0.153 (5)	0.5
H28A	0.521661	0.359587	0.512091	0.184*	0.5
H28B	0.526958	0.331581	0.407474	0.184*	0.5
C29	0.5802 (8)	0.3813 (16)	0.463 (3)	0.153 (6)	0.5
H29A	0.587533	0.418759	0.517371	0.184*	0.5
H29B	0.593039	0.412736	0.408333	0.184*	0.5
C30	0.6023 (9)	0.2919 (14)	0.472 (2)	0.145 (8)	0.5
H30A	0.618222	0.277503	0.415558	0.217*	0.5
H30B	0.580198	0.244295	0.484161	0.217*	0.5
H30C	0.622993	0.294464	0.524036	0.217*	0.5
Cl1	0.7285 (4)	0.6122 (8)	1.0070 (9)	0.138 (4)	0.5
Cl2	0.7325 (2)	0.3853 (3)	0.9903 (3)	0.0567 (10)	0.5
C31	0.6877 (5)	0.5509 (15)	0.9703 (18)	0.106 (5)	0.5
H31A	0.660627	0.579996	0.994674	0.127*	0.5
H31B	0.686838	0.558738	0.902605	0.127*	0.5
C32	0.6835 (5)	0.4558 (14)	0.9872 (16)	0.096 (5)	0.5
H32A	0.663778	0.430247	0.939307	0.115*	0.5
H32B	0.668218	0.448365	1.047057	0.115*	0.5
Cl3	0.4068 (3)	0.9999 (18)	0.4979 (15)	0.141 (3)	0.369 (6)
Cl4	0.2736 (2)	0.9972 (15)	0.5343 (9)	0.168 (6)	0.369 (6)
C33	0.3578 (7)	0.962 (2)	0.499 (3)	0.174 (8)	0.369 (6)
H33A	0.357513	0.905275	0.461575	0.209*	0.369 (6)
H33B	0.350960	0.944358	0.562913	0.209*	0.369 (6)
C34	0.3224 (8)	1.018 (3)	0.467 (3)	0.178 (8)	0.369 (6)
H34A	0.316359	1.004016	0.401455	0.214*	0.369 (6)
H34B	0.330723	1.083400	0.471696	0.214*	0.369 (6)
Cl5	0.3010 (7)	0.8437 (14)	0.7485 (15)	0.220 (9)	0.430 (5)
Cl6	0.1916 (5)	0.8177 (11)	0.7806 (12)	0.191 (7)	0.430 (5)
C35	0.2663 (9)	0.789 (2)	0.6864 (19)	0.189 (8)	0.430 (5)
H35A	0.253110	0.834059	0.643004	0.226*	0.430 (5)
H35B	0.282908	0.744430	0.649016	0.226*	0.430 (5)
C36	0.2312 (8)	0.7416 (16)	0.730 (2)	0.177 (8)	0.430 (5)
H36A	0.243427	0.701578	0.779210	0.213*	0.430 (5)
H36B	0.216299	0.701941	0.684716	0.213*	0.430 (5)
Cl7	0.2016 (4)	0.2257 (11)	0.8159 (9)	0.131 (4)	0.5
Cl8	0.3112 (5)	0.2379 (11)	0.6531 (10)	0.148 (4)	0.5
C37	0.2346 (6)	0.2772 (15)	0.7457 (19)	0.144 (6)	0.5
H37A	0.218165	0.304617	0.693555	0.172*	0.5
H37B	0.251215	0.326180	0.777619	0.172*	0.5
C38	0.2643 (8)	0.2042 (17)	0.7133 (19)	0.158 (6)	0.5
H38A	0.247185	0.162423	0.673214	0.190*	0.5
H38B	0.273892	0.168232	0.767563	0.190*	0.5

### Bis(tetrabutylazanium) hexa-µ4-hydroxido-dodeca-µ2-(4-methylpyrazolato)-hexahedro-octanickel 1,2-dichloroethane 7.196-solvate (2) Atomic displacement parameters (Å^2^)

**Table:**

	*U*^11^	*U*^22^	*U*^33^	*U*^12^	*U*^13^	*U*^23^
Ni1	0.0413 (4)	0.0365 (4)	0.0398 (4)	−0.0013 (3)	0.0037 (3)	0.0032 (3)
Ni2	0.0425 (4)	0.0364 (4)	0.0396 (4)	−0.0022 (3)	−0.0036 (3)	−0.0042 (3)
O1	0.039 (2)	0.0316 (19)	0.039 (2)	0.000	0.000	−0.0004 (15)
O2	0.0345 (19)	0.0354 (19)	0.040 (2)	0.000	0.000	0.0006 (15)
O3	0.046 (2)	0.038 (2)	0.0300 (18)	0.000	0.000	−0.0005 (15)
N1	0.057 (2)	0.045 (2)	0.045 (2)	−0.0017 (17)	0.0026 (16)	0.0108 (16)
N2	0.046 (2)	0.046 (2)	0.069 (3)	−0.0084 (17)	0.0004 (19)	0.0035 (19)
N3	0.048 (2)	0.042 (2)	0.060 (2)	−0.0068 (16)	−0.0039 (18)	−0.0075 (17)
N4	0.061 (2)	0.045 (2)	0.048 (2)	−0.0003 (17)	−0.0066 (17)	−0.0133 (17)
N5	0.055 (2)	0.052 (2)	0.049 (2)	0.0013 (18)	0.0140 (17)	0.0082 (18)
N6	0.051 (2)	0.056 (2)	0.055 (2)	0.0022 (18)	0.0143 (18)	−0.0051 (19)
C1	0.071 (3)	0.056 (3)	0.056 (3)	−0.006 (2)	0.005 (2)	0.019 (2)
C2	0.087 (5)	0.046 (4)	0.043 (3)	0.000	0.000	0.004 (3)
C3	0.130 (8)	0.056 (5)	0.051 (4)	0.000	0.000	0.012 (4)
C4	0.061 (3)	0.055 (3)	0.081 (4)	−0.016 (2)	0.005 (3)	0.012 (3)
C5	0.059 (3)	0.049 (3)	0.102 (5)	−0.015 (3)	−0.006 (3)	−0.001 (3)
C6	0.058 (3)	0.054 (3)	0.084 (4)	−0.014 (2)	−0.009 (3)	−0.015 (3)
C7	0.077 (5)	0.066 (4)	0.158 (8)	−0.032 (4)	−0.014 (5)	0.013 (5)
C8	0.080 (4)	0.059 (3)	0.061 (3)	−0.003 (3)	−0.012 (3)	−0.027 (3)
C9	0.096 (6)	0.045 (4)	0.050 (4)	0.000	0.000	−0.008 (3)
C10	0.146 (10)	0.060 (5)	0.052 (5)	0.000	0.000	−0.019 (4)
C11	0.066 (3)	0.067 (3)	0.074 (4)	−0.001 (3)	0.028 (3)	0.016 (3)
C12	0.069 (4)	0.081 (4)	0.071 (4)	0.005 (3)	0.029 (3)	0.004 (3)
C13	0.073 (4)	0.067 (3)	0.071 (3)	0.005 (3)	0.030 (3)	−0.007 (3)
C14	0.102 (6)	0.135 (8)	0.125 (7)	0.010 (5)	0.073 (6)	0.022 (6)
N13	0.080 (7)	0.122 (9)	0.097 (8)	0.000	0.000	−0.033 (7)
C15	0.079 (6)	0.097 (7)	0.095 (7)	0.005 (6)	−0.006 (5)	−0.033 (6)
C16	0.089 (8)	0.082 (7)	0.097 (8)	0.002 (6)	−0.019 (7)	−0.031 (6)
C17	0.104 (9)	0.079 (7)	0.099 (9)	0.003 (7)	−0.018 (8)	−0.031 (7)
C18	0.137 (14)	0.107 (12)	0.133 (14)	0.004 (11)	−0.027 (12)	−0.017 (11)
C19	0.092 (9)	0.164 (10)	0.105 (8)	−0.006 (7)	0.004 (6)	−0.028 (8)
C20	0.112 (9)	0.187 (11)	0.112 (9)	−0.017 (9)	0.009 (8)	−0.026 (9)
C21	0.111 (11)	0.188 (13)	0.107 (10)	−0.016 (10)	0.023 (9)	−0.027 (10)
C22	0.107 (12)	0.177 (16)	0.088 (11)	−0.046 (13)	0.033 (10)	−0.019 (13)
N14	0.122 (10)	0.104 (9)	0.153 (11)	0.000	0.000	0.036 (8)
C23	0.127 (10)	0.104 (9)	0.125 (10)	0.015 (7)	0.012 (7)	0.044 (8)
C24	0.146 (11)	0.114 (10)	0.117 (10)	0.028 (8)	0.008 (8)	0.048 (8)
C25	0.157 (12)	0.118 (11)	0.109 (10)	0.033 (10)	0.007 (10)	0.043 (9)
C26	0.180 (17)	0.135 (15)	0.138 (15)	0.044 (14)	0.005 (14)	0.005 (13)
C27	0.129 (10)	0.106 (8)	0.184 (11)	0.006 (8)	0.013 (9)	0.035 (8)
C28	0.138 (10)	0.111 (9)	0.209 (13)	0.017 (9)	0.029 (10)	0.030 (9)
C29	0.139 (11)	0.105 (10)	0.216 (14)	0.030 (10)	0.046 (12)	0.039 (11)
C30	0.144 (15)	0.074 (10)	0.216 (19)	0.033 (11)	0.063 (15)	0.021 (14)
Cl1	0.063 (4)	0.123 (7)	0.230 (10)	−0.008 (4)	0.002 (4)	−0.014 (6)
Cl2	0.040 (2)	0.051 (2)	0.079 (2)	0.0002 (15)	−0.0026 (15)	−0.0017 (17)
C31	0.058 (6)	0.091 (7)	0.169 (14)	0.011 (8)	−0.018 (8)	−0.003 (10)
C32	0.051 (6)	0.075 (6)	0.162 (13)	0.007 (6)	−0.026 (7)	−0.001 (10)
Cl3	0.166 (7)	0.133 (5)	0.123 (5)	0.028 (11)	0.057 (9)	0.001 (4)
Cl4	0.082 (4)	0.158 (7)	0.263 (19)	0.004 (5)	0.008 (6)	0.004 (14)
C33	0.159 (14)	0.154 (14)	0.208 (16)	0.022 (12)	0.017 (14)	0.020 (14)
C34	0.150 (14)	0.162 (14)	0.223 (17)	0.013 (12)	0.010 (13)	0.026 (15)
Cl5	0.223 (15)	0.230 (15)	0.205 (13)	−0.046 (11)	−0.042 (11)	0.016 (10)
Cl6	0.150 (7)	0.205 (12)	0.217 (13)	−0.052 (8)	−0.034 (8)	−0.106 (10)
C35	0.166 (14)	0.205 (16)	0.196 (16)	−0.040 (13)	−0.033 (13)	−0.059 (13)
C36	0.147 (13)	0.200 (16)	0.185 (15)	−0.034 (12)	−0.024 (13)	−0.076 (13)
Cl7	0.100 (5)	0.166 (9)	0.128 (7)	−0.008 (5)	−0.007 (4)	0.052 (6)
Cl8	0.157 (10)	0.128 (5)	0.158 (10)	−0.024 (6)	−0.034 (6)	−0.031 (6)
C37	0.139 (13)	0.146 (12)	0.146 (12)	−0.025 (9)	−0.009 (12)	0.015 (11)
C38	0.150 (13)	0.162 (12)	0.163 (13)	−0.033 (10)	−0.009 (11)	−0.002 (11)

### Bis(tetrabutylazanium) hexa-µ4-hydroxido-dodeca-µ2-(4-methylpyrazolato)-hexahedro-octanickel 1,2-dichloroethane 7.196-solvate (2) Geometric parameters (Å, º)

**Table:**

Ni1—N2	2.019 (4)	C17—C18	1.464 (19)
Ni1—N1	2.028 (4)	C17—H17A	0.9900
Ni1—N5	2.031 (4)	C17—H17B	0.9900
Ni1—O2	2.1735 (12)	C18—H18A	0.9800
Ni1—O1	2.175 (3)	C18—H18B	0.9800
Ni1—O3^i^	2.181 (3)	C18—H18C	0.9800
Ni1—Ni1^ii^	2.9859 (14)	C19—C20	1.451 (15)
Ni1—Ni2	2.9939 (10)	C19—H19A	0.9900
Ni1—Ni2^iii^	2.9988 (10)	C19—H19B	0.9900
Ni2—N4	2.023 (4)	C20—C21	1.436 (17)
Ni2—N6^iii^	2.025 (4)	C20—H20A	0.9900
Ni2—N3	2.027 (4)	C20—H20B	0.9900
Ni2—O3	2.175 (3)	C21—C22	1.48 (2)
Ni2—O2	2.1766 (12)	C21—H21A	0.9900
Ni2—O1	2.178 (3)	C21—H21B	0.9900
Ni2—Ni2^ii^	2.9835 (14)	C22—H22A	0.9800
O1—H1O	0.85 (2)	C22—H22B	0.9800
O2—H2O	0.84 (2)	C22—H22C	0.9800
O3—H3O	0.85 (2)	N14—C27	1.519 (13)
N1—N1^ii^	1.337 (8)	N14—C23	1.527 (13)
N1—C1	1.339 (6)	C23—C24	1.447 (17)
N2—N3	1.341 (6)	C23—H23A	0.9900
N2—C4	1.350 (6)	C23—H23B	0.9900
N3—C6	1.346 (6)	C24—C25	1.462 (17)
N4—N4^ii^	1.329 (8)	C24—H24A	0.9900
N4—C8	1.341 (6)	C24—H24B	0.9900
N5—N6	1.333 (6)	C25—C26	1.46 (2)
N5—C11	1.336 (6)	C25—H25	0.9500
N6—C13	1.349 (6)	C26—H26A	0.9800
C1—C2	1.387 (7)	C26—H26B	0.9800
C1—H1	0.9500	C26—H26C	0.9800
C2—C3	1.506 (10)	C27—C28	1.454 (16)
C3—H3A	0.9800	C27—H27A	0.9900
C3—H3B	0.9800	C27—H27B	0.9900
C3—H3C	0.9800	C28—C29	1.436 (17)
C3—H3A^ii^	0.98 (8)	C28—H28A	0.9900
C3—H3B^ii^	0.98 (6)	C28—H28B	0.9900
C3—H3C^ii^	0.98 (2)	C29—C30	1.472 (19)
C4—C5	1.402 (9)	C29—H29A	0.9900
C4—H4	0.9500	C29—H29B	0.9900
C5—C6	1.375 (9)	C30—H30A	0.9800
C5—C7	1.512 (8)	C30—H30B	0.9800
C6—H6	0.9500	C30—H30C	0.9800
C7—H7A	0.9800	Cl1—C31	1.619 (18)
C7—H7B	0.9800	Cl2—C32	1.811 (15)
C7—H7C	0.9800	C31—C32	1.414 (13)
C8—C9	1.377 (8)	C31—H31A	0.9900
C8—H8	0.9500	C31—H31B	0.9900
C9—C10	1.509 (10)	C32—H32A	0.9900
C10—H10A	0.9800	C32—H32B	0.9900
C10—H10B	0.9800	Cl3—C33	1.59 (2)
C10—H10C	0.9800	Cl4—C34	1.801 (19)
C10—H10A^ii^	0.98 (6)	C33—C34	1.42 (2)
C10—H10B^ii^	0.98 (4)	C33—H33A	0.9900
C10—H10C^ii^	0.98 (10)	C33—H33B	0.9900
C11—C12	1.397 (9)	C34—H34A	0.9900
C11—H11	0.9500	C34—H34B	0.9900
C12—C13	1.370 (9)	Cl5—C35	1.60 (2)
C12—C14	1.508 (9)	Cl6—C36	1.791 (19)
C13—H13	0.9500	C35—C36	1.43 (2)
C14—H14A	0.9800	C35—H35A	0.9900
C14—H14B	0.9800	C35—H35B	0.9900
C14—H14C	0.9800	C36—H36A	0.9900
N13—N13^iv^	1.12 (3)	C36—H36B	0.9900
N13—C19	1.507 (13)	Cl7—C37	1.615 (19)
N13—C15	1.524 (12)	Cl8—C38	1.741 (17)
C15—C16	1.432 (16)	C37—C38	1.474 (19)
C15—H15A	0.9900	C37—H37A	0.9900
C15—H15B	0.9900	C37—H37B	0.9900
C16—C17	1.450 (16)	C38—H38A	0.9900
C16—H16A	0.9900	C38—H38B	0.9900
C16—H16B	0.9900		
			
N2—Ni1—N1	96.13 (17)	H10A—C10—H10A^ii^	35.3
N2—Ni1—N5	95.70 (18)	H10B—C10—H10A^ii^	76.8
N1—Ni1—N5	97.14 (16)	H10C—C10—H10A^ii^	135.3
N2—Ni1—O2	91.76 (14)	C9—C10—H10B^ii^	109.5 (8)
N1—Ni1—O2	169.03 (16)	H10A—C10—H10B^ii^	76.8
N5—Ni1—O2	89.63 (14)	H10B—C10—H10B^ii^	135.3
N2—Ni1—O1	89.99 (15)	H10C—C10—H10B^ii^	35.3
N1—Ni1—O1	91.17 (15)	H10A^ii^—C10—H10B^ii^	109.5
N5—Ni1—O1	169.36 (16)	C9—C10—H10C^ii^	109 (2)
O2—Ni1—O1	81.20 (13)	H10A—C10—H10C^ii^	135.3
N2—Ni1—O3^i^	169.78 (17)	H10B—C10—H10C^ii^	35.3
N1—Ni1—O3^i^	90.20 (15)	H10C—C10—H10C^ii^	76.8
N5—Ni1—O3^i^	91.45 (15)	H10A^ii^—C10—H10C^ii^	109.5
O2—Ni1—O3^i^	80.97 (13)	H10B^ii^—C10—H10C^ii^	109.5
O1—Ni1—O3^i^	81.84 (12)	N5—C11—C12	110.4 (5)
N2—Ni1—Ni1^ii^	129.41 (12)	N5—C11—H11	124.8
N1—Ni1—Ni1^ii^	66.02 (11)	C12—C11—H11	124.8
N5—Ni1—Ni1^ii^	131.82 (13)	C13—C12—C11	102.9 (5)
O2—Ni1—Ni1^ii^	103.06 (11)	C13—C12—C14	127.7 (7)
O1—Ni1—Ni1^ii^	46.66 (7)	C11—C12—C14	129.3 (7)
O3^i^—Ni1—Ni1^ii^	46.80 (7)	N6—C13—C12	110.5 (5)
N2—Ni1—Ni2	66.05 (13)	N6—C13—H13	124.8
N1—Ni1—Ni2	131.00 (12)	C12—C13—H13	124.8
N5—Ni1—Ni2	128.46 (12)	C12—C14—H14A	109.5
O2—Ni1—Ni2	46.55 (3)	C12—C14—H14B	109.5
O1—Ni1—Ni2	46.58 (8)	H14A—C14—H14B	109.5
O3^i^—Ni1—Ni2	103.75 (11)	C12—C14—H14C	109.5
Ni1^ii^—Ni1—Ni2	89.976 (18)	H14A—C14—H14C	109.5
N2—Ni1—Ni2^iii^	131.15 (13)	H14B—C14—H14C	109.5
N1—Ni1—Ni2^iii^	129.39 (12)	N13^iv^—N13—C19	54.0 (9)
N5—Ni1—Ni2^iii^	65.70 (12)	N13^iv^—N13—C15	123.7 (8)
O2—Ni1—Ni2^iii^	46.46 (3)	C19—N13—C15	107.9 (9)
O1—Ni1—Ni2^iii^	103.83 (11)	C16—C15—N13	121.8 (13)
O3^i^—Ni1—Ni2^iii^	46.40 (8)	C16—C15—H15A	106.9
Ni1^ii^—Ni1—Ni2^iii^	89.975 (18)	N13—C15—H15A	106.9
Ni2—Ni1—Ni2^iii^	90.08 (3)	C16—C15—H15B	106.9
N4—Ni2—N6^iii^	96.94 (17)	N13—C15—H15B	106.9
N4—Ni2—N3	96.66 (17)	H15A—C15—H15B	106.7
N6^iii^—Ni2—N3	95.90 (18)	C15—C16—C17	118.5 (16)
N4—Ni2—O3	90.40 (15)	C15—C16—H16A	107.7
N6^iii^—Ni2—O3	91.01 (15)	C17—C16—H16A	107.7
N3—Ni2—O3	169.43 (16)	C15—C16—H16B	107.7
N4—Ni2—O2	168.93 (16)	C17—C16—H16B	107.7
N6^iii^—Ni2—O2	90.27 (14)	H16A—C16—H16B	107.1
N3—Ni2—O2	90.92 (14)	C16—C17—C18	114.5 (16)
O3—Ni2—O2	81.04 (13)	C16—C17—H17A	108.6
N4—Ni2—O1	90.80 (15)	C18—C17—H17A	108.6
N6^iii^—Ni2—O1	169.48 (17)	C16—C17—H17B	108.6
N3—Ni2—O1	90.24 (14)	C18—C17—H17B	108.6
O3—Ni2—O1	81.79 (12)	H17A—C17—H17B	107.6
O2—Ni2—O1	81.08 (13)	C17—C18—H18A	109.5
N4—Ni2—Ni2^ii^	65.86 (11)	C17—C18—H18B	109.5
N6^iii^—Ni2—Ni2^ii^	131.01 (13)	H18A—C18—H18B	109.5
N3—Ni2—Ni2^ii^	130.07 (12)	C17—C18—H18C	109.5
O3—Ni2—Ni2^ii^	46.69 (7)	H18A—C18—H18C	109.5
O2—Ni2—Ni2^ii^	103.07 (11)	H18B—C18—H18C	109.5
O1—Ni2—Ni2^ii^	46.76 (7)	C20—C19—N13	117.8 (16)
N4—Ni2—Ni1	130.48 (12)	C20—C19—H19A	107.9
N6^iii^—Ni2—Ni1	129.25 (13)	N13—C19—H19A	107.9
N3—Ni2—Ni1	65.71 (12)	C20—C19—H19B	107.9
O3—Ni2—Ni1	103.72 (11)	N13—C19—H19B	107.9
O2—Ni2—Ni1	46.47 (3)	H19A—C19—H19B	107.2
O1—Ni2—Ni1	46.51 (8)	C21—C20—C19	126.3 (18)
Ni2^ii^—Ni2—Ni1	90.024 (18)	C21—C20—H20A	105.7
N4—Ni2—Ni1^iii^	129.98 (13)	C19—C20—H20A	105.7
N6^iii^—Ni2—Ni1^iii^	65.79 (12)	C21—C20—H20B	105.7
N3—Ni2—Ni1^iii^	130.14 (12)	C19—C20—H20B	105.7
O3—Ni2—Ni1^iii^	46.57 (8)	H20A—C20—H20B	106.2
O2—Ni2—Ni1^iii^	46.38 (3)	C20—C21—C22	114 (2)
O1—Ni2—Ni1^iii^	103.75 (11)	C20—C21—H21A	108.9
Ni2^ii^—Ni2—Ni1^iii^	90.023 (18)	C22—C21—H21A	108.9
Ni1—Ni2—Ni1^iii^	89.93 (3)	C20—C21—H21B	108.9
Ni1^ii^—O1—Ni1	86.68 (15)	C22—C21—H21B	108.9
Ni1^ii^—O1—Ni2	152.4 (2)	H21A—C21—H21B	107.7
Ni1—O1—Ni2	86.91 (6)	C21—C22—H22A	109.5
Ni1^ii^—O1—Ni2^ii^	86.91 (6)	C21—C22—H22B	109.5
Ni1—O1—Ni2^ii^	152.4 (2)	H22A—C22—H22B	109.5
Ni2—O1—Ni2^ii^	86.47 (15)	C21—C22—H22C	109.5
Ni1^ii^—O1—H1O	112 (4)	H22A—C22—H22C	109.5
Ni1—O1—H1O	112 (4)	H22B—C22—H22C	109.5
Ni2—O1—H1O	95 (4)	C27—N14—C23	110.0 (10)
Ni2^ii^—O1—H1O	95 (4)	C24—C23—N14	121.0 (15)
Ni1—O2—Ni1^iii^	153.9 (2)	C24—C23—H23A	107.1
Ni1—O2—Ni2^iii^	87.16 (5)	N14—C23—H23A	107.1
Ni1^iii^—O2—Ni2^iii^	86.98 (5)	C24—C23—H23B	107.1
Ni1—O2—Ni2	86.98 (5)	N14—C23—H23B	107.1
Ni1^iii^—O2—Ni2	87.16 (5)	H23A—C23—H23B	106.8
Ni2^iii^—O2—Ni2	153.9 (2)	C23—C24—C25	115.4 (19)
Ni1—O2—H2O	103.06 (11)	C23—C24—H24A	108.4
Ni1^iii^—O2—H2O	103.06 (11)	C25—C24—H24A	108.4
Ni2^iii^—O2—H2O	103.07 (11)	C23—C24—H24B	108.4
Ni2—O2—H2O	103.07 (11)	C25—C24—H24B	108.4
Ni2—O3—Ni2^ii^	86.62 (15)	H24A—C24—H24B	107.5
Ni2—O3—Ni1^i^	152.5 (2)	C26—C25—C24	115.6 (19)
Ni2^ii^—O3—Ni1^i^	87.02 (5)	C26—C25—H25	122.2
Ni2—O3—Ni1^iii^	87.02 (5)	C24—C25—H25	122.2
Ni2^ii^—O3—Ni1^iii^	152.5 (2)	C25—C26—H26A	109.5
Ni1^i^—O3—Ni1^iii^	86.41 (15)	C25—C26—H26B	109.5
Ni2—O3—H3O	108 (4)	H26A—C26—H26B	109.5
Ni2^ii^—O3—H3O	108 (4)	C25—C26—H26C	109.5
Ni1^i^—O3—H3O	100 (4)	H26A—C26—H26C	109.5
Ni1^iii^—O3—H3O	100 (4)	H26B—C26—H26C	109.5
N1^ii^—N1—C1	108.2 (3)	C28—C27—N14	117.6 (17)
N1^ii^—N1—Ni1	113.98 (11)	C28—C27—H27A	107.9
C1—N1—Ni1	137.8 (4)	N14—C27—H27A	107.9
N3—N2—C4	108.6 (4)	C28—C27—H27B	107.9
N3—N2—Ni1	114.0 (3)	N14—C27—H27B	107.9
C4—N2—Ni1	137.2 (4)	H27A—C27—H27B	107.2
N2—N3—C6	107.5 (4)	C29—C28—C27	128 (2)
N2—N3—Ni2	114.2 (3)	C29—C28—H28A	105.2
C6—N3—Ni2	138.3 (4)	C27—C28—H28A	105.2
N4^ii^—N4—C8	107.6 (3)	C29—C28—H28B	105.2
N4^ii^—N4—Ni2	114.14 (11)	C27—C28—H28B	105.2
C8—N4—Ni2	138.2 (4)	H28A—C28—H28B	105.9
N6—N5—C11	107.9 (4)	C28—C29—C30	117 (2)
N6—N5—Ni1	114.1 (3)	C28—C29—H29A	107.9
C11—N5—Ni1	137.9 (4)	C30—C29—H29A	107.9
N5—N6—C13	108.2 (4)	C28—C29—H29B	107.9
N5—N6—Ni2^iii^	114.4 (3)	C30—C29—H29B	107.9
C13—N6—Ni2^iii^	137.1 (4)	H29A—C29—H29B	107.2
N1—C1—C2	110.1 (5)	C29—C30—H30A	109.5
N1—C1—H1	124.9	C29—C30—H30B	109.5
C2—C1—H1	124.9	H30A—C30—H30B	109.5
C1—C2—C1^ii^	103.3 (6)	C29—C30—H30C	109.5
C1—C2—C3	128.4 (3)	H30A—C30—H30C	109.5
C1^ii^—C2—C3	128.4 (3)	H30B—C30—H30C	109.5
C2—C3—H3A	109.5	C32—C31—Cl1	123.6 (15)
C2—C3—H3B	109.5	C32—C31—H31A	106.4
H3A—C3—H3B	109.5	Cl1—C31—H31A	106.4
C2—C3—H3C	109.5	C32—C31—H31B	106.4
H3A—C3—H3C	109.5	Cl1—C31—H31B	106.4
H3B—C3—H3C	109.5	H31A—C31—H31B	106.5
C2—C3—H3A^ii^	109.5 (17)	C31—C32—Cl2	119.1 (15)
H3A—C3—H3A^ii^	70.8	C31—C32—H32A	107.5
H3B—C3—H3A^ii^	138.1	Cl2—C32—H32A	107.5
H3C—C3—H3A^ii^	41.6	C31—C32—H32B	107.5
C2—C3—H3B^ii^	109.5 (13)	Cl2—C32—H32B	107.5
H3A—C3—H3B^ii^	138.1	H32A—C32—H32B	107.0
H3B—C3—H3B^ii^	41.6	C34—C33—Cl3	121 (2)
H3C—C3—H3B^ii^	70.8	C34—C33—H33A	107.2
H3A^ii^—C3—H3B^ii^	109.5	Cl3—C33—H33A	107.2
C2—C3—H3C^ii^	109.5 (5)	C34—C33—H33B	107.2
H3A—C3—H3C^ii^	41.6	Cl3—C33—H33B	107.2
H3B—C3—H3C^ii^	70.7	H33A—C33—H33B	106.8
H3C—C3—H3C^ii^	138.1	C33—C34—Cl4	110.6 (18)
H3A^ii^—C3—H3C^ii^	109.5	C33—C34—H34A	109.5
H3B^ii^—C3—H3C^ii^	109.5	Cl4—C34—H34A	109.5
N2—C4—C5	109.5 (5)	C33—C34—H34B	109.5
N2—C4—H4	125.2	Cl4—C34—H34B	109.5
C5—C4—H4	125.2	H34A—C34—H34B	108.1
C6—C5—C4	103.1 (5)	C36—C35—Cl5	119 (2)
C6—C5—C7	129.6 (7)	C36—C35—H35A	107.6
C4—C5—C7	127.2 (7)	Cl5—C35—H35A	107.6
N3—C6—C5	111.3 (6)	C36—C35—H35B	107.6
N3—C6—H6	124.3	Cl5—C35—H35B	107.6
C5—C6—H6	124.3	H35A—C35—H35B	107.0
C5—C7—H7A	109.5	C35—C36—Cl6	112.5 (19)
C5—C7—H7B	109.5	C35—C36—H36A	109.1
H7A—C7—H7B	109.5	Cl6—C36—H36A	109.1
C5—C7—H7C	109.5	C35—C36—H36B	109.1
H7A—C7—H7C	109.5	Cl6—C36—H36B	109.1
H7B—C7—H7C	109.5	H36A—C36—H36B	107.8
N4—C8—C9	111.4 (5)	C38—C37—Cl7	104.2 (14)
N4—C8—H8	124.3	C38—C37—H37A	110.9
C9—C8—H8	124.3	Cl7—C37—H37A	110.9
C8—C9—C8^ii^	102.0 (7)	C38—C37—H37B	110.9
C8—C9—C10	129.0 (3)	Cl7—C37—H37B	110.9
C8^ii^—C9—C10	129.0 (3)	H37A—C37—H37B	108.9
C9—C10—H10A	109.5	C37—C38—Cl8	117.3 (15)
C9—C10—H10B	109.5	C37—C38—H38A	108.0
H10A—C10—H10B	109.5	Cl8—C38—H38A	108.0
C9—C10—H10C	109.5	C37—C38—H38B	108.0
H10A—C10—H10C	109.5	Cl8—C38—H38B	108.0
H10B—C10—H10C	109.5	H38A—C38—H38B	107.2
C9—C10—H10A^ii^	109.5 (13)		
			
C4—N2—N3—C6	0.1 (6)	Ni1—N5—C11—C12	175.9 (5)
Ni1—N2—N3—C6	175.7 (3)	N5—C11—C12—C13	−0.9 (8)
C4—N2—N3—Ni2	−177.6 (4)	N5—C11—C12—C14	−179.9 (8)
Ni1—N2—N3—Ni2	−2.0 (4)	N5—N6—C13—C12	0.0 (7)
C11—N5—N6—C13	−0.5 (6)	Ni2^iii^—N6—C13—C12	−172.7 (5)
Ni1—N5—N6—C13	−176.9 (4)	C11—C12—C13—N6	0.5 (8)
C11—N5—N6—Ni2^iii^	174.0 (4)	C14—C12—C13—N6	179.6 (8)
Ni1—N5—N6—Ni2^iii^	−2.3 (4)	N13^iv^—N13—C15—C16	−110.9 (16)
N1^ii^—N1—C1—C2	1.2 (6)	C19—N13—C15—C16	−53 (2)
Ni1—N1—C1—C2	−179.3 (4)	N13—C15—C16—C17	−171.0 (18)
N1—C1—C2—C1^ii^	−1.8 (9)	C15—C16—C17—C18	176 (2)
N1—C1—C2—C3	180.0 (7)	N13^iv^—N13—C19—C20	69.8 (16)
N3—N2—C4—C5	−0.6 (6)	C15—N13—C19—C20	−49 (2)
Ni1—N2—C4—C5	−174.7 (4)	N13—C19—C20—C21	−98 (3)
N2—C4—C5—C6	0.9 (7)	C19—C20—C21—C22	−162 (3)
N2—C4—C5—C7	177.6 (7)	C27—N14—C23—C24	−165 (2)
N2—N3—C6—C5	0.5 (6)	N14—C23—C24—C25	−167 (2)
Ni2—N3—C6—C5	177.3 (4)	C23—C24—C25—C26	171 (2)
C4—C5—C6—N3	−0.8 (7)	C23—N14—C27—C28	−179 (2)
C7—C5—C6—N3	−177.4 (7)	N14—C27—C28—C29	−107 (4)
N4^ii^—N4—C8—C9	1.6 (6)	C27—C28—C29—C30	−168 (3)
Ni2—N4—C8—C9	178.7 (5)	Cl1—C31—C32—Cl2	−35 (3)
N4—C8—C9—C8^ii^	−2.4 (9)	Cl3—C33—C34—Cl4	−144 (3)
N4—C8—C9—C10	−179.6 (8)	Cl5—C35—C36—Cl6	70 (3)
N6—N5—C11—C12	0.9 (7)	Cl7—C37—C38—Cl8	−168 (2)

Symmetry codes: (i) −*x*+1, −*y*+1, −*z*+2; (ii) −*x*+1, *y*, *z*; (iii) *x*, −*y*+1, −*z*+2; (iv) −*x*+1, −*y*, −*z*+2.

### Bis(tetrabutylazanium) hexa-µ4-hydroxido-dodeca-µ2-(4-octylpyrazolato)-hexahedro-octanickel (3) Crystal data

**Table:**

(C_16_H_36_N)_2_[Ni_8_(C_11_H_19_N_2_)_12_(OH)_6_]	*Z* = 2
*M**_r_* = 3208.01	*F*(000) = 3488
Triclinic, *P*1	*D*_x_ = 1.192 Mg m^−^^3^
*a* = 13.973 (8) Å	Cu *K*α radiation, λ = 1.54178 Å
*b* = 20.765 (10) Å	Cell parameters from 9844 reflections
*c* = 31.873 (14) Å	θ = 3.9–70.0°
α = 99.896 (17)°	µ = 1.33 mm^−^^1^
β = 94.798 (17)°	*T* = 150 K
γ = 99.10 (2)°	Flat needle, light blue
*V* = 8938 (8) Å^3^	0.22 × 0.08 × 0.03 mm

### Bis(tetrabutylazanium) hexa-µ4-hydroxido-dodeca-µ2-(4-octylpyrazolato)-hexahedro-octanickel (3) Data collection

**Table:**

Bruker AXS D8 Quest diffractometer with PhotonIII_C14 charge-integrating and photon counting pixel array detector	52094 independent reflections
Radiation source: I-mu-S microsource X-ray tube	37936 reflections with *I* > 2σ(*I*)
Laterally graded multilayer (Goebel) mirror monochromator	*R*_int_ = 0.071
Detector resolution: 7.4074 pixels mm^-1^	θ_max_ = 70.6°, θ_min_ = 2.4°
ω and phi scans	*h* = −16→16
Absorption correction: multi-scan (*TWINABS*; Sheldrick, 2012)	*k* = −25→25
*T*_min_ = 0.594, *T*_max_ = 0.753	*l* = 0→38
110848 measured reflections	

### Bis(tetrabutylazanium) hexa-µ4-hydroxido-dodeca-µ2-(4-octylpyrazolato)-hexahedro-octanickel (3) Refinement

**Table:**

Refinement on *F*^2^	Primary atom site location: dual
Least-squares matrix: full	Secondary atom site location: difference Fourier map
*R*[*F*^2^ > 2σ(*F*^2^)] = 0.066	Hydrogen site location: mixed
*wR*(*F*^2^) = 0.185	H atoms treated by a mixture of independent and constrained refinement
*S* = 1.05	*w* = 1/[σ^2^(*F*_o_^2^) + (0.0741*P*)^2^ + 9.544*P*] where *P* = (*F*_o_^2^ + 2*F*_c_^2^)/3
52094 reflections	(Δ/σ)_max_ = 0.001
2507 parameters	Δρ_max_ = 0.73 e Å^−^^3^
5486 restraints	Δρ_min_ = −0.51 e Å^−^^3^

### Bis(tetrabutylazanium) hexa-µ4-hydroxido-dodeca-µ2-(4-octylpyrazolato)-hexahedro-octanickel (3) Special details

**Table:**

Geometry. All esds (except the esd in the dihedral angle between two l.s. planes) are estimated using the full covariance matrix. The cell esds are taken into account individually in the estimation of esds in distances, angles and torsion angles; correlations between esds in cell parameters are only used when they are defined by crystal symmetry. An approximate (isotropic) treatment of cell esds is used for estimating esds involving l.s. planes.
Refinement. The crystal under investigation was found to be non-merohedrally twinned. The orientation matrices for the two components were identified using the program Cell_Now, with the two components being related by a 180° rotation around the reciprocal *b*-axis. The two components were integrated using SAINT and corrected for absorption using TWINABS, resulting in the following statistics:27457 data (13708 unique) involve domain 1 only, mean I/σ 8.9 27508 data (13715 unique) involve domain 2 only, mean I/σ 8.1 57229 data (24936 unique) involve 2 domains, mean I/σ 11.4 1 data (1 unique) involve 3 domains, mean I/σ 2.7The exact twin matrix identified by the integration program was found to be: –0.99999 0.00004 –0.00000 0.51610 1.00001 0.24406 –0.00001 –0.00019 –1.00002The structure was solved using dual methods using all non-overlapping reflections of both components. The structure was refined using the HKLF 5 routine with all reflections of both components (including overlapping reflections), resulting in a BASF value of 0.4564 (7). The R_int_ value given is for all reflections and is based on agreement between observed single and composite intensities and those calculated from refined unique intensities and twin fractions (TWINABS (Sheldrick, 2012)).Some of the alkyl chains were refined as two-fold disordered. The geometries of all fully occupied and major moiety pyrazolate ligands were restrained to be similar to each other, and disordered major and minor moieties were restrained to have similar geometries. U_ij_ components of ADPs for disordered atoms closer to each other than 2.0 Å were restrained to be similar. Subject to these conditions the occupancy ratio refined to 0.713 (8)/0.287 (8) (C5–C11 residue 3), 0.507 (8)/0.493 (8) (C5–C11 residue 4), 0.518 (9)/0.482 (9) (C5–C11 residue 5), 0.450 (13)/0.550 (13) (C6–C11 residue 7), and 0.524 (11)/0.476 (11) (C5–C11 residue 10). Two Bu_4_N^+^ cations are disordered around inversion centers (residues 14 and 15). One of them is additionally disordered (residue 15). The geometries of all partially occupied Bu_4_N^+^ cations were restrained to be similar to that of the single non-disordered cation (residue 13). All N–C bond lengths in all cations were restrained to be similar to each other. U_ij_ components of ADPs for disordered atoms closer to each other than 2.0 Å were restrained to be similar. Subject to these conditions the occupancy ratio for the additionally split cation refined to two times 0.257 (5) and two times 0.243 (5). Hydroxyl H atom positions were refined and O–H distances were restrained to 0.84 (2) Å. All hydroxyl H–O–Ni angles were restrained (via the H···Ni distances) to be similar to each other.

### Bis(tetrabutylazanium) hexa-µ4-hydroxido-dodeca-µ2-(4-octylpyrazolato)-hexahedro-octanickel (3) Fractional atomic coordinates and isotropic or equivalent isotropic displacement parameters (Å^2^)

**Table:**

	*x*	*y*	*z*	*U*_iso_*/*U*_eq_	Occ. (<1)
Ni1_16	0.40937 (6)	0.59193 (4)	0.48006 (3)	0.03648 (19)	
Ni2_16	0.62348 (6)	0.59550 (4)	0.48257 (3)	0.03650 (19)	
Ni3_16	0.58936 (6)	0.45370 (4)	0.43611 (2)	0.03420 (18)	
Ni4_16	0.37488 (6)	0.45100 (4)	0.43373 (3)	0.03607 (19)	
Ni5_16	0.43942 (5)	0.59817 (3)	0.04516 (2)	0.02700 (16)	
Ni6_16	0.39490 (5)	0.45647 (3)	0.05544 (2)	0.02920 (17)	
Ni7_16	0.60458 (5)	0.44538 (3)	0.05024 (2)	0.02798 (17)	
Ni8_16	0.64844 (5)	0.58690 (3)	0.03919 (2)	0.02761 (16)	
O1_16	0.4992 (2)	0.53022 (14)	0.44333 (10)	0.0337 (7)	
H1_16	0.4985 (11)	0.5435 (7)	0.4204 (5)	0.051*	
O2_16	0.3559 (2)	0.49862 (15)	0.49881 (10)	0.0342 (7)	
H2_16	0.2962 (12)	0.4961 (7)	0.4975 (5)	0.051*	
O3_16	0.4755 (2)	0.40496 (14)	0.46917 (10)	0.0340 (7)	
H3_16	0.4664 (11)	0.3661 (8)	0.4562 (5)	0.051*	
O4_16	0.5298 (2)	0.52864 (13)	0.06389 (9)	0.0266 (6)	
H4_16	0.5409 (11)	0.5412 (7)	0.0902 (5)	0.040*	
O5_16	0.6415 (2)	0.49218 (13)	−0.00380 (9)	0.0278 (6)	
H5_16	0.6980 (12)	0.4885 (7)	−0.0056 (5)	0.042*	
O6_16	0.5290 (2)	0.59454 (13)	−0.00681 (9)	0.0271 (6)	
H6_16	0.5414 (11)	0.6339 (8)	−0.0100 (5)	0.041*	
N1_1	0.3355 (4)	0.6415 (2)	0.52270 (14)	0.0482 (11)	
N2_1	0.3333 (3)	0.6212 (2)	0.56053 (13)	0.0419 (10)	
C1_1	0.2992 (5)	0.6977 (3)	0.52614 (18)	0.0596 (17)	
H1_1	0.292173	0.721841	0.503659	0.072*	
C2_1	0.2733 (4)	0.7158 (3)	0.56662 (18)	0.0525 (14)	
C3_1	0.2959 (4)	0.6659 (3)	0.58680 (17)	0.0511 (14)	
H3_1	0.286378	0.663445	0.615681	0.061*	
C4_1	0.2344 (6)	0.7783 (3)	0.5840 (2)	0.075 (2)	
H4A_1	0.166334	0.765448	0.590001	0.091*	
H4B_1	0.273590	0.800325	0.611517	0.091*	
C5_1	0.2366 (6)	0.8268 (3)	0.5544 (3)	0.079 (2)	
H5A_1	0.195607	0.804672	0.527316	0.095*	
H5B_1	0.304323	0.837521	0.547431	0.095*	
C6_1	0.2025 (7)	0.8914 (3)	0.5703 (3)	0.089 (2)	
H6A_1	0.247361	0.916420	0.595545	0.106*	
H6B_1	0.136824	0.881369	0.579603	0.106*	
C7_1	0.1984 (8)	0.9344 (4)	0.5364 (3)	0.108 (3)	
H7A_1	0.263929	0.943314	0.526809	0.129*	
H7B_1	0.153073	0.909111	0.511370	0.129*	
C8_1	0.1664 (8)	0.9995 (4)	0.5507 (3)	0.123 (4)	
H8A_1	0.214428	1.026397	0.574277	0.147*	
H8B_1	0.102955	0.990948	0.562290	0.147*	
C9_1	0.1559 (10)	1.0387 (5)	0.5158 (4)	0.146 (5)	
H9A_1	0.110499	1.010961	0.491547	0.175*	
H9B_1	0.220144	1.049366	0.505249	0.175*	
C10_1	0.1183 (12)	1.1031 (5)	0.5298 (4)	0.179 (6)	
H10A_1	0.054818	1.093049	0.541089	0.214*	
H10B_1	0.164804	1.132058	0.553225	0.214*	
C11_1	0.1068 (13)	1.1374 (7)	0.4947 (6)	0.231 (9)	
H11A_1	0.069239	1.106484	0.469900	0.346*	
H11B_1	0.171097	1.154758	0.487125	0.346*	
H11C_1	0.072042	1.174188	0.503080	0.346*	
N1_2	0.5803 (3)	0.67681 (19)	0.46577 (14)	0.0473 (11)	
N2_2	0.4820 (3)	0.6745 (2)	0.46344 (14)	0.0469 (11)	
C1_2	0.6204 (5)	0.7344 (3)	0.4559 (2)	0.0618 (17)	
H1_2	0.688271	0.748048	0.455119	0.074*	
C2_2	0.5498 (5)	0.7712 (3)	0.4469 (2)	0.072 (2)	
C3_2	0.4640 (5)	0.7318 (3)	0.4520 (2)	0.0602 (17)	
H3_2	0.401017	0.743173	0.448101	0.072*	
C4_2	0.5667 (7)	0.8370 (3)	0.4327 (4)	0.119 (4)	
H4A_2	0.631893	0.861008	0.446148	0.143*	
H4B_2	0.569207	0.828283	0.401348	0.143*	
C5_2	0.4963 (5)	0.8818 (3)	0.4419 (2)	0.0641 (17)	
H5A_2	0.495333	0.892628	0.473367	0.077*	
H5B_2	0.430419	0.858018	0.429232	0.077*	
C6_2	0.5167 (7)	0.9455 (3)	0.4255 (3)	0.087 (3)	
H6A_2	0.585337	0.966026	0.435900	0.104*	
H6B_2	0.512606	0.933683	0.393833	0.104*	
C7_2	0.4567 (6)	0.9974 (3)	0.4356 (2)	0.077 (2)	
H7A_2	0.461310	1.010325	0.467136	0.093*	
H7B_2	0.387822	0.977487	0.425204	0.093*	
C8_2	0.4811 (6)	1.0590 (3)	0.4177 (3)	0.076 (2)	
H8A_2	0.551330	1.076608	0.426470	0.091*	
H8B_2	0.472960	1.045668	0.386048	0.091*	
C9_2	0.4285 (7)	1.1142 (3)	0.4283 (2)	0.085 (2)	
H9A_2	0.437649	1.128611	0.459828	0.103*	
H9B_2	0.358070	1.096928	0.419814	0.103*	
C10_2	0.4551 (6)	1.1738 (3)	0.4089 (2)	0.077 (2)	
H10A_2	0.526518	1.188504	0.415568	0.093*	
H10B_2	0.441738	1.159339	0.377397	0.093*	
C11_2	0.4101 (9)	1.2315 (4)	0.4203 (3)	0.114 (4)	
H11A_2	0.436262	1.266056	0.404903	0.172*	
H11B_2	0.424188	1.248246	0.451289	0.172*	
H11C_2	0.339388	1.219202	0.412621	0.172*	
N1_3	0.6896 (3)	0.51459 (19)	0.41226 (13)	0.0392 (9)	
N2_3	0.7044 (3)	0.57861 (19)	0.43335 (13)	0.0412 (10)	
C1_3	0.7387 (4)	0.5132 (3)	0.37773 (16)	0.0449 (13)	
H1_3	0.739761	0.474480	0.357126	0.054*	
C2_3	0.7875 (4)	0.5759 (3)	0.37635 (18)	0.0525 (14)	
C3_3	0.7628 (4)	0.6152 (3)	0.41210 (18)	0.0504 (14)	
H3_3	0.784440	0.661751	0.420338	0.061*	
C4_3	0.8515 (5)	0.5958 (3)	0.3430 (2)	0.0705 (17)	0.713 (8)
H4A_3	0.912889	0.578360	0.346918	0.085*	0.713 (8)
H4B_3	0.818012	0.574175	0.314297	0.085*	0.713 (8)
C5_3	0.8765 (7)	0.6686 (4)	0.3440 (3)	0.062 (2)	0.713 (8)
H5A_3	0.924423	0.676065	0.323332	0.075*	0.713 (8)
H5B_3	0.908638	0.690469	0.372883	0.075*	0.713 (8)
C6_3	0.7900 (8)	0.7024 (5)	0.3333 (4)	0.074 (2)	0.713 (8)
H6A_3	0.757364	0.678987	0.304730	0.089*	0.713 (8)
H6B_3	0.743048	0.694700	0.354226	0.089*	0.713 (8)
C7_3	0.8067 (9)	0.7722 (5)	0.3325 (4)	0.087 (3)	0.713 (8)
H7A_3	0.854185	0.779931	0.311754	0.104*	0.713 (8)
H7B_3	0.838921	0.795607	0.361127	0.104*	0.713 (8)
C8_3	0.7230 (10)	0.8056 (5)	0.3218 (5)	0.107 (3)	0.713 (8)
H8A_3	0.672304	0.795778	0.340831	0.128*	0.713 (8)
H8B_3	0.694202	0.786358	0.291950	0.128*	0.713 (8)
C9_3	0.7502 (11)	0.8814 (6)	0.3261 (4)	0.108 (3)	0.713 (8)
H9A_3	0.776850	0.901598	0.356114	0.129*	0.713 (8)
H9B_3	0.801148	0.892071	0.307374	0.129*	0.713 (8)
C10_3	0.6600 (12)	0.9101 (8)	0.3135 (4)	0.122 (4)	0.713 (8)
H10A_3	0.603479	0.887930	0.325563	0.147*	0.713 (8)
H10B_3	0.671072	0.958003	0.326389	0.147*	0.713 (8)
C11_3	0.6364 (14)	0.9021 (10)	0.2667 (5)	0.132 (6)	0.713 (8)
H11A_3	0.694826	0.918545	0.254048	0.199*	0.713 (8)
H11B_3	0.585062	0.927472	0.260621	0.199*	0.713 (8)
H11C_3	0.613605	0.855037	0.254390	0.199*	0.713 (8)
C4B_3	0.8515 (5)	0.5958 (3)	0.3430 (2)	0.0705 (17)	0.287 (8)
H4BA_3	0.920291	0.602377	0.355634	0.085*	0.287 (8)
H4BB_3	0.841669	0.558045	0.318583	0.085*	0.287 (8)
C5B_3	0.837 (2)	0.6557 (9)	0.3259 (7)	0.074 (3)	0.287 (8)
H5BA_3	0.778433	0.642021	0.304792	0.089*	0.287 (8)
H5BB_3	0.892921	0.665307	0.309250	0.089*	0.287 (8)
C6B_3	0.827 (2)	0.7203 (9)	0.3514 (9)	0.075 (4)	0.287 (8)
H6BA_3	0.756640	0.716878	0.354232	0.090*	0.287 (8)
H6BB_3	0.859399	0.721263	0.380418	0.090*	0.287 (8)
C7B_3	0.858 (2)	0.7869 (10)	0.3425 (10)	0.090 (4)	0.287 (8)
H7BA_3	0.871564	0.782113	0.312184	0.109*	0.287 (8)
H7BB_3	0.920555	0.806690	0.360469	0.109*	0.287 (8)
C8B_3	0.789 (2)	0.8354 (12)	0.3497 (8)	0.094 (4)	0.287 (8)
H8BA_3	0.825987	0.879728	0.363325	0.112*	0.287 (8)
H8BB_3	0.742421	0.820905	0.369532	0.112*	0.287 (8)
C9B_3	0.732 (2)	0.8403 (19)	0.3076 (9)	0.112 (4)	0.287 (8)
H9BA_3	0.775296	0.868645	0.292492	0.134*	0.287 (8)
H9BB_3	0.716389	0.795403	0.289516	0.134*	0.287 (8)
C10B_3	0.638 (2)	0.8675 (19)	0.3105 (10)	0.118 (4)	0.287 (8)
H10C_3	0.587723	0.834128	0.318699	0.142*	0.287 (8)
H10D_3	0.648890	0.907768	0.333222	0.142*	0.287 (8)
C11B_3	0.601 (3)	0.885 (3)	0.2696 (14)	0.134 (9)	0.287 (8)
H11D_3	0.649341	0.880245	0.249281	0.201*	0.287 (8)
H11E_3	0.588925	0.930333	0.274873	0.201*	0.287 (8)
H11F_3	0.539853	0.854286	0.257704	0.201*	0.287 (8)
N1_4	0.5134 (3)	0.4094 (2)	0.37865 (13)	0.0420 (10)	
N2_4	0.4148 (3)	0.4026 (2)	0.37910 (12)	0.0405 (9)	
C1_4	0.5300 (4)	0.3687 (2)	0.34324 (16)	0.0477 (13)	
H1_4	0.592838	0.363979	0.335249	0.057*	
C2_4	0.4431 (4)	0.3347 (2)	0.32010 (16)	0.0510 (14)	
C3_4	0.3728 (4)	0.3578 (2)	0.34422 (16)	0.0479 (13)	
H3_4	0.304368	0.343931	0.337026	0.057*	
C4_4	0.4266 (5)	0.2824 (3)	0.27963 (19)	0.0637 (15)	0.507 (8)
H4A_4	0.468412	0.297842	0.258464	0.076*	0.507 (8)
H4B_4	0.357731	0.276088	0.267103	0.076*	0.507 (8)
C5_4	0.4499 (13)	0.2160 (8)	0.2885 (11)	0.066 (3)	0.507 (8)
H5A_4	0.406684	0.202135	0.309629	0.079*	0.507 (8)
H5B_4	0.429991	0.183074	0.261568	0.079*	0.507 (8)
C6_4	0.5526 (10)	0.2092 (8)	0.3046 (5)	0.070 (3)	0.507 (8)
H6A_4	0.551446	0.163542	0.310092	0.084*	0.507 (8)
H6B_4	0.573280	0.240251	0.332307	0.084*	0.507 (8)
C7_4	0.6280 (9)	0.2230 (7)	0.2738 (4)	0.076 (3)	0.507 (8)
H7A_4	0.604656	0.193713	0.245705	0.091*	0.507 (8)
H7B_4	0.631000	0.269384	0.269670	0.091*	0.507 (8)
C8_4	0.7308 (10)	0.2132 (7)	0.2874 (5)	0.082 (3)	0.507 (8)
H8A_4	0.775039	0.230094	0.267494	0.099*	0.507 (8)
H8B_4	0.753092	0.239731	0.316427	0.099*	0.507 (8)
C9_4	0.7378 (12)	0.1417 (8)	0.2877 (7)	0.096 (4)	0.507 (8)
H9A_4	0.704486	0.113913	0.260454	0.115*	0.507 (8)
H9B_4	0.703639	0.127213	0.311379	0.115*	0.507 (8)
C10_4	0.8430 (13)	0.1309 (9)	0.2933 (9)	0.106 (4)	0.507 (8)
H10A_4	0.877486	0.146700	0.270124	0.127*	0.507 (8)
H10B_4	0.875601	0.157833	0.320940	0.127*	0.507 (8)
C11_4	0.852 (2)	0.0587 (10)	0.2925 (9)	0.129 (6)	0.507 (8)
H11A_4	0.814436	0.031050	0.266410	0.194*	0.507 (8)
H11B_4	0.825695	0.044395	0.317695	0.194*	0.507 (8)
H11C_4	0.920386	0.053989	0.292894	0.194*	0.507 (8)
C4B_4	0.4266 (5)	0.2824 (3)	0.27963 (19)	0.0637 (15)	0.493 (8)
H4BA_4	0.467967	0.299040	0.258675	0.076*	0.493 (8)
H4BB_4	0.357774	0.277299	0.267316	0.076*	0.493 (8)
C5B_4	0.4473 (14)	0.2145 (9)	0.2843 (12)	0.067 (3)	0.493 (8)
H5BA_4	0.414575	0.180550	0.259451	0.080*	0.493 (8)
H5BB_4	0.423536	0.202137	0.310758	0.080*	0.493 (8)
C6B_4	0.5573 (12)	0.2193 (7)	0.2866 (6)	0.073 (3)	0.493 (8)
H6BA_4	0.580484	0.237395	0.261755	0.087*	0.493 (8)
H6BB_4	0.588169	0.250716	0.312968	0.087*	0.493 (8)
C7B_4	0.5893 (10)	0.1537 (6)	0.2867 (5)	0.077 (3)	0.493 (8)
H7BA_4	0.557340	0.121803	0.260719	0.092*	0.493 (8)
H7BB_4	0.568247	0.136147	0.312030	0.092*	0.493 (8)
C8B_4	0.6979 (11)	0.1595 (8)	0.2877 (6)	0.086 (3)	0.493 (8)
H8BA_4	0.730380	0.195578	0.311402	0.103*	0.493 (8)
H8BB_4	0.718258	0.170969	0.260450	0.103*	0.493 (8)
C9B_4	0.7295 (12)	0.0944 (8)	0.2938 (6)	0.100 (4)	0.493 (8)
H9BA_4	0.701051	0.079799	0.318747	0.120*	0.493 (8)
H9BB_4	0.704080	0.059807	0.268134	0.120*	0.493 (8)
C10B_4	0.8400 (13)	0.1014 (11)	0.3010 (9)	0.110 (4)	0.493 (8)
H10C_4	0.868193	0.113847	0.275412	0.132*	0.493 (8)
H10D_4	0.865705	0.137583	0.325758	0.132*	0.493 (8)
C11B_4	0.872 (2)	0.0380 (11)	0.3093 (10)	0.133 (7)	0.493 (8)
H11D_4	0.943265	0.044746	0.313901	0.199*	0.493 (8)
H11E_4	0.848080	0.002251	0.284523	0.199*	0.493 (8)
H11F_4	0.844926	0.025771	0.334831	0.199*	0.493 (8)
N1_5	0.2610 (3)	0.3797 (2)	0.43639 (14)	0.0485 (11)	
N2_5	0.2660 (3)	0.3543 (2)	0.47286 (14)	0.0474 (11)	
C1_5	0.1861 (4)	0.3415 (3)	0.40947 (19)	0.0625 (17)	
H1_5	0.166750	0.348623	0.381586	0.075*	
C2_5	0.1415 (5)	0.2909 (3)	0.4276 (2)	0.0687 (19)	
C3_5	0.1951 (4)	0.3009 (3)	0.46796 (19)	0.0617 (17)	
H3_5	0.183117	0.273931	0.488836	0.074*	
C4_5	0.0595 (6)	0.2341 (4)	0.4079 (2)	0.097 (2)	0.518 (9)
H4A_5	0.035976	0.211243	0.430844	0.116*	0.518 (9)
H4B_5	0.004618	0.252673	0.395726	0.116*	0.518 (9)
C5_5	0.0871 (15)	0.1842 (8)	0.3735 (4)	0.103 (3)	0.518 (9)
H5A_5	0.033725	0.145180	0.366462	0.124*	0.518 (9)
H5B_5	0.145991	0.168969	0.384850	0.124*	0.518 (9)
C6_5	0.1073 (17)	0.2087 (9)	0.3327 (5)	0.118 (4)	0.518 (9)
H6A_5	0.049567	0.224679	0.320876	0.142*	0.518 (9)
H6B_5	0.163159	0.246004	0.338610	0.142*	0.518 (9)
C7_5	0.1302 (16)	0.1526 (9)	0.3009 (5)	0.132 (4)	0.518 (9)
H7A_5	0.182612	0.133904	0.314941	0.158*	0.518 (9)
H7B_5	0.071624	0.117289	0.294200	0.158*	0.518 (9)
C8_5	0.1610 (16)	0.1683 (14)	0.2600 (5)	0.150 (5)	0.518 (9)
H8A_5	0.210545	0.209421	0.267799	0.180*	0.518 (9)
H8B_5	0.196136	0.132603	0.248280	0.180*	0.518 (9)
C9_5	0.0977 (18)	0.1777 (13)	0.2246 (6)	0.168 (5)	0.518 (9)
H9A_5	0.091481	0.225106	0.228348	0.202*	0.518 (9)
H9B_5	0.032025	0.151589	0.224796	0.202*	0.518 (9)
C10_5	0.135 (2)	0.1558 (12)	0.1788 (6)	0.170 (6)	0.518 (9)
H10A_5	0.198466	0.140141	0.182154	0.204*	0.518 (9)
H10B_5	0.087105	0.120238	0.160062	0.204*	0.518 (9)
C11_5	0.147 (2)	0.2181 (14)	0.1612 (7)	0.172 (9)	0.518 (9)
H11A_5	0.165417	0.209180	0.132087	0.258*	0.518 (9)
H11B_5	0.197038	0.251647	0.179470	0.258*	0.518 (9)
H11C_5	0.084455	0.234400	0.160346	0.258*	0.518 (9)
C4B_5	0.0595 (6)	0.2341 (4)	0.4079 (2)	0.097 (2)	0.482 (9)
H4BA_5	0.022857	0.246506	0.383393	0.116*	0.482 (9)
H4BB_5	0.013883	0.226570	0.429414	0.116*	0.482 (9)
C5B_5	0.0974 (15)	0.1688 (8)	0.3921 (6)	0.112 (4)	0.482 (9)
H5BA_5	0.134039	0.157105	0.416819	0.134*	0.482 (9)
H5BB_5	0.040436	0.132871	0.382108	0.134*	0.482 (9)
C6B_5	0.1622 (13)	0.1703 (10)	0.3565 (5)	0.121 (4)	0.482 (9)
H6BA_5	0.223066	0.202852	0.366535	0.146*	0.482 (9)
H6BB_5	0.179536	0.126036	0.347442	0.146*	0.482 (9)
C7B_5	0.1062 (16)	0.1901 (11)	0.3197 (5)	0.121 (4)	0.482 (9)
H7BA_5	0.039853	0.163150	0.312943	0.145*	0.482 (9)
H7BB_5	0.100890	0.237679	0.326237	0.145*	0.482 (9)
C8B_5	0.1656 (17)	0.1766 (13)	0.2836 (6)	0.138 (4)	0.482 (9)
H8BA_5	0.232054	0.203154	0.291222	0.166*	0.482 (9)
H8BB_5	0.171258	0.129021	0.277969	0.166*	0.482 (9)
C9B_5	0.1189 (17)	0.1939 (13)	0.2448 (6)	0.143 (5)	0.482 (9)
H9BA_5	0.125759	0.242639	0.247046	0.172*	0.482 (9)
H9BB_5	0.048871	0.173763	0.239090	0.172*	0.482 (9)
C10B_5	0.1784 (19)	0.1631 (13)	0.2094 (7)	0.159 (5)	0.482 (9)
H10C_5	0.244398	0.159238	0.221582	0.191*	0.482 (9)
H10D_5	0.143994	0.118948	0.193916	0.191*	0.482 (9)
C11B_5	0.182 (3)	0.2141 (15)	0.1813 (9)	0.181 (9)	0.482 (9)
H11D_5	0.198851	0.195071	0.153117	0.272*	0.482 (9)
H11E_5	0.232022	0.252718	0.194411	0.272*	0.482 (9)
H11F_5	0.118566	0.227834	0.178121	0.272*	0.482 (9)
N1_6	0.2965 (3)	0.5097 (2)	0.40595 (14)	0.0445 (10)	
N2_6	0.3033 (3)	0.5709 (2)	0.42987 (13)	0.0437 (10)	
C1_6	0.2331 (4)	0.5053 (3)	0.37117 (18)	0.0538 (15)	
H1_6	0.215142	0.467189	0.349018	0.065*	
C2_6	0.1977 (4)	0.5637 (3)	0.37204 (18)	0.0586 (15)	
C3_6	0.2433 (4)	0.6034 (3)	0.40987 (17)	0.0517 (14)	
H3_6	0.233669	0.647372	0.420248	0.062*	
C4_6	0.1244 (5)	0.5794 (4)	0.3395 (2)	0.077 (2)	
H4A_6	0.077805	0.603469	0.354623	0.092*	
H4B_6	0.087065	0.537322	0.322220	0.092*	
C5_6	0.1720 (6)	0.6210 (5)	0.3098 (2)	0.103 (3)	
H5A_6	0.122142	0.622212	0.286051	0.124*	
H5B_6	0.223019	0.598566	0.297032	0.124*	
C6_6	0.2184 (6)	0.6927 (5)	0.3301 (3)	0.096 (3)	
H6A_6	0.258688	0.691941	0.357060	0.115*	
H6B_6	0.262933	0.710159	0.310549	0.115*	
C7_6	0.1499 (7)	0.7400 (5)	0.3401 (3)	0.107 (3)	
H7A_6	0.104784	0.722415	0.359458	0.129*	
H7B_6	0.110306	0.741465	0.313178	0.129*	
C8_6	0.1969 (8)	0.8096 (5)	0.3606 (4)	0.130 (4)	
H8A_6	0.249322	0.825108	0.343744	0.156*	
H8B_6	0.227410	0.809518	0.389760	0.156*	
C9_6	0.1254 (11)	0.8586 (7)	0.3638 (5)	0.174 (6)	
H9A_6	0.093985	0.857150	0.334597	0.209*	
H9B_6	0.073604	0.842597	0.380840	0.209*	
C10_6	0.1651 (13)	0.9280 (7)	0.3828 (5)	0.204 (8)	
H10A_6	0.221996	0.942943	0.368107	0.245*	
H10B_6	0.188939	0.930682	0.413278	0.245*	
C11_6	0.0931 (15)	0.9758 (8)	0.3803 (6)	0.250 (11)	
H11A_6	0.064923	0.971121	0.350513	0.375*	
H11B_6	0.127251	1.021522	0.390732	0.375*	
H11C_6	0.040976	0.965475	0.398111	0.375*	
N1_7	0.6303 (3)	0.67132 (17)	0.07720 (12)	0.0333 (8)	
N2_7	0.5344 (3)	0.67596 (16)	0.08026 (12)	0.0318 (8)	
C1_7	0.6843 (4)	0.7266 (2)	0.10116 (16)	0.0367 (11)	
H1_7	0.753538	0.736145	0.104473	0.044*	
C2_7	0.6251 (4)	0.7680 (2)	0.12049 (15)	0.0402 (11)	
C3_7	0.5320 (4)	0.7340 (2)	0.10592 (15)	0.0367 (11)	
H3_7	0.473990	0.749628	0.113074	0.044*	
C4_7	0.6534 (4)	0.8355 (2)	0.14860 (18)	0.0531 (14)	
H4A_7	0.716887	0.837618	0.165584	0.064*	
H4B_7	0.604343	0.841272	0.168961	0.064*	
C5_7	0.6618 (5)	0.8924 (2)	0.1244 (2)	0.0647 (16)	0.450 (13)
H5A_7	0.598144	0.891808	0.108024	0.078*	0.450 (13)
H5B_7	0.710373	0.887029	0.103685	0.078*	0.450 (13)
C6_7	0.693 (4)	0.9593 (8)	0.1556 (8)	0.072 (3)	0.450 (13)
H6A_7	0.639185	0.967788	0.172753	0.087*	0.450 (13)
H6B_7	0.749855	0.956278	0.175542	0.087*	0.450 (13)
C7_7	0.7203 (19)	1.0168 (8)	0.1334 (8)	0.081 (4)	0.450 (13)
H7A_7	0.663461	1.019200	0.113312	0.097*	0.450 (13)
H7B_7	0.773889	1.007640	0.116030	0.097*	0.450 (13)
C8_7	0.752 (2)	1.0837 (8)	0.1624 (8)	0.092 (4)	0.450 (13)
H8A_7	0.810177	1.082204	0.181891	0.110*	0.450 (13)
H8B_7	0.699136	1.092867	0.180274	0.110*	0.450 (13)
C9_7	0.775 (2)	1.1398 (8)	0.1381 (7)	0.101 (4)	0.450 (13)
H9A_7	0.830284	1.131728	0.121315	0.122*	0.450 (13)
H9B_7	0.718173	1.140129	0.117767	0.122*	0.450 (13)
C10_7	0.803 (3)	1.2068 (9)	0.1676 (7)	0.116 (5)	0.450 (13)
H10A_7	0.745968	1.217337	0.182323	0.139*	0.450 (13)
H10B_7	0.856422	1.205724	0.189634	0.139*	0.450 (13)
C11_7	0.834 (2)	1.2594 (10)	0.1422 (8)	0.135 (7)	0.450 (13)
H11A_7	0.884208	1.294218	0.159899	0.202*	0.450 (13)
H11B_7	0.778113	1.278755	0.133402	0.202*	0.450 (13)
H11C_7	0.861582	1.239578	0.116684	0.202*	0.450 (13)
C5B_7	0.6618 (5)	0.8924 (2)	0.1244 (2)	0.0647 (16)	0.550 (13)
H5BA_7	0.598083	0.890067	0.107566	0.078*	0.550 (13)
H5BB_7	0.710102	0.885827	0.103689	0.078*	0.550 (13)
C6B_7	0.692 (4)	0.9622 (6)	0.1515 (7)	0.074 (3)	0.550 (13)
H6BA_7	0.648425	0.967454	0.174483	0.089*	0.550 (13)
H6BB_7	0.759170	0.967059	0.165398	0.089*	0.550 (13)
C7B_7	0.6867 (16)	1.0168 (7)	0.1261 (7)	0.081 (3)	0.550 (13)
H7BA_7	0.618408	1.013243	0.113520	0.097*	0.550 (13)
H7BB_7	0.726820	1.009763	0.102041	0.097*	0.550 (13)
C8B_7	0.7212 (18)	1.0863 (7)	0.1516 (7)	0.094 (4)	0.550 (13)
H8BA_7	0.678534	1.093993	0.174599	0.113*	0.550 (13)
H8BB_7	0.787993	1.088728	0.165662	0.113*	0.550 (13)
C9B_7	0.7226 (16)	1.1416 (7)	0.1264 (6)	0.104 (4)	0.550 (13)
H9BA_7	0.655064	1.140645	0.113868	0.125*	0.550 (13)
H9BB_7	0.761819	1.132161	0.102356	0.125*	0.550 (13)
C10B_7	0.7627 (14)	1.2116 (7)	0.1509 (7)	0.114 (4)	0.550 (13)
H10C_7	0.754010	1.243734	0.131854	0.137*	0.550 (13)
H10D_7	0.725006	1.221632	0.175469	0.137*	0.550 (13)
C11B_7	0.8695 (13)	1.2203 (9)	0.1673 (7)	0.117 (6)	0.550 (13)
H11D_7	0.880023	1.184137	0.182286	0.176*	0.550 (13)
H11E_7	0.889246	1.262934	0.187089	0.176*	0.550 (13)
H11F_7	0.908449	1.219392	0.143077	0.176*	0.550 (13)
N1_8	0.3572 (3)	0.58370 (17)	0.09385 (11)	0.0291 (8)	
N2_8	0.3400 (3)	0.51946 (17)	0.09862 (12)	0.0343 (9)	
C1_8	0.3212 (3)	0.6196 (2)	0.12571 (15)	0.0356 (10)	
H1_8	0.323800	0.666297	0.129562	0.043*	
C2_8	0.2794 (3)	0.5797 (2)	0.15246 (14)	0.0356 (10)	
C3_8	0.2937 (4)	0.5172 (2)	0.13372 (15)	0.0392 (11)	
H3_8	0.273330	0.477787	0.144324	0.047*	
C4_8	0.2285 (4)	0.5966 (3)	0.19177 (17)	0.0468 (13)	
H4A_8	0.270839	0.591462	0.217065	0.056*	
H4B_8	0.167719	0.563681	0.189179	0.056*	
C5_8	0.2027 (4)	0.6655 (2)	0.20021 (17)	0.0476 (13)	
H5A_8	0.172492	0.674077	0.172949	0.057*	
H5B_8	0.152622	0.665730	0.220445	0.057*	
C6_8	0.2854 (4)	0.7222 (3)	0.21812 (19)	0.0548 (14)	
H6A_8	0.330949	0.726876	0.196203	0.066*	
H6B_8	0.321704	0.711353	0.243282	0.066*	
C7_8	0.2516 (5)	0.7879 (3)	0.2315 (2)	0.0630 (16)	
H7A_8	0.218811	0.799613	0.205825	0.076*	
H7B_8	0.202541	0.781874	0.251794	0.076*	
C8_8	0.3303 (5)	0.8449 (3)	0.2520 (2)	0.0743 (19)	
H8A_8	0.379933	0.850928	0.231955	0.089*	
H8B_8	0.362477	0.833865	0.278065	0.089*	
C9_8	0.2932 (6)	0.9099 (3)	0.2643 (3)	0.090 (2)	
H9A_8	0.260191	0.920368	0.238199	0.108*	
H9B_8	0.243879	0.903701	0.284460	0.108*	
C10_8	0.3710 (7)	0.9680 (3)	0.2845 (3)	0.108 (3)	
H10A_8	0.421691	0.973493	0.264880	0.129*	
H10B_8	0.402155	0.958684	0.311349	0.129*	
C11_8	0.3311 (9)	1.0324 (4)	0.2946 (4)	0.135 (4)	
H11A_8	0.308605	1.045208	0.267763	0.203*	
H11B_8	0.382654	1.067481	0.310822	0.203*	
H11C_8	0.276394	1.025848	0.311638	0.203*	
N1_9	0.7461 (3)	0.56243 (17)	0.08225 (12)	0.0330 (8)	
N2_9	0.7235 (3)	0.49931 (17)	0.08887 (12)	0.0311 (8)	
C1_9	0.8205 (3)	0.5943 (2)	0.11106 (15)	0.0382 (11)	
H1_9	0.850136	0.639375	0.113125	0.046*	
C2_9	0.8490 (3)	0.5525 (2)	0.13773 (14)	0.0374 (10)	
C3_9	0.7850 (4)	0.4935 (2)	0.12216 (15)	0.0388 (11)	
H3_9	0.784746	0.454101	0.133534	0.047*	
C4_9	0.9262 (4)	0.5685 (3)	0.17575 (16)	0.0495 (13)	
H4A_9	0.982443	0.599315	0.169426	0.059*	
H4B_9	0.949191	0.527138	0.180032	0.059*	
C5_9	0.8903 (4)	0.6002 (3)	0.21768 (18)	0.0582 (15)	
H5A_9	0.833789	0.569393	0.223838	0.070*	
H5B_9	0.942860	0.605581	0.241493	0.070*	
C6_9	0.8606 (4)	0.6669 (3)	0.2167 (2)	0.0634 (17)	
H6A_9	0.814276	0.662488	0.190667	0.076*	
H6B_9	0.825171	0.678336	0.241802	0.076*	
C7_9	0.9423 (5)	0.7239 (3)	0.2169 (2)	0.0658 (18)	
H7A_9	0.987821	0.729745	0.243371	0.079*	
H7B_9	0.978920	0.712592	0.192179	0.079*	
C8_9	0.9078 (5)	0.7884 (3)	0.2147 (2)	0.0754 (19)	
H8A_9	0.866116	0.797322	0.237947	0.090*	
H8B_9	0.866803	0.783380	0.187121	0.090*	
C9_9	0.9882 (5)	0.8480 (3)	0.2187 (3)	0.080 (2)	
H9A_9	1.030117	0.839149	0.195578	0.096*	
H9B_9	1.029104	0.853287	0.246414	0.096*	
C10_9	0.9522 (7)	0.9124 (4)	0.2163 (3)	0.103 (3)	
H10A_9	0.909676	0.906706	0.189002	0.123*	
H10B_9	0.912202	0.922075	0.240022	0.123*	
C11_9	1.0325 (8)	0.9704 (5)	0.2189 (4)	0.136 (4)	
H11A_9	1.075662	0.959908	0.196894	0.204*	
H11B_9	1.070040	0.980067	0.247281	0.204*	
H11C_9	1.004484	1.009284	0.214083	0.204*	
N1_10	0.4490 (3)	0.40508 (18)	0.09811 (12)	0.0363 (9)	
N2_10	0.5447 (3)	0.40267 (18)	0.09690 (12)	0.0358 (9)	
C1_10	0.4139 (4)	0.3625 (2)	0.12268 (16)	0.0429 (12)	
H1_10	0.348011	0.354522	0.128618	0.052*	
C2_10	0.4866 (4)	0.3321 (2)	0.13813 (15)	0.0439 (12)	
C3_10	0.5678 (4)	0.3594 (2)	0.12111 (16)	0.0434 (12)	
H3_10	0.631153	0.348962	0.125901	0.052*	
C4_10	0.4777 (6)	0.2762 (2)	0.16318 (19)	0.0629 (16)	0.524 (11)
H4A_10	0.420095	0.277388	0.179229	0.075*	0.524 (11)
H4B_10	0.536198	0.282999	0.184281	0.075*	0.524 (11)
C5_10	0.4672 (18)	0.2082 (6)	0.1343 (7)	0.076 (3)	0.524 (11)
H5A_10	0.406532	0.200196	0.114314	0.092*	0.524 (11)
H5B_10	0.522800	0.207845	0.116933	0.092*	0.524 (11)
C6_10	0.4643 (18)	0.1526 (7)	0.1599 (8)	0.093 (4)	0.524 (11)
H6A_10	0.528567	0.157924	0.176929	0.112*	0.524 (11)
H6B_10	0.415099	0.157595	0.180253	0.112*	0.524 (11)
C7_10	0.4409 (18)	0.0839 (7)	0.1337 (7)	0.110 (4)	0.524 (11)
H7A_10	0.492003	0.077765	0.114387	0.132*	0.524 (11)
H7B_10	0.378091	0.078953	0.115560	0.132*	0.524 (11)
C8_10	0.4338 (19)	0.0302 (7)	0.1606 (7)	0.129 (4)	0.524 (11)
H8A_10	0.497271	0.033379	0.177800	0.155*	0.524 (11)
H8B_10	0.384028	0.036613	0.180543	0.155*	0.524 (11)
C9_10	0.4069 (19)	−0.0361 (8)	0.1326 (9)	0.156 (5)	0.524 (11)
H9A_10	0.458867	−0.061530	0.138872	0.188*	0.524 (11)
H9B_10	0.409768	−0.029377	0.102674	0.188*	0.524 (11)
C10_10	0.3071 (16)	−0.0821 (11)	0.1336 (11)	0.174 (6)	0.524 (11)
H10A_10	0.276300	−0.071505	0.160237	0.209*	0.524 (11)
H10B_10	0.259947	−0.087103	0.107781	0.209*	0.524 (11)
C11_10	0.367 (2)	−0.1386 (11)	0.1330 (11)	0.169 (8)	0.524 (11)
H11A_10	0.394225	−0.146500	0.105567	0.253*	0.524 (11)
H11B_10	0.324855	−0.179184	0.136591	0.253*	0.524 (11)
H11C_10	0.420457	−0.125871	0.156459	0.253*	0.524 (11)
C4B_10	0.4777 (6)	0.2762 (2)	0.16318 (19)	0.0629 (16)	0.476 (11)
H4BA_10	0.542577	0.275221	0.177854	0.075*	0.476 (11)
H4BB_10	0.433569	0.284901	0.185445	0.075*	0.476 (11)
C5B_10	0.4388 (19)	0.2093 (7)	0.1350 (7)	0.074 (3)	0.476 (11)
H5BA_10	0.483226	0.201305	0.112815	0.089*	0.476 (11)
H5BB_10	0.374464	0.211192	0.120109	0.089*	0.476 (11)
C6B_10	0.4270 (19)	0.1507 (8)	0.1578 (8)	0.091 (4)	0.476 (11)
H6BA_10	0.491637	0.146930	0.171420	0.109*	0.476 (11)
H6BB_10	0.384586	0.159061	0.180768	0.109*	0.476 (11)
C7B_10	0.384 (2)	0.0864 (8)	0.1285 (7)	0.111 (4)	0.476 (11)
H7BA_10	0.322406	0.091590	0.112755	0.133*	0.476 (11)
H7BB_10	0.429531	0.075951	0.107179	0.133*	0.476 (11)
C8B_10	0.363 (2)	0.0295 (8)	0.1513 (7)	0.133 (4)	0.476 (11)
H8BA_10	0.313631	0.038465	0.170769	0.160*	0.476 (11)
H8BB_10	0.423777	0.027164	0.169158	0.160*	0.476 (11)
C9B_10	0.329 (2)	−0.0366 (9)	0.1228 (8)	0.158 (5)	0.476 (11)
H9BA_10	0.356881	−0.038954	0.095240	0.190*	0.476 (11)
H9BB_10	0.256515	−0.045841	0.116954	0.190*	0.476 (11)
C10B_10	0.367 (2)	−0.0882 (10)	0.1502 (10)	0.168 (6)	0.476 (11)
H10C_10	0.435990	−0.092195	0.147847	0.201*	0.476 (11)
H10D_10	0.353913	−0.080101	0.180503	0.201*	0.476 (11)
C11B_10	0.294 (2)	−0.1447 (12)	0.1216 (11)	0.179 (9)	0.476 (11)
H11D_10	0.318661	−0.156179	0.093826	0.268*	0.476 (11)
H11E_10	0.231084	−0.130490	0.117232	0.268*	0.476 (11)
H11F_10	0.286394	−0.183557	0.135409	0.268*	0.476 (11)
N1_11	0.2694 (3)	0.39052 (19)	0.03687 (13)	0.0376 (9)	
N2_11	0.2529 (3)	0.36822 (18)	−0.00618 (13)	0.0353 (9)	
C1_11	0.2012 (4)	0.3550 (2)	0.05483 (16)	0.0439 (12)	
H1_11	0.196300	0.360610	0.084744	0.053*	
C2_11	0.1392 (3)	0.3090 (2)	0.02399 (16)	0.0383 (11)	
C3_11	0.1756 (4)	0.3185 (2)	−0.01373 (16)	0.0429 (12)	
H3_11	0.149782	0.293435	−0.041269	0.051*	
C4_11	0.0572 (4)	0.2561 (2)	0.03096 (18)	0.0461 (12)	
H4A_11	0.006692	0.246267	0.005959	0.055*	
H4B_11	0.026911	0.273345	0.056568	0.055*	
C5_11	0.0919 (4)	0.1921 (2)	0.03707 (19)	0.0499 (13)	
H5A_11	0.115934	0.172527	0.010262	0.060*	
H5B_11	0.147406	0.202814	0.059996	0.060*	
C6_11	0.0139 (4)	0.1409 (2)	0.04864 (19)	0.0513 (13)	
H6A_11	−0.041080	0.129438	0.025460	0.062*	
H6B_11	−0.011017	0.160656	0.075178	0.062*	
C7_11	0.0499 (4)	0.0780 (3)	0.0554 (2)	0.0572 (15)	
H7A_11	0.072288	0.057408	0.028474	0.069*	
H7B_11	0.106840	0.089872	0.077680	0.069*	
C8_11	−0.0259 (5)	0.0275 (3)	0.0689 (2)	0.0616 (16)	
H8A_11	−0.082886	0.015958	0.046635	0.074*	
H8B_11	−0.048120	0.048241	0.095804	0.074*	
C9_11	0.0082 (5)	−0.0362 (3)	0.0757 (2)	0.0692 (18)	
H9A_11	0.031599	−0.056752	0.049018	0.083*	
H9B_11	0.064125	−0.025035	0.098498	0.083*	
C10_11	−0.0700 (6)	−0.0861 (3)	0.0882 (3)	0.085 (2)	
H10A_11	−0.092262	−0.065982	0.115310	0.103*	
H10B_11	−0.126550	−0.096533	0.065778	0.103*	
C11_11	−0.0362 (8)	−0.1496 (3)	0.0940 (3)	0.115 (4)	
H11A_11	−0.089003	−0.179084	0.103175	0.172*	
H11B_11	0.020512	−0.139658	0.115732	0.172*	
H11C_11	−0.018223	−0.171405	0.066705	0.172*	
N1_12	0.6660 (3)	0.36680 (18)	0.02623 (12)	0.0343 (9)	
N2_12	0.6426 (3)	0.34603 (17)	−0.01683 (12)	0.0328 (8)	
C1_12	0.7121 (4)	0.3212 (2)	0.04030 (15)	0.0385 (11)	
H1_12	0.736122	0.323672	0.069410	0.046*	
C2_12	0.7203 (3)	0.2704 (2)	0.00742 (15)	0.0353 (10)	
C3_12	0.6745 (3)	0.2886 (2)	−0.02785 (15)	0.0357 (10)	
H3_12	0.666704	0.263536	−0.056189	0.043*	
C4_12	0.7604 (3)	0.2078 (2)	0.00902 (16)	0.0375 (11)	
H4A_12	0.804188	0.213991	0.036012	0.045*	
H4B_12	0.799880	0.199628	−0.015101	0.045*	
C5_12	0.6815 (4)	0.1471 (2)	0.00653 (19)	0.0443 (12)	
H5A_12	0.634906	0.142772	−0.019389	0.053*	
H5B_12	0.645064	0.153872	0.031811	0.053*	
C6_12	0.7220 (4)	0.0827 (2)	0.0050 (2)	0.0474 (13)	
H6A_12	0.758656	0.076038	−0.020248	0.057*	
H6B_12	0.768356	0.086965	0.030935	0.057*	
C7_12	0.6431 (4)	0.0217 (2)	0.0023 (2)	0.0540 (14)	
H7A_12	0.608572	0.027249	0.028179	0.065*	
H7B_12	0.594986	0.018513	−0.022943	0.065*	
C8_12	0.6840 (4)	−0.0424 (2)	−0.0014 (2)	0.0545 (15)	
H8A_12	0.733411	−0.038294	0.023497	0.065*	
H8B_12	0.717833	−0.047781	−0.027540	0.065*	
C9_12	0.6091 (4)	−0.1049 (2)	−0.0034 (2)	0.0504 (14)	
H9A_12	0.577234	−0.100867	0.023320	0.061*	
H9B_12	0.558191	−0.108586	−0.027636	0.061*	
C10_12	0.6535 (4)	−0.1677 (2)	−0.0089 (2)	0.0518 (14)	
H10A_12	0.684604	−0.171451	−0.035742	0.062*	
H10B_12	0.705490	−0.162972	0.015098	0.062*	
C11_12	0.5830 (5)	−0.2313 (2)	−0.0103 (2)	0.0608 (16)	
H11A_12	0.532225	−0.237530	−0.034456	0.091*	
H11B_12	0.552874	−0.228893	0.016527	0.091*	
H11C_12	0.618160	−0.268804	−0.013737	0.091*	
N1_13	0.5182 (3)	0.5031 (2)	0.24963 (14)	0.0461 (10)	
C1_13	0.6147 (4)	0.4846 (3)	0.26588 (19)	0.0540 (14)	
H1A_13	0.665376	0.525111	0.271625	0.065*	
H1B_13	0.606894	0.469872	0.293533	0.065*	
C2_13	0.6510 (5)	0.4319 (4)	0.2368 (2)	0.0701 (18)	
H2A_13	0.660955	0.446615	0.209328	0.084*	
H2B_13	0.600775	0.391134	0.230718	0.084*	
C3_13	0.7452 (6)	0.4158 (4)	0.2554 (2)	0.080 (2)	
H3A_13	0.791519	0.457957	0.265673	0.096*	
H3B_13	0.732137	0.395393	0.280677	0.096*	
C4_13	0.7931 (8)	0.3715 (5)	0.2267 (3)	0.118 (4)	
H4A_13	0.750948	0.327883	0.218330	0.177*	
H4B_13	0.855225	0.366658	0.241486	0.177*	
H4C_13	0.805325	0.390245	0.201059	0.177*	
C5_13	0.4374 (4)	0.4457 (3)	0.2493 (2)	0.0579 (15)	
H5A_13	0.447294	0.428849	0.276332	0.069*	
H5B_13	0.442333	0.409516	0.225397	0.069*	
C6_13	0.3349 (4)	0.4616 (3)	0.2448 (2)	0.0619 (16)	
H6A_13	0.324644	0.491188	0.271326	0.074*	
H6B_13	0.327646	0.485478	0.220711	0.074*	
C7_13	0.2587 (5)	0.3994 (3)	0.2369 (3)	0.079 (2)	
H7A_13	0.271333	0.372918	0.259135	0.094*	
H7B_13	0.264478	0.372371	0.208714	0.094*	
C8_13	0.1561 (5)	0.4137 (4)	0.2374 (3)	0.096 (3)	
H8A_13	0.143455	0.440577	0.215735	0.143*	
H8B_13	0.148679	0.438100	0.265768	0.143*	
H8C_13	0.109474	0.371819	0.230946	0.143*	
C9_13	0.5051 (4)	0.5656 (3)	0.27943 (17)	0.0513 (13)	
H9A_13	0.558467	0.601715	0.276960	0.062*	
H9B_13	0.443088	0.578153	0.269109	0.062*	
C10_13	0.5035 (5)	0.5621 (3)	0.32650 (18)	0.0574 (15)	
H10A_13	0.463753	0.519613	0.329507	0.069*	
H10B_13	0.570637	0.564249	0.339971	0.069*	
C11_13	0.4611 (7)	0.6189 (4)	0.3484 (2)	0.085 (2)	
H11A_13	0.389844	0.609383	0.339675	0.102*	
H11B_13	0.470887	0.619967	0.379656	0.102*	
C12_13	0.4985 (8)	0.6855 (4)	0.3409 (3)	0.100 (3)	
H12A_13	0.486439	0.686515	0.310340	0.151*	
H12B_13	0.568809	0.696743	0.350086	0.151*	
H12C_13	0.465325	0.717924	0.357402	0.151*	
C13_13	0.5197 (4)	0.5182 (3)	0.20401 (17)	0.0524 (13)	
H13A_13	0.517048	0.475732	0.183903	0.063*	
H13B_13	0.459420	0.535285	0.196731	0.063*	
C14_13	0.6066 (4)	0.5675 (3)	0.19581 (18)	0.0516 (13)	
H14A_13	0.647755	0.586253	0.223339	0.062*	
H14B_13	0.646411	0.543244	0.176549	0.062*	
C15_13	0.5787 (5)	0.6231 (3)	0.17635 (18)	0.0550 (14)	
H15A_13	0.525334	0.604638	0.152878	0.066*	
H15B_13	0.635263	0.643374	0.163447	0.066*	
C16_13	0.5457 (6)	0.6774 (3)	0.2071 (2)	0.0663 (18)	
H16A_13	0.530511	0.712286	0.191765	0.100*	
H16B_13	0.597879	0.696443	0.230491	0.100*	
H16C_13	0.487312	0.658633	0.218922	0.100*	
N1_14	1.0384 (11)	0.5273 (7)	0.0132 (4)	0.047 (2)	0.5
C1_14	1.0014 (10)	0.5856 (8)	0.0386 (6)	0.049 (3)	0.5
H1A_14	0.954427	0.601194	0.019333	0.059*	0.5
H1B_14	0.966107	0.570286	0.061708	0.059*	0.5
C2_14	1.0817 (9)	0.6433 (6)	0.0583 (4)	0.052 (2)	0.5
H2A_14	1.136580	0.625864	0.071387	0.062*	0.5
H2B_14	1.105975	0.665663	0.035185	0.062*	0.5
C3_14	1.0508 (9)	0.6939 (5)	0.0918 (4)	0.050 (2)	0.5
H3A_14	0.993144	0.709189	0.079291	0.060*	0.5
H3B_14	1.031190	0.672436	0.115914	0.060*	0.5
C4_14	1.1297 (8)	0.7529 (5)	0.1086 (4)	0.055 (3)	0.5
H4A_14	1.187358	0.738121	0.120758	0.083*	0.5
H4B_14	1.106326	0.783313	0.130957	0.083*	0.5
H4C_14	1.146881	0.775948	0.085194	0.083*	0.5
C5_14	1.0991 (8)	0.4944 (5)	0.0422 (3)	0.048 (2)	0.5
H5A_14	1.110038	0.452299	0.024951	0.058*	0.5
H5B_14	1.163689	0.523434	0.050264	0.058*	0.5
C6_14	1.0613 (9)	0.4786 (6)	0.0829 (4)	0.052 (2)	0.5
H6A_14	1.037481	0.518169	0.097368	0.063*	0.5
H6B_14	1.117113	0.471952	0.101937	0.063*	0.5
C7_14	0.9811 (10)	0.4193 (6)	0.0791 (4)	0.053 (3)	0.5
H7A_14	0.990489	0.384614	0.055036	0.064*	0.5
H7B_14	0.917639	0.432711	0.072066	0.064*	0.5
C8_14	0.9772 (11)	0.3893 (7)	0.1201 (5)	0.060 (3)	0.5
H8A_14	0.917530	0.356249	0.117305	0.089*	0.5
H8B_14	0.977362	0.424674	0.144840	0.089*	0.5
H8C_14	1.034192	0.368072	0.124214	0.089*	0.5
C9_14	1.1002 (8)	0.5496 (5)	−0.0205 (3)	0.049 (2)	0.5
H9A_14	1.161265	0.577758	−0.005323	0.058*	0.5
H9B_14	1.118388	0.509598	−0.037341	0.058*	0.5
C10_14	1.0578 (8)	0.5867 (6)	−0.0511 (4)	0.054 (2)	0.5
H10A_14	1.112642	0.612023	−0.062502	0.064*	0.5
H10B_14	1.024360	0.619599	−0.034618	0.064*	0.5
C11_14	0.9870 (10)	0.5490 (7)	−0.0889 (4)	0.055 (3)	0.5
H11A_14	0.921112	0.540198	−0.079606	0.066*	0.5
H11B_14	1.006441	0.505636	−0.099008	0.066*	0.5
C12_14	0.9829 (10)	0.5867 (7)	−0.1261 (4)	0.053 (3)	0.5
H12A_14	0.944701	0.557477	−0.151409	0.080*	0.5
H12B_14	1.049188	0.601219	−0.132708	0.080*	0.5
H12C_14	0.952004	0.625567	−0.118019	0.080*	0.5
C13_14	0.9478 (14)	0.4781 (9)	−0.0079 (6)	0.048 (3)	0.5
H13A_14	0.909353	0.500261	−0.026754	0.058*	0.5
H13B_14	0.907297	0.466833	0.014751	0.058*	0.5
C14_14	0.9667 (9)	0.4129 (8)	−0.0347 (6)	0.052 (3)	0.5
H14A_14	1.013519	0.423392	−0.055240	0.062*	0.5
H14B_14	0.996039	0.386769	−0.015479	0.062*	0.5
C15_14	0.8752 (10)	0.3734 (6)	−0.0582 (5)	0.062 (3)	0.5
H15A_14	0.848483	0.398581	−0.078807	0.074*	0.5
H15B_14	0.826861	0.366115	−0.037755	0.074*	0.5
C16_14	0.8901 (10)	0.3063 (6)	−0.0825 (4)	0.059 (3)	0.5
H16A_14	0.830687	0.284967	−0.101541	0.089*	0.5
H16B_14	0.904420	0.278007	−0.062029	0.089*	0.5
H16C_14	0.944761	0.312993	−0.099589	0.089*	0.5
N1_15	1.0001 (15)	0.5214 (9)	0.5011 (7)	0.083 (2)	0.257 (5)
C1_15	1.0672 (17)	0.5430 (16)	0.5431 (7)	0.083 (3)	0.257 (5)
H1A_15	1.085157	0.503076	0.552516	0.099*	0.257 (5)
H1B_15	1.127869	0.570761	0.537904	0.099*	0.257 (5)
C2_15	1.023 (2)	0.5818 (18)	0.5789 (7)	0.077 (3)	0.257 (5)
H2A_15	1.020558	0.626801	0.572869	0.093*	0.257 (5)
H2B_15	0.954758	0.559553	0.579182	0.093*	0.257 (5)
C3_15	1.077 (2)	0.5879 (18)	0.6216 (8)	0.079 (3)	0.257 (5)
H3A_15	1.143688	0.612215	0.621225	0.094*	0.257 (5)
H3B_15	1.082919	0.542725	0.626202	0.094*	0.257 (5)
C4_15	1.034 (3)	0.622 (2)	0.6597 (10)	0.080 (4)	0.257 (5)
H4A_15	1.075868	0.665463	0.671408	0.119*	0.257 (5)
H4B_15	1.030986	0.594843	0.681719	0.119*	0.257 (5)
H4C_15	0.968604	0.629283	0.650455	0.119*	0.257 (5)
C5_15	0.9636 (18)	0.5776 (11)	0.4841 (10)	0.083 (3)	0.257 (5)
H5A_15	0.909903	0.590103	0.500340	0.099*	0.257 (5)
H5B_15	0.935921	0.561210	0.453784	0.099*	0.257 (5)
C6_15	1.039 (2)	0.6397 (15)	0.4862 (15)	0.080 (3)	0.257 (5)
H6A_15	1.064859	0.658052	0.516533	0.096*	0.257 (5)
H6B_15	1.094342	0.627649	0.470782	0.096*	0.257 (5)
C7_15	0.998 (2)	0.6923 (16)	0.4669 (15)	0.078 (4)	0.257 (5)
H7A_15	0.951526	0.670109	0.441502	0.094*	0.257 (5)
H7B_15	0.960774	0.715307	0.488038	0.094*	0.257 (5)
C8_15	1.071 (2)	0.7429 (14)	0.4537 (10)	0.078 (4)	0.257 (5)
H8A_15	1.088385	0.725307	0.425379	0.117*	0.257 (5)
H8B_15	1.129560	0.753572	0.474583	0.117*	0.257 (5)
H8C_15	1.043312	0.783107	0.452569	0.117*	0.257 (5)
C9_15	0.9126 (16)	0.4711 (11)	0.5071 (10)	0.083 (3)	0.257 (5)
H9A_15	0.893888	0.486440	0.535909	0.099*	0.257 (5)
H9B_15	0.858187	0.474877	0.486155	0.099*	0.257 (5)
C10_15	0.916 (2)	0.3995 (12)	0.5033 (10)	0.083 (3)	0.257 (5)
H10A_15	0.937244	0.384152	0.475107	0.100*	0.257 (5)
H10B_15	0.847704	0.376250	0.502630	0.100*	0.257 (5)
C11_15	0.977 (3)	0.3762 (18)	0.5359 (14)	0.081 (4)	0.257 (5)
H11A_15	0.963369	0.397351	0.564651	0.098*	0.257 (5)
H11B_15	1.045870	0.392233	0.533164	0.098*	0.257 (5)
C12_15	0.963 (6)	0.301 (2)	0.534 (2)	0.081 (4)	0.257 (5)
H12A_15	0.904573	0.286917	0.547703	0.121*	0.257 (5)
H12B_15	1.020084	0.290292	0.549464	0.121*	0.257 (5)
H12C_15	0.954690	0.278206	0.504211	0.121*	0.257 (5)
C13_15	1.0636 (18)	0.4895 (16)	0.4699 (7)	0.087 (3)	0.257 (5)
H13A_15	1.068800	0.444885	0.475876	0.104*	0.257 (5)
H13B_15	1.130001	0.516493	0.475273	0.104*	0.257 (5)
C14_15	1.026 (2)	0.4827 (14)	0.4226 (7)	0.088 (3)	0.257 (5)
H14A_15	0.990409	0.519514	0.419134	0.106*	0.257 (5)
H14B_15	1.081645	0.486073	0.405526	0.106*	0.257 (5)
C15_15	0.960 (3)	0.4194 (18)	0.4065 (10)	0.089 (4)	0.257 (5)
H15A_15	0.897103	0.422873	0.417716	0.107*	0.257 (5)
H15B_15	0.987528	0.385144	0.419505	0.107*	0.257 (5)
C16_15	0.939 (4)	0.393 (2)	0.3583 (10)	0.092 (4)	0.257 (5)
H16A_15	0.904011	0.422819	0.344920	0.138*	0.257 (5)
H16B_15	0.898782	0.348543	0.353195	0.138*	0.257 (5)
H16C_15	1.000513	0.390367	0.345973	0.138*	0.257 (5)
N1B_15	1.0102 (16)	0.5238 (9)	0.4988 (7)	0.083 (2)	0.243 (5)
C1B_15	0.9234 (17)	0.4874 (16)	0.4673 (8)	0.087 (3)	0.243 (5)
H1C_15	0.872523	0.470129	0.484307	0.105*	0.243 (5)
H1D_15	0.897536	0.521019	0.453177	0.105*	0.243 (5)
C2B_15	0.933 (2)	0.4307 (14)	0.4322 (8)	0.088 (3)	0.243 (5)
H2C_15	0.971866	0.400808	0.444297	0.106*	0.243 (5)
H2D_15	0.867543	0.404829	0.420950	0.106*	0.243 (5)
C3B_15	0.980 (3)	0.4544 (18)	0.3970 (9)	0.090 (4)	0.243 (5)
H3C_15	1.051757	0.462576	0.405523	0.108*	0.243 (5)
H3D_15	0.962126	0.498048	0.395061	0.108*	0.243 (5)
C4B_15	0.962 (4)	0.413 (2)	0.3527 (10)	0.088 (4)	0.243 (5)
H4D_15	0.903842	0.423227	0.337510	0.132*	0.243 (5)
H4E_15	0.951784	0.366107	0.354520	0.132*	0.243 (5)
H4F_15	1.018403	0.423658	0.337189	0.132*	0.243 (5)
C5B_15	1.0636 (19)	0.5847 (11)	0.4850 (11)	0.083 (3)	0.243 (5)
H5C_15	1.122751	0.602545	0.505531	0.100*	0.243 (5)
H5D_15	1.085553	0.570247	0.456671	0.100*	0.243 (5)
C6B_15	1.008 (2)	0.6408 (16)	0.4815 (17)	0.081 (3)	0.243 (5)
H6C_15	0.943613	0.622409	0.464991	0.097*	0.243 (5)
H6D_15	0.997202	0.661771	0.510601	0.097*	0.243 (5)
C7B_15	1.061 (3)	0.6933 (18)	0.4601 (17)	0.079 (4)	0.243 (5)
H7C_15	1.128845	0.706413	0.473835	0.094*	0.243 (5)
H7D_15	1.062878	0.673629	0.429636	0.094*	0.243 (5)
C8B_15	1.016 (2)	0.7539 (13)	0.4618 (12)	0.082 (4)	0.243 (5)
H8D_15	1.050395	0.783592	0.445120	0.122*	0.243 (5)
H8E_15	1.020186	0.776724	0.491697	0.122*	0.243 (5)
H8F_15	0.947068	0.741258	0.449860	0.122*	0.243 (5)
C9B_15	1.0848 (17)	0.4782 (11)	0.5045 (11)	0.084 (3)	0.243 (5)
H9C_15	1.128204	0.480876	0.481640	0.100*	0.243 (5)
H9D_15	1.125684	0.496668	0.532187	0.100*	0.243 (5)
C10B_15	1.049 (2)	0.4078 (12)	0.5040 (11)	0.084 (3)	0.243 (5)
H10C_15	1.106916	0.386744	0.507808	0.101*	0.243 (5)
H10D_15	1.016529	0.388548	0.474634	0.101*	0.243 (5)
C11B_15	0.981 (4)	0.385 (2)	0.5342 (16)	0.081 (4)	0.243 (5)
H11C_15	0.914695	0.392888	0.525292	0.097*	0.243 (5)
H11D_15	1.002432	0.411425	0.563269	0.097*	0.243 (5)
C12B_15	0.975 (5)	0.311 (2)	0.536 (3)	0.081 (4)	0.243 (5)
H12D_15	0.991093	0.287360	0.508816	0.121*	0.243 (5)
H12E_15	0.908769	0.292081	0.540677	0.121*	0.243 (5)
H12F_15	1.021432	0.306084	0.559531	0.121*	0.243 (5)
C13B_15	0.9720 (19)	0.5396 (16)	0.5423 (7)	0.082 (3)	0.243 (5)
H13C_15	0.908861	0.509464	0.540811	0.099*	0.243 (5)
H13D_15	0.957725	0.585240	0.545552	0.099*	0.243 (5)
C14B_15	1.034 (2)	0.5353 (18)	0.5844 (7)	0.077 (3)	0.243 (5)
H14C_15	1.020046	0.489975	0.590749	0.092*	0.243 (5)
H14D_15	1.104212	0.546975	0.581955	0.092*	0.243 (5)
C15B_15	1.0034 (19)	0.5842 (16)	0.6184 (9)	0.077 (3)	0.243 (5)
H15C_15	0.938953	0.564143	0.625426	0.093*	0.243 (5)
H15D_15	0.993883	0.623547	0.606034	0.093*	0.243 (5)
C16B_15	1.070 (3)	0.609 (2)	0.6604 (10)	0.077 (4)	0.243 (5)
H16D_15	1.090465	0.570519	0.670798	0.116*	0.243 (5)
H16E_15	1.034536	0.631823	0.681797	0.116*	0.243 (5)
H16F_15	1.127440	0.638935	0.655703	0.116*	0.243 (5)

### Bis(tetrabutylazanium) hexa-µ4-hydroxido-dodeca-µ2-(4-octylpyrazolato)-hexahedro-octanickel (3) Atomic displacement parameters (Å^2^)

**Table:**

	*U*^11^	*U*^22^	*U*^33^	*U*^12^	*U*^13^	*U*^23^
Ni1_16	0.0434 (5)	0.0400 (4)	0.0311 (4)	0.0155 (4)	0.0088 (4)	0.0110 (3)
Ni2_16	0.0398 (5)	0.0370 (4)	0.0337 (4)	0.0051 (3)	0.0102 (4)	0.0082 (3)
Ni3_16	0.0378 (4)	0.0371 (4)	0.0309 (4)	0.0103 (3)	0.0100 (3)	0.0088 (3)
Ni4_16	0.0355 (4)	0.0425 (4)	0.0308 (4)	0.0069 (3)	0.0049 (3)	0.0078 (3)
Ni5_16	0.0278 (4)	0.0272 (3)	0.0259 (4)	0.0054 (3)	0.0045 (3)	0.0038 (3)
Ni6_16	0.0313 (4)	0.0282 (4)	0.0278 (4)	0.0027 (3)	0.0077 (3)	0.0045 (3)
Ni7_16	0.0311 (4)	0.0275 (4)	0.0253 (4)	0.0052 (3)	0.0034 (3)	0.0049 (3)
Ni8_16	0.0260 (4)	0.0271 (3)	0.0288 (4)	0.0025 (3)	0.0031 (3)	0.0047 (3)
O1_16	0.0411 (18)	0.0338 (15)	0.0298 (17)	0.0094 (13)	0.0102 (14)	0.0098 (13)
O2_16	0.0298 (16)	0.0426 (17)	0.0321 (17)	0.0091 (13)	0.0068 (13)	0.0082 (14)
O3_16	0.0391 (18)	0.0310 (15)	0.0333 (17)	0.0069 (13)	0.0067 (14)	0.0077 (13)
O4_16	0.0311 (16)	0.0241 (13)	0.0225 (15)	0.0017 (11)	0.0022 (12)	0.0020 (11)
O5_16	0.0258 (15)	0.0297 (14)	0.0280 (16)	0.0062 (12)	0.0076 (12)	0.0022 (12)
O6_16	0.0268 (15)	0.0250 (13)	0.0287 (16)	0.0018 (11)	0.0026 (12)	0.0052 (12)
N1_1	0.063 (3)	0.052 (2)	0.038 (2)	0.028 (2)	0.014 (2)	0.013 (2)
N2_1	0.045 (2)	0.044 (2)	0.042 (2)	0.0146 (19)	0.016 (2)	0.0118 (19)
C1_1	0.091 (5)	0.059 (3)	0.046 (3)	0.046 (3)	0.024 (3)	0.018 (3)
C2_1	0.062 (4)	0.047 (3)	0.056 (4)	0.026 (3)	0.018 (3)	0.011 (3)
C3_1	0.064 (4)	0.061 (3)	0.041 (3)	0.033 (3)	0.025 (3)	0.015 (3)
C4_1	0.107 (6)	0.071 (4)	0.066 (4)	0.052 (4)	0.034 (4)	0.017 (3)
C5_1	0.087 (5)	0.049 (3)	0.102 (6)	0.027 (3)	−0.003 (4)	0.005 (4)
C6_1	0.102 (6)	0.067 (4)	0.097 (6)	0.041 (4)	−0.002 (5)	0.000 (4)
C7_1	0.123 (8)	0.066 (5)	0.135 (8)	0.038 (5)	−0.008 (6)	0.013 (5)
C8_1	0.128 (8)	0.074 (5)	0.155 (9)	0.046 (5)	−0.038 (7)	−0.005 (6)
C9_1	0.189 (13)	0.083 (6)	0.163 (11)	0.031 (7)	−0.025 (9)	0.033 (7)
C10_1	0.247 (17)	0.079 (7)	0.204 (14)	0.049 (9)	−0.039 (12)	0.025 (8)
C11_1	0.25 (2)	0.124 (11)	0.32 (2)	−0.008 (12)	0.008 (18)	0.087 (14)
N1_2	0.063 (3)	0.036 (2)	0.048 (3)	0.010 (2)	0.019 (2)	0.0138 (19)
N2_2	0.065 (3)	0.042 (2)	0.041 (2)	0.018 (2)	0.015 (2)	0.0135 (19)
C1_2	0.084 (5)	0.042 (3)	0.070 (4)	0.014 (3)	0.038 (4)	0.022 (3)
C2_2	0.109 (6)	0.046 (3)	0.082 (5)	0.032 (4)	0.051 (4)	0.032 (3)
C3_2	0.087 (5)	0.050 (3)	0.060 (4)	0.033 (3)	0.024 (3)	0.026 (3)
C4_2	0.158 (9)	0.062 (4)	0.183 (10)	0.057 (5)	0.107 (8)	0.072 (6)
C5_2	0.092 (5)	0.045 (3)	0.061 (4)	0.017 (3)	0.010 (3)	0.022 (3)
C6_2	0.126 (7)	0.045 (3)	0.108 (6)	0.030 (4)	0.046 (5)	0.036 (4)
C7_2	0.111 (6)	0.056 (4)	0.080 (5)	0.028 (4)	0.024 (4)	0.033 (3)
C8_2	0.097 (6)	0.043 (3)	0.096 (5)	0.020 (3)	0.020 (4)	0.025 (3)
C9_2	0.135 (7)	0.059 (4)	0.078 (5)	0.037 (4)	0.029 (5)	0.029 (4)
C10_2	0.104 (6)	0.047 (3)	0.083 (5)	0.016 (3)	0.011 (4)	0.017 (3)
C11_2	0.205 (12)	0.071 (5)	0.095 (6)	0.065 (6)	0.057 (7)	0.038 (5)
N1_3	0.041 (2)	0.042 (2)	0.037 (2)	0.0080 (17)	0.0118 (19)	0.0101 (17)
N2_3	0.042 (2)	0.042 (2)	0.041 (2)	0.0006 (18)	0.0123 (19)	0.0131 (18)
C1_3	0.049 (3)	0.050 (3)	0.039 (3)	0.014 (2)	0.017 (3)	0.010 (2)
C2_3	0.048 (3)	0.067 (3)	0.050 (3)	0.016 (3)	0.019 (3)	0.018 (3)
C3_3	0.048 (3)	0.050 (3)	0.053 (3)	−0.003 (2)	0.024 (3)	0.009 (3)
C4_3	0.061 (4)	0.088 (4)	0.068 (4)	0.004 (3)	0.040 (3)	0.021 (3)
C5_3	0.064 (5)	0.082 (5)	0.044 (4)	0.001 (4)	0.026 (4)	0.023 (4)
C6_3	0.090 (6)	0.079 (5)	0.059 (5)	0.007 (4)	0.027 (4)	0.026 (4)
C7_3	0.103 (6)	0.078 (5)	0.077 (5)	−0.003 (5)	0.013 (5)	0.025 (4)
C8_3	0.137 (7)	0.093 (6)	0.090 (6)	0.004 (6)	0.025 (6)	0.021 (5)
C9_3	0.150 (7)	0.083 (6)	0.085 (6)	0.012 (6)	0.009 (6)	0.011 (5)
C10_3	0.161 (8)	0.102 (7)	0.097 (6)	0.009 (7)	0.026 (7)	0.009 (6)
C11_3	0.152 (13)	0.123 (12)	0.113 (9)	0.016 (10)	0.004 (10)	0.010 (8)
C4B_3	0.061 (4)	0.088 (4)	0.068 (4)	0.004 (3)	0.040 (3)	0.021 (3)
C5B_3	0.075 (6)	0.087 (6)	0.063 (6)	0.003 (6)	0.034 (6)	0.022 (6)
C6B_3	0.086 (7)	0.077 (6)	0.063 (7)	0.003 (6)	0.025 (6)	0.022 (6)
C7B_3	0.107 (8)	0.083 (7)	0.081 (7)	0.005 (7)	0.015 (7)	0.022 (6)
C8B_3	0.125 (8)	0.076 (7)	0.079 (7)	0.011 (6)	0.013 (6)	0.020 (6)
C9B_3	0.146 (8)	0.091 (7)	0.094 (7)	0.009 (7)	0.021 (7)	0.014 (7)
C10B_3	0.153 (8)	0.097 (8)	0.101 (7)	0.009 (7)	0.017 (7)	0.015 (7)
C11B_3	0.158 (16)	0.112 (15)	0.119 (14)	0.010 (15)	0.004 (14)	0.004 (13)
N1_4	0.055 (3)	0.041 (2)	0.033 (2)	0.011 (2)	0.013 (2)	0.0071 (18)
N2_4	0.045 (2)	0.045 (2)	0.031 (2)	0.0050 (18)	0.0056 (18)	0.0070 (18)
C1_4	0.066 (4)	0.044 (3)	0.037 (3)	0.014 (3)	0.018 (3)	0.010 (2)
C2_4	0.076 (4)	0.044 (3)	0.034 (3)	0.012 (3)	0.008 (3)	0.008 (2)
C3_4	0.061 (4)	0.049 (3)	0.032 (3)	0.007 (3)	0.000 (2)	0.009 (2)
C4_4	0.092 (4)	0.054 (3)	0.042 (3)	0.015 (3)	0.008 (3)	−0.001 (2)
C5_4	0.088 (5)	0.054 (5)	0.054 (6)	0.019 (5)	0.006 (5)	−0.002 (4)
C6_4	0.090 (5)	0.063 (5)	0.057 (6)	0.023 (5)	0.013 (5)	0.003 (5)
C7_4	0.085 (6)	0.079 (5)	0.064 (5)	0.018 (5)	0.013 (5)	0.010 (5)
C8_4	0.079 (7)	0.089 (7)	0.078 (6)	0.020 (6)	0.009 (6)	0.013 (6)
C9_4	0.088 (6)	0.100 (7)	0.096 (6)	0.026 (7)	0.003 (6)	0.003 (7)
C10_4	0.091 (7)	0.110 (9)	0.114 (8)	0.027 (8)	0.003 (7)	0.012 (8)
C11_4	0.113 (11)	0.118 (13)	0.145 (13)	0.017 (11)	−0.008 (11)	0.005 (11)
C4B_4	0.092 (4)	0.054 (3)	0.042 (3)	0.015 (3)	0.008 (3)	−0.001 (2)
C5B_4	0.088 (5)	0.055 (5)	0.054 (6)	0.017 (5)	0.006 (5)	−0.002 (5)
C6B_4	0.091 (6)	0.065 (5)	0.060 (6)	0.022 (5)	0.010 (6)	−0.001 (5)
C7B_4	0.093 (6)	0.068 (5)	0.068 (6)	0.023 (5)	0.007 (5)	0.002 (5)
C8B_4	0.092 (6)	0.086 (6)	0.075 (5)	0.021 (6)	0.005 (6)	0.000 (6)
C9B_4	0.092 (6)	0.102 (8)	0.104 (7)	0.027 (7)	0.002 (6)	0.004 (7)
C10B_4	0.096 (7)	0.110 (9)	0.118 (8)	0.021 (8)	0.000 (7)	0.007 (8)
C11B_4	0.117 (14)	0.121 (14)	0.148 (17)	0.020 (12)	−0.020 (13)	0.009 (13)
N1_5	0.043 (3)	0.061 (3)	0.037 (2)	−0.001 (2)	0.007 (2)	0.005 (2)
N2_5	0.048 (3)	0.051 (2)	0.039 (3)	−0.003 (2)	0.011 (2)	0.004 (2)
C1_5	0.050 (4)	0.080 (4)	0.045 (3)	−0.013 (3)	0.002 (3)	0.002 (3)
C2_5	0.066 (4)	0.069 (4)	0.053 (4)	−0.020 (3)	0.010 (3)	−0.009 (3)
C3_5	0.059 (4)	0.059 (3)	0.058 (4)	−0.017 (3)	0.020 (3)	0.005 (3)
C4_5	0.093 (5)	0.097 (4)	0.069 (4)	−0.045 (4)	0.021 (4)	−0.019 (3)
C5_5	0.102 (6)	0.108 (6)	0.068 (6)	−0.042 (5)	0.013 (5)	−0.021 (5)
C6_5	0.115 (7)	0.125 (7)	0.078 (7)	−0.040 (6)	0.003 (6)	−0.023 (6)
C7_5	0.121 (7)	0.147 (8)	0.088 (7)	−0.037 (7)	0.004 (6)	−0.025 (6)
C8_5	0.137 (8)	0.175 (8)	0.095 (8)	−0.033 (7)	0.003 (7)	−0.036 (8)
C9_5	0.153 (10)	0.190 (10)	0.116 (9)	−0.022 (9)	−0.006 (8)	−0.036 (9)
C10_5	0.160 (11)	0.192 (11)	0.112 (10)	−0.030 (10)	−0.010 (9)	−0.031 (10)
C11_5	0.187 (18)	0.201 (17)	0.087 (14)	−0.017 (16)	−0.022 (13)	−0.021 (14)
C4B_5	0.093 (5)	0.097 (4)	0.069 (4)	−0.045 (4)	0.021 (4)	−0.019 (3)
C5B_5	0.107 (7)	0.110 (7)	0.082 (7)	−0.043 (6)	0.018 (6)	−0.028 (6)
C6B_5	0.118 (7)	0.124 (7)	0.086 (7)	−0.037 (6)	0.004 (6)	−0.024 (6)
C7B_5	0.115 (7)	0.127 (8)	0.087 (7)	−0.038 (7)	0.005 (6)	−0.018 (7)
C8B_5	0.128 (7)	0.152 (8)	0.094 (8)	−0.030 (7)	0.000 (7)	−0.033 (7)
C9B_5	0.131 (9)	0.170 (9)	0.085 (9)	−0.023 (8)	−0.007 (8)	−0.041 (8)
C10B_5	0.138 (9)	0.190 (9)	0.101 (9)	−0.024 (8)	0.002 (8)	−0.053 (8)
C11B_5	0.165 (16)	0.188 (15)	0.139 (16)	−0.029 (14)	−0.026 (14)	−0.040 (14)
N1_6	0.040 (2)	0.055 (3)	0.039 (2)	0.0123 (19)	0.004 (2)	0.009 (2)
N2_6	0.046 (2)	0.056 (3)	0.037 (2)	0.021 (2)	0.0098 (19)	0.0143 (19)
C1_6	0.044 (3)	0.077 (4)	0.040 (3)	0.016 (3)	−0.005 (3)	0.008 (3)
C2_6	0.047 (3)	0.090 (4)	0.047 (3)	0.025 (3)	0.005 (3)	0.023 (3)
C3_6	0.049 (3)	0.074 (4)	0.044 (3)	0.031 (3)	0.011 (3)	0.021 (3)
C4_6	0.060 (4)	0.120 (6)	0.060 (4)	0.039 (4)	−0.005 (3)	0.028 (4)
C5_6	0.085 (6)	0.193 (11)	0.054 (5)	0.062 (7)	0.010 (4)	0.052 (6)
C6_6	0.080 (6)	0.144 (8)	0.081 (6)	0.023 (6)	0.006 (5)	0.066 (6)
C7_6	0.089 (7)	0.160 (9)	0.096 (7)	0.044 (7)	0.026 (5)	0.064 (7)
C8_6	0.127 (10)	0.151 (10)	0.113 (8)	−0.005 (8)	0.022 (7)	0.051 (8)
C9_6	0.182 (15)	0.188 (15)	0.185 (15)	0.061 (13)	0.086 (12)	0.066 (12)
C10_6	0.29 (2)	0.163 (14)	0.167 (14)	0.036 (16)	0.083 (15)	0.030 (12)
C11_6	0.40 (3)	0.170 (14)	0.23 (2)	0.149 (19)	0.13 (2)	0.066 (14)
N1_7	0.030 (2)	0.0346 (19)	0.034 (2)	0.0030 (15)	0.0005 (16)	0.0069 (16)
N2_7	0.037 (2)	0.0295 (18)	0.030 (2)	0.0069 (15)	0.0048 (16)	0.0054 (15)
C1_7	0.036 (3)	0.031 (2)	0.038 (3)	0.0010 (19)	0.001 (2)	−0.002 (2)
C2_7	0.048 (3)	0.032 (2)	0.035 (3)	0.001 (2)	−0.003 (2)	0.000 (2)
C3_7	0.046 (3)	0.031 (2)	0.033 (3)	0.013 (2)	0.004 (2)	−0.0011 (19)
C4_7	0.061 (4)	0.032 (2)	0.057 (4)	0.004 (2)	−0.004 (3)	−0.009 (2)
C5_7	0.088 (4)	0.032 (2)	0.066 (4)	0.001 (3)	0.015 (3)	−0.005 (2)
C6_7	0.100 (6)	0.033 (5)	0.076 (6)	−0.001 (5)	0.016 (6)	−0.003 (5)
C7_7	0.116 (8)	0.038 (5)	0.082 (6)	0.003 (6)	0.019 (6)	0.000 (5)
C8_7	0.133 (9)	0.041 (5)	0.097 (7)	0.007 (6)	0.014 (7)	0.006 (5)
C9_7	0.144 (9)	0.049 (5)	0.106 (8)	0.009 (7)	0.016 (7)	0.005 (6)
C10_7	0.159 (11)	0.063 (6)	0.116 (9)	0.005 (8)	0.011 (9)	0.008 (7)
C11_7	0.198 (15)	0.083 (10)	0.121 (13)	0.000 (11)	0.018 (12)	0.036 (10)
C5B_7	0.088 (4)	0.032 (2)	0.066 (4)	0.001 (3)	0.015 (3)	−0.005 (2)
C6B_7	0.100 (6)	0.035 (4)	0.077 (6)	−0.001 (5)	0.017 (5)	−0.009 (4)
C7B_7	0.117 (7)	0.037 (4)	0.083 (6)	0.007 (5)	0.022 (6)	−0.002 (4)
C8B_7	0.136 (8)	0.042 (4)	0.099 (7)	0.006 (6)	0.014 (7)	0.005 (5)
C9B_7	0.151 (9)	0.048 (5)	0.106 (8)	0.007 (6)	0.016 (7)	0.007 (5)
C10B_7	0.159 (10)	0.061 (6)	0.116 (9)	0.009 (7)	0.010 (8)	0.007 (6)
C11B_7	0.151 (12)	0.076 (9)	0.122 (11)	0.021 (9)	0.037 (11)	−0.002 (8)
N1_8	0.030 (2)	0.0331 (18)	0.0256 (19)	0.0106 (15)	0.0057 (15)	0.0024 (15)
N2_8	0.039 (2)	0.0319 (19)	0.032 (2)	0.0050 (16)	0.0089 (17)	0.0050 (15)
C1_8	0.039 (3)	0.035 (2)	0.034 (3)	0.0106 (19)	0.007 (2)	0.0038 (19)
C2_8	0.034 (3)	0.043 (2)	0.029 (2)	0.007 (2)	0.0068 (19)	0.0041 (19)
C3_8	0.043 (3)	0.040 (2)	0.035 (3)	0.003 (2)	0.011 (2)	0.010 (2)
C4_8	0.053 (3)	0.052 (3)	0.037 (3)	0.013 (3)	0.016 (2)	0.005 (2)
C5_8	0.052 (3)	0.056 (3)	0.038 (3)	0.016 (3)	0.012 (2)	0.009 (2)
C6_8	0.062 (4)	0.058 (3)	0.044 (3)	0.015 (3)	0.014 (3)	0.003 (3)
C7_8	0.075 (4)	0.057 (3)	0.055 (4)	0.017 (3)	0.012 (3)	−0.001 (3)
C8_8	0.078 (5)	0.066 (4)	0.071 (5)	0.006 (3)	0.007 (4)	−0.002 (3)
C9_8	0.129 (7)	0.057 (4)	0.079 (5)	0.019 (4)	0.011 (5)	−0.001 (4)
C10_8	0.135 (8)	0.055 (4)	0.120 (7)	0.003 (5)	0.006 (6)	−0.005 (4)
C11_8	0.202 (13)	0.062 (5)	0.136 (9)	0.013 (6)	0.025 (9)	0.004 (5)
N1_9	0.029 (2)	0.0351 (19)	0.034 (2)	0.0043 (15)	0.0024 (16)	0.0066 (16)
N2_9	0.032 (2)	0.0310 (18)	0.030 (2)	0.0047 (15)	0.0026 (16)	0.0042 (15)
C1_9	0.031 (2)	0.040 (2)	0.038 (3)	0.0005 (19)	−0.004 (2)	0.002 (2)
C2_9	0.030 (2)	0.048 (3)	0.032 (3)	0.006 (2)	−0.0002 (19)	0.003 (2)
C3_9	0.039 (3)	0.047 (3)	0.032 (3)	0.012 (2)	−0.001 (2)	0.011 (2)
C4_9	0.042 (3)	0.063 (3)	0.037 (3)	0.008 (2)	−0.007 (2)	0.000 (2)
C5_9	0.044 (3)	0.078 (4)	0.042 (3)	0.004 (3)	−0.012 (3)	−0.004 (3)
C6_9	0.051 (4)	0.086 (4)	0.045 (3)	0.008 (3)	0.005 (3)	−0.010 (3)
C7_9	0.055 (4)	0.082 (4)	0.054 (4)	0.016 (3)	−0.001 (3)	−0.006 (3)
C8_9	0.073 (5)	0.080 (5)	0.068 (4)	0.020 (4)	0.000 (4)	−0.003 (4)
C9_9	0.078 (5)	0.084 (5)	0.072 (5)	0.021 (4)	0.000 (4)	0.001 (4)
C10_9	0.113 (7)	0.086 (6)	0.106 (7)	0.021 (5)	0.015 (6)	0.009 (5)
C11_9	0.147 (10)	0.088 (6)	0.167 (11)	0.010 (7)	0.010 (9)	0.020 (7)
N1_10	0.044 (2)	0.035 (2)	0.030 (2)	0.0065 (17)	0.0111 (18)	0.0061 (17)
N2_10	0.042 (2)	0.037 (2)	0.029 (2)	0.0074 (17)	0.0059 (17)	0.0089 (16)
C1_10	0.053 (3)	0.043 (3)	0.035 (3)	0.005 (2)	0.016 (2)	0.010 (2)
C2_10	0.071 (4)	0.032 (2)	0.030 (3)	0.008 (2)	0.010 (2)	0.009 (2)
C3_10	0.055 (3)	0.044 (3)	0.035 (3)	0.014 (2)	0.007 (2)	0.014 (2)
C4_10	0.103 (5)	0.042 (3)	0.047 (3)	0.007 (3)	0.013 (3)	0.018 (2)
C5_10	0.118 (8)	0.046 (4)	0.063 (5)	0.002 (5)	0.006 (6)	0.019 (4)
C6_10	0.132 (9)	0.053 (5)	0.089 (6)	0.002 (6)	0.004 (7)	0.019 (5)
C7_10	0.145 (9)	0.058 (5)	0.119 (7)	−0.003 (7)	0.002 (8)	0.019 (5)
C8_10	0.161 (10)	0.069 (6)	0.149 (8)	−0.006 (8)	0.005 (9)	0.031 (6)
C9_10	0.177 (11)	0.100 (7)	0.175 (9)	−0.011 (9)	−0.001 (9)	0.021 (7)
C10_10	0.189 (12)	0.128 (8)	0.186 (10)	−0.010 (10)	−0.003 (10)	0.021 (9)
C11_10	0.185 (17)	0.125 (12)	0.190 (15)	0.020 (14)	0.022 (15)	0.016 (13)
C4B_10	0.103 (5)	0.042 (3)	0.047 (3)	0.007 (3)	0.013 (3)	0.018 (2)
C5B_10	0.119 (8)	0.041 (4)	0.063 (5)	0.005 (5)	0.006 (6)	0.019 (4)
C6B_10	0.135 (9)	0.048 (5)	0.086 (6)	0.000 (6)	0.003 (7)	0.025 (5)
C7B_10	0.153 (9)	0.057 (5)	0.117 (7)	−0.002 (7)	−0.004 (8)	0.025 (5)
C8B_10	0.167 (10)	0.077 (6)	0.147 (8)	−0.010 (8)	−0.006 (9)	0.033 (6)
C9B_10	0.185 (11)	0.102 (7)	0.174 (9)	−0.009 (10)	−0.003 (10)	0.028 (8)
C10B_10	0.189 (12)	0.121 (8)	0.179 (10)	−0.006 (10)	0.000 (10)	0.028 (8)
C11B_10	0.187 (17)	0.139 (13)	0.179 (15)	−0.034 (15)	0.012 (16)	0.007 (13)
N1_11	0.040 (2)	0.036 (2)	0.037 (2)	0.0036 (17)	0.0145 (18)	0.0031 (17)
N2_11	0.034 (2)	0.036 (2)	0.036 (2)	0.0018 (16)	0.0015 (17)	0.0117 (17)
C1_11	0.041 (3)	0.049 (3)	0.040 (3)	−0.002 (2)	0.016 (2)	0.008 (2)
C2_11	0.029 (2)	0.036 (2)	0.049 (3)	−0.0006 (19)	0.006 (2)	0.012 (2)
C3_11	0.038 (3)	0.043 (3)	0.041 (3)	−0.011 (2)	−0.003 (2)	0.009 (2)
C4_11	0.037 (3)	0.046 (3)	0.055 (3)	−0.003 (2)	0.010 (2)	0.014 (2)
C5_11	0.044 (3)	0.047 (3)	0.058 (3)	−0.001 (2)	0.014 (3)	0.009 (3)
C6_11	0.046 (3)	0.045 (3)	0.061 (4)	−0.002 (2)	0.015 (3)	0.010 (3)
C7_11	0.059 (4)	0.046 (3)	0.067 (4)	0.003 (3)	0.005 (3)	0.015 (3)
C8_11	0.064 (4)	0.047 (3)	0.072 (4)	−0.005 (3)	0.011 (3)	0.018 (3)
C9_11	0.088 (5)	0.054 (3)	0.061 (4)	−0.001 (3)	0.001 (4)	0.015 (3)
C10_11	0.103 (6)	0.061 (4)	0.082 (5)	−0.025 (4)	0.000 (4)	0.026 (4)
C11_11	0.178 (10)	0.055 (4)	0.100 (6)	−0.020 (5)	−0.010 (6)	0.033 (4)
N1_12	0.040 (2)	0.036 (2)	0.028 (2)	0.0093 (17)	0.0043 (17)	0.0047 (16)
N2_12	0.034 (2)	0.0326 (19)	0.033 (2)	0.0080 (15)	0.0045 (17)	0.0063 (16)
C1_12	0.052 (3)	0.036 (2)	0.032 (3)	0.019 (2)	0.002 (2)	0.0089 (19)
C2_12	0.033 (2)	0.030 (2)	0.043 (3)	0.0055 (18)	0.005 (2)	0.0068 (19)
C3_12	0.043 (3)	0.030 (2)	0.035 (3)	0.0158 (19)	0.000 (2)	0.0007 (18)
C4_12	0.037 (3)	0.031 (2)	0.044 (3)	0.0077 (19)	0.002 (2)	0.005 (2)
C5_12	0.041 (3)	0.034 (2)	0.059 (3)	0.006 (2)	0.008 (2)	0.012 (2)
C6_12	0.041 (3)	0.036 (2)	0.069 (4)	0.009 (2)	0.011 (3)	0.014 (2)
C7_12	0.040 (3)	0.032 (2)	0.090 (4)	0.006 (2)	0.009 (3)	0.014 (3)
C8_12	0.045 (3)	0.034 (3)	0.085 (4)	0.006 (2)	0.009 (3)	0.014 (3)
C9_12	0.044 (3)	0.031 (2)	0.077 (4)	0.005 (2)	0.012 (3)	0.013 (2)
C10_12	0.051 (3)	0.034 (3)	0.073 (4)	0.005 (2)	0.012 (3)	0.017 (2)
C11_12	0.065 (4)	0.038 (3)	0.082 (4)	0.005 (3)	0.007 (3)	0.022 (3)
N1_13	0.050 (3)	0.050 (2)	0.039 (2)	0.008 (2)	0.002 (2)	0.012 (2)
C1_13	0.054 (3)	0.055 (3)	0.054 (3)	0.011 (3)	0.003 (3)	0.015 (3)
C2_13	0.068 (4)	0.091 (5)	0.056 (4)	0.032 (4)	−0.002 (3)	0.015 (3)
C3_13	0.099 (6)	0.090 (5)	0.063 (4)	0.049 (4)	0.012 (4)	0.016 (4)
C4_13	0.132 (9)	0.157 (9)	0.089 (6)	0.092 (8)	0.019 (6)	0.024 (6)
C5_13	0.063 (4)	0.054 (3)	0.055 (4)	0.004 (3)	0.006 (3)	0.013 (3)
C6_13	0.052 (4)	0.078 (4)	0.053 (4)	0.005 (3)	−0.004 (3)	0.016 (3)
C7_13	0.075 (5)	0.077 (4)	0.082 (5)	−0.002 (4)	0.005 (4)	0.023 (4)
C8_13	0.062 (5)	0.120 (7)	0.099 (6)	−0.007 (4)	−0.018 (4)	0.036 (5)
C9_13	0.052 (3)	0.055 (3)	0.049 (3)	0.013 (3)	0.006 (3)	0.013 (3)
C10_13	0.071 (4)	0.065 (4)	0.046 (3)	0.020 (3)	0.016 (3)	0.024 (3)
C11_13	0.133 (7)	0.088 (5)	0.049 (4)	0.049 (5)	0.028 (4)	0.021 (4)
C12_13	0.162 (9)	0.083 (5)	0.068 (5)	0.046 (6)	0.031 (6)	0.014 (4)
C13_13	0.052 (3)	0.062 (3)	0.043 (3)	0.010 (3)	0.002 (3)	0.011 (3)
C14_13	0.049 (3)	0.064 (3)	0.044 (3)	0.012 (3)	0.012 (3)	0.010 (3)
C15_13	0.060 (4)	0.064 (3)	0.038 (3)	−0.002 (3)	0.007 (3)	0.013 (3)
C16_13	0.089 (5)	0.058 (4)	0.053 (4)	0.014 (3)	−0.003 (3)	0.016 (3)
N1_14	0.042 (4)	0.052 (4)	0.049 (4)	0.012 (4)	0.005 (4)	0.010 (3)
C1_14	0.044 (5)	0.053 (4)	0.050 (5)	0.009 (4)	0.009 (4)	0.006 (4)
C2_14	0.042 (5)	0.057 (5)	0.055 (5)	0.013 (4)	0.008 (4)	0.004 (4)
C3_14	0.046 (5)	0.059 (5)	0.046 (5)	0.020 (5)	0.008 (5)	0.005 (4)
C4_14	0.055 (6)	0.056 (5)	0.055 (6)	0.014 (5)	0.006 (5)	0.008 (5)
C5_14	0.040 (4)	0.053 (4)	0.051 (4)	0.011 (4)	0.005 (4)	0.005 (4)
C6_14	0.049 (5)	0.057 (5)	0.050 (5)	0.020 (4)	0.001 (4)	0.000 (4)
C7_14	0.049 (6)	0.055 (6)	0.053 (6)	0.014 (5)	0.007 (5)	−0.001 (5)
C8_14	0.053 (7)	0.062 (7)	0.063 (7)	0.011 (6)	0.011 (6)	0.007 (6)
C9_14	0.043 (4)	0.055 (4)	0.049 (4)	0.009 (4)	0.009 (4)	0.009 (4)
C10_14	0.045 (5)	0.063 (5)	0.049 (5)	0.002 (4)	0.012 (4)	0.007 (4)
C11_14	0.049 (6)	0.062 (6)	0.055 (6)	0.008 (5)	0.009 (5)	0.010 (5)
C12_14	0.046 (6)	0.063 (7)	0.048 (6)	0.003 (5)	0.005 (5)	0.008 (5)
C13_14	0.042 (5)	0.054 (5)	0.048 (5)	0.008 (4)	0.003 (4)	0.009 (4)
C14_14	0.050 (6)	0.052 (5)	0.053 (5)	0.015 (5)	−0.001 (5)	0.008 (4)
C15_14	0.057 (6)	0.059 (6)	0.062 (6)	0.002 (5)	−0.009 (5)	0.009 (5)
C16_14	0.058 (6)	0.055 (6)	0.061 (6)	0.002 (5)	0.000 (6)	0.012 (5)
N1_15	0.058 (4)	0.102 (5)	0.091 (5)	0.020 (4)	0.011 (4)	0.017 (5)
C1_15	0.057 (4)	0.102 (6)	0.091 (5)	0.023 (5)	0.012 (4)	0.013 (5)
C2_15	0.050 (5)	0.102 (6)	0.084 (6)	0.026 (5)	0.011 (5)	0.013 (6)
C3_15	0.053 (6)	0.101 (7)	0.085 (7)	0.026 (6)	0.008 (6)	0.015 (6)
C4_15	0.053 (7)	0.104 (8)	0.084 (7)	0.026 (7)	0.010 (7)	0.012 (7)
C5_15	0.059 (5)	0.100 (6)	0.092 (5)	0.021 (5)	0.012 (5)	0.015 (5)
C6_15	0.058 (5)	0.097 (6)	0.088 (6)	0.022 (5)	0.010 (5)	0.018 (6)
C7_15	0.057 (6)	0.096 (7)	0.085 (7)	0.022 (6)	0.013 (6)	0.018 (6)
C8_15	0.059 (7)	0.094 (7)	0.084 (7)	0.023 (7)	0.009 (7)	0.018 (7)
C9_15	0.059 (5)	0.102 (6)	0.090 (5)	0.019 (5)	0.012 (4)	0.019 (5)
C10_15	0.059 (5)	0.103 (6)	0.090 (5)	0.021 (5)	0.010 (5)	0.018 (5)
C11_15	0.060 (6)	0.102 (7)	0.086 (6)	0.020 (6)	0.010 (6)	0.023 (6)
C12_15	0.059 (7)	0.102 (8)	0.085 (7)	0.022 (7)	0.007 (7)	0.022 (7)
C13_15	0.062 (5)	0.105 (6)	0.094 (5)	0.020 (5)	0.012 (4)	0.016 (5)
C14_15	0.065 (5)	0.105 (6)	0.096 (6)	0.020 (5)	0.012 (5)	0.018 (5)
C15_15	0.065 (6)	0.108 (7)	0.097 (7)	0.019 (6)	0.014 (6)	0.017 (7)
C16_15	0.067 (7)	0.109 (8)	0.100 (8)	0.020 (7)	0.012 (7)	0.017 (8)
N1B_15	0.058 (4)	0.101 (5)	0.091 (5)	0.021 (4)	0.011 (4)	0.018 (5)
C1B_15	0.062 (5)	0.106 (6)	0.095 (5)	0.020 (5)	0.010 (4)	0.015 (5)
C2B_15	0.065 (5)	0.106 (6)	0.095 (6)	0.018 (5)	0.009 (5)	0.018 (5)
C3B_15	0.066 (6)	0.106 (7)	0.098 (7)	0.017 (6)	0.008 (6)	0.020 (7)
C4B_15	0.063 (7)	0.104 (8)	0.099 (8)	0.018 (7)	0.010 (7)	0.022 (7)
C5B_15	0.059 (5)	0.101 (6)	0.092 (5)	0.022 (5)	0.012 (5)	0.016 (5)
C6B_15	0.060 (6)	0.097 (6)	0.088 (6)	0.018 (6)	0.012 (5)	0.018 (6)
C7B_15	0.058 (6)	0.096 (7)	0.085 (7)	0.019 (6)	0.012 (6)	0.018 (6)
C8B_15	0.062 (7)	0.098 (7)	0.086 (7)	0.017 (7)	0.009 (7)	0.018 (7)
C9B_15	0.061 (4)	0.102 (5)	0.091 (5)	0.021 (4)	0.010 (4)	0.017 (5)
C10B_15	0.060 (5)	0.103 (6)	0.090 (6)	0.019 (5)	0.011 (5)	0.019 (5)
C11B_15	0.059 (6)	0.103 (7)	0.086 (6)	0.021 (6)	0.009 (6)	0.024 (6)
C12B_15	0.059 (7)	0.102 (8)	0.086 (7)	0.023 (7)	0.007 (7)	0.023 (7)
C13B_15	0.056 (5)	0.102 (6)	0.090 (5)	0.022 (5)	0.011 (4)	0.014 (5)
C14B_15	0.051 (5)	0.100 (6)	0.083 (6)	0.022 (5)	0.014 (5)	0.017 (6)
C15B_15	0.051 (6)	0.101 (7)	0.083 (7)	0.024 (6)	0.014 (6)	0.014 (6)
C16B_15	0.052 (7)	0.101 (8)	0.082 (7)	0.029 (7)	0.009 (7)	0.015 (7)

### Bis(tetrabutylazanium) hexa-µ4-hydroxido-dodeca-µ2-(4-octylpyrazolato)-hexahedro-octanickel (3) Geometric parameters (Å, º)

**Table:**

Ni1_16—N1_1	2.015 (4)	C1_8—H1_8	0.9500
Ni1_16—N2_2	2.026 (4)	C2_8—C3_8	1.383 (6)
Ni1_16—N2_6	2.026 (4)	C2_8—C4_8	1.509 (6)
Ni1_16—O2_16	2.165 (3)	C3_8—H3_8	0.9500
Ni1_16—O3_16^i^	2.169 (3)	C4_8—C5_8	1.517 (7)
Ni1_16—O1_16	2.197 (3)	C4_8—H4A_8	0.9900
Ni1_16—Ni2_16	2.975 (2)	C4_8—H4B_8	0.9900
Ni1_16—Ni3_16^i^	2.9842 (16)	C5_8—C6_8	1.509 (7)
Ni1_16—Ni4_16	2.9876 (17)	C5_8—H5A_8	0.9900
Ni2_16—N1_2	2.020 (4)	C5_8—H5B_8	0.9900
Ni2_16—N2_3	2.024 (4)	C6_8—C7_8	1.518 (7)
Ni2_16—N2_5^i^	2.026 (5)	C6_8—H6A_8	0.9900
Ni2_16—O3_16^i^	2.154 (3)	C6_8—H6B_8	0.9900
Ni2_16—O1_16	2.174 (3)	C7_8—C8_8	1.495 (8)
Ni2_16—O2_16^i^	2.192 (3)	C7_8—H7A_8	0.9900
Ni2_16—Ni4_16^i^	2.9887 (16)	C7_8—H7B_8	0.9900
Ni3_16—N1_3	2.022 (4)	C8_8—C9_8	1.522 (8)
Ni3_16—N1_4	2.028 (4)	C8_8—H8A_8	0.9900
Ni3_16—N2_1^i^	2.042 (4)	C8_8—H8B_8	0.9900
Ni3_16—O2_16^i^	2.158 (3)	C9_8—C10_8	1.501 (10)
Ni3_16—O1_16	2.173 (3)	C9_8—H9A_8	0.9900
Ni3_16—O3_16	2.191 (3)	C9_8—H9B_8	0.9900
Ni3_16—Ni4_16	2.983 (2)	C10_8—C11_8	1.523 (10)
Ni4_16—N1_5	2.014 (4)	C10_8—H10A_8	0.9900
Ni4_16—N2_4	2.023 (4)	C10_8—H10B_8	0.9900
Ni4_16—N1_6	2.025 (4)	C11_8—H11A_8	0.9800
Ni4_16—O1_16	2.157 (3)	C11_8—H11B_8	0.9800
Ni4_16—O3_16	2.168 (3)	C11_8—H11C_8	0.9800
Ni4_16—O2_16	2.196 (3)	N1_9—C1_9	1.333 (5)
Ni5_16—N2_7	2.018 (4)	N1_9—N2_9	1.356 (5)
Ni5_16—N2_12^ii^	2.019 (4)	N2_9—C3_9	1.343 (6)
Ni5_16—N1_8	2.042 (4)	C1_9—C2_9	1.393 (6)
Ni5_16—O6_16	2.156 (3)	C1_9—H1_9	0.9500
Ni5_16—O5_16^ii^	2.183 (3)	C2_9—C3_9	1.384 (6)
Ni5_16—O4_16	2.187 (3)	C2_9—C4_9	1.508 (6)
Ni5_16—Ni8_16	2.9859 (19)	C3_9—H3_9	0.9500
Ni5_16—Ni6_16	2.9899 (16)	C4_9—C5_9	1.544 (7)
Ni6_16—N2_8	2.018 (4)	C4_9—H4A_9	0.9900
Ni6_16—N1_11	2.023 (4)	C4_9—H4B_9	0.9900
Ni6_16—N1_10	2.035 (4)	C5_9—C6_9	1.512 (8)
Ni6_16—O6_16^ii^	2.173 (3)	C5_9—H5A_9	0.9900
Ni6_16—O5_16^ii^	2.177 (3)	C5_9—H5B_9	0.9900
Ni6_16—O4_16	2.180 (3)	C6_9—C7_9	1.508 (8)
Ni6_16—Ni8_16^ii^	2.9772 (17)	C6_9—H6A_9	0.9900
Ni6_16—Ni7_16	2.9910 (19)	C6_9—H6B_9	0.9900
Ni7_16—N1_12	2.027 (4)	C7_9—C8_9	1.504 (8)
Ni7_16—N2_9	2.031 (4)	C7_9—H7A_9	0.9900
Ni7_16—N2_10	2.031 (4)	C7_9—H7B_9	0.9900
Ni7_16—O4_16	2.159 (3)	C8_9—C9_9	1.513 (9)
Ni7_16—O5_16	2.178 (3)	C8_9—H8A_9	0.9900
Ni7_16—O6_16^ii^	2.184 (3)	C8_9—H8B_9	0.9900
Ni7_16—Ni8_16	2.9929 (16)	C9_9—C10_9	1.513 (9)
Ni8_16—N2_11^ii^	2.012 (4)	C9_9—H9A_9	0.9900
Ni8_16—N1_7	2.017 (4)	C9_9—H9B_9	0.9900
Ni8_16—N1_9	2.037 (4)	C10_9—C11_9	1.497 (11)
Ni8_16—O6_16	2.172 (3)	C10_9—H10A_9	0.9900
Ni8_16—O4_16	2.178 (3)	C10_9—H10B_9	0.9900
Ni8_16—O5_16	2.179 (3)	C11_9—H11A_9	0.9800
O1_16—H1_16	0.824 (16)	C11_9—H11B_9	0.9800
O2_16—H2_16	0.824 (16)	C11_9—H11C_9	0.9800
O3_16—H3_16	0.826 (16)	N1_10—C1_10	1.341 (6)
O4_16—H4_16	0.827 (16)	N1_10—N2_10	1.350 (5)
O5_16—H5_16	0.811 (16)	N2_10—C3_10	1.340 (6)
O6_16—H6_16	0.833 (16)	C1_10—C2_10	1.379 (7)
N1_1—C1_1	1.335 (6)	C1_10—H1_10	0.9500
N1_1—N2_1	1.344 (6)	C2_10—C3_10	1.382 (7)
N2_1—C3_1	1.339 (6)	C2_10—C4B_10	1.512 (6)
C1_1—C2_1	1.375 (7)	C2_10—C4_10	1.512 (6)
C1_1—H1_1	0.9500	C3_10—H3_10	0.9500
C2_1—C3_1	1.372 (7)	C4_10—C5_10	1.527 (13)
C2_1—C4_1	1.521 (7)	C4_10—H4A_10	0.9900
C3_1—H3_1	0.9500	C4_10—H4B_10	0.9900
C4_1—C5_1	1.494 (8)	C5_10—C6_10	1.521 (14)
C4_1—H4A_1	0.9900	C5_10—H5A_10	0.9900
C4_1—H4B_1	0.9900	C5_10—H5B_10	0.9900
C5_1—C6_1	1.513 (8)	C6_10—C7_10	1.495 (14)
C5_1—H5A_1	0.9900	C6_10—H6A_10	0.9900
C5_1—H5B_1	0.9900	C6_10—H6B_10	0.9900
C6_1—C7_1	1.518 (10)	C7_10—C8_10	1.515 (15)
C6_1—H6A_1	0.9900	C7_10—H7A_10	0.9900
C6_1—H6B_1	0.9900	C7_10—H7B_10	0.9900
C7_1—C8_1	1.499 (9)	C8_10—C9_10	1.481 (15)
C7_1—H7A_1	0.9900	C8_10—H8A_10	0.9900
C7_1—H7B_1	0.9900	C8_10—H8B_10	0.9900
C8_1—C9_1	1.500 (11)	C9_10—C10_10	1.566 (17)
C8_1—H8A_1	0.9900	C9_10—H9A_10	0.9900
C8_1—H8B_1	0.9900	C9_10—H9B_10	0.9900
C9_1—C10_1	1.522 (12)	C10_10—C11_10	1.546 (17)
C9_1—H9A_1	0.9900	C10_10—H10A_10	0.9900
C9_1—H9B_1	0.9900	C10_10—H10B_10	0.9900
C10_1—C11_1	1.438 (13)	C11_10—H11A_10	0.9800
C10_1—H10A_1	0.9900	C11_10—H11B_10	0.9800
C10_1—H10B_1	0.9900	C11_10—H11C_10	0.9800
C11_1—H11A_1	0.9800	C4B_10—C5B_10	1.510 (14)
C11_1—H11B_1	0.9800	C4B_10—H4BA_10	0.9900
C11_1—H11C_1	0.9800	C4B_10—H4BB_10	0.9900
N1_2—C1_2	1.338 (6)	C5B_10—C6B_10	1.515 (14)
N1_2—N2_2	1.362 (6)	C5B_10—H5BA_10	0.9900
N2_2—C3_2	1.355 (6)	C5B_10—H5BB_10	0.9900
C1_2—C2_2	1.381 (8)	C6B_10—C7B_10	1.497 (15)
C1_2—H1_2	0.9500	C6B_10—H6BA_10	0.9900
C2_2—C3_2	1.380 (8)	C6B_10—H6BB_10	0.9900
C2_2—C4_2	1.501 (7)	C7B_10—C8B_10	1.491 (15)
C3_2—H3_2	0.9500	C7B_10—H7BA_10	0.9900
C4_2—C5_2	1.471 (8)	C7B_10—H7BB_10	0.9900
C4_2—H4A_2	0.9900	C8B_10—C9B_10	1.494 (16)
C4_2—H4B_2	0.9900	C8B_10—H8BA_10	0.9900
C5_2—C6_2	1.500 (7)	C8B_10—H8BB_10	0.9900
C5_2—H5A_2	0.9900	C9B_10—C10B_10	1.617 (17)
C5_2—H5B_2	0.9900	C9B_10—H9BA_10	0.9900
C6_2—C7_2	1.476 (8)	C9B_10—H9BB_10	0.9900
C6_2—H6A_2	0.9900	C10B_10—C11B_10	1.529 (17)
C6_2—H6B_2	0.9900	C10B_10—H10C_10	0.9900
C7_2—C8_2	1.491 (7)	C10B_10—H10D_10	0.9900
C7_2—H7A_2	0.9900	C11B_10—H11D_10	0.9800
C7_2—H7B_2	0.9900	C11B_10—H11E_10	0.9800
C8_2—C9_2	1.464 (8)	C11B_10—H11F_10	0.9800
C8_2—H8A_2	0.9900	N1_11—C1_11	1.339 (6)
C8_2—H8B_2	0.9900	N1_11—N2_11	1.358 (5)
C9_2—C10_2	1.487 (8)	N2_11—C3_11	1.345 (6)
C9_2—H9A_2	0.9900	C1_11—C2_11	1.379 (6)
C9_2—H9B_2	0.9900	C1_11—H1_11	0.9500
C10_2—C11_2	1.445 (9)	C2_11—C3_11	1.377 (6)
C10_2—H10A_2	0.9900	C2_11—C4_11	1.516 (6)
C10_2—H10B_2	0.9900	C3_11—H3_11	0.9500
C11_2—H11A_2	0.9800	C4_11—C5_11	1.521 (7)
C11_2—H11B_2	0.9800	C4_11—H4A_11	0.9900
C11_2—H11C_2	0.9800	C4_11—H4B_11	0.9900
N1_3—C1_3	1.344 (6)	C5_11—C6_11	1.512 (6)
N1_3—N2_3	1.358 (5)	C5_11—H5A_11	0.9900
N2_3—C3_3	1.332 (6)	C5_11—H5B_11	0.9900
C1_3—C2_3	1.381 (7)	C6_11—C7_11	1.515 (7)
C1_3—H1_3	0.9500	C6_11—H6A_11	0.9900
C2_3—C3_3	1.385 (7)	C6_11—H6B_11	0.9900
C2_3—C4B_3	1.513 (7)	C7_11—C8_11	1.511 (7)
C2_3—C4_3	1.513 (7)	C7_11—H7A_11	0.9900
C3_3—H3_3	0.9500	C7_11—H7B_11	0.9900
C4_3—C5_3	1.491 (10)	C8_11—C9_11	1.517 (8)
C4_3—H4A_3	0.9900	C8_11—H8A_11	0.9900
C4_3—H4B_3	0.9900	C8_11—H8B_11	0.9900
C5_3—C6_3	1.533 (11)	C9_11—C10_11	1.512 (8)
C5_3—H5A_3	0.9900	C9_11—H9A_11	0.9900
C5_3—H5B_3	0.9900	C9_11—H9B_11	0.9900
C6_3—C7_3	1.437 (11)	C10_11—C11_11	1.503 (9)
C6_3—H6A_3	0.9900	C10_11—H10A_11	0.9900
C6_3—H6B_3	0.9900	C10_11—H10B_11	0.9900
C7_3—C8_3	1.495 (13)	C11_11—H11A_11	0.9800
C7_3—H7A_3	0.9900	C11_11—H11B_11	0.9800
C7_3—H7B_3	0.9900	C11_11—H11C_11	0.9800
C8_3—C9_3	1.539 (13)	N1_12—C1_12	1.342 (6)
C8_3—H8A_3	0.9900	N1_12—N2_12	1.361 (5)
C8_3—H8B_3	0.9900	N2_12—C3_12	1.340 (5)
C9_3—C10_3	1.530 (14)	C1_12—C2_12	1.380 (6)
C9_3—H9A_3	0.9900	C1_12—H1_12	0.9500
C9_3—H9B_3	0.9900	C2_12—C3_12	1.384 (6)
C10_3—C11_3	1.476 (14)	C2_12—C4_12	1.502 (6)
C10_3—H10A_3	0.9900	C3_12—H3_12	0.9500
C10_3—H10B_3	0.9900	C4_12—C5_12	1.524 (6)
C11_3—H11A_3	0.9800	C4_12—H4A_12	0.9900
C11_3—H11B_3	0.9800	C4_12—H4B_12	0.9900
C11_3—H11C_3	0.9800	C5_12—C6_12	1.527 (6)
C4B_3—C5B_3	1.474 (16)	C5_12—H5A_12	0.9900
C4B_3—H4BA_3	0.9900	C5_12—H5B_12	0.9900
C4B_3—H4BB_3	0.9900	C6_12—C7_12	1.527 (6)
C5B_3—C6B_3	1.478 (16)	C6_12—H6A_12	0.9900
C5B_3—H5BA_3	0.9900	C6_12—H6B_12	0.9900
C5B_3—H5BB_3	0.9900	C7_12—C8_12	1.520 (6)
C6B_3—C7B_3	1.466 (16)	C7_12—H7A_12	0.9900
C6B_3—H6BA_3	0.9900	C7_12—H7B_12	0.9900
C6B_3—H6BB_3	0.9900	C8_12—C9_12	1.521 (6)
C7B_3—C8B_3	1.508 (17)	C8_12—H8A_12	0.9900
C7B_3—H7BA_3	0.9900	C8_12—H8B_12	0.9900
C7B_3—H7BB_3	0.9900	C9_12—C10_12	1.520 (6)
C8B_3—C9B_3	1.530 (17)	C9_12—H9A_12	0.9900
C8B_3—H8BA_3	0.9900	C9_12—H9B_12	0.9900
C8B_3—H8BB_3	0.9900	C10_12—C11_12	1.509 (7)
C9B_3—C10B_3	1.513 (18)	C10_12—H10A_12	0.9900
C9B_3—H9BA_3	0.9900	C10_12—H10B_12	0.9900
C9B_3—H9BB_3	0.9900	C11_12—H11A_12	0.9800
C10B_3—C11B_3	1.479 (18)	C11_12—H11B_12	0.9800
C10B_3—H10C_3	0.9900	C11_12—H11C_12	0.9800
C10B_3—H10D_3	0.9900	N1_13—C5_13	1.505 (7)
C11B_3—H11D_3	0.9800	N1_13—C9_13	1.516 (6)
C11B_3—H11E_3	0.9800	N1_13—C1_13	1.533 (7)
C11B_3—H11F_3	0.9800	N1_13—C13_13	1.540 (7)
N1_4—C1_4	1.349 (6)	C1_13—C2_13	1.488 (8)
N1_4—N2_4	1.365 (6)	C1_13—H1A_13	0.9900
N2_4—C3_4	1.344 (6)	C1_13—H1B_13	0.9900
C1_4—C2_4	1.384 (7)	C2_13—C3_13	1.508 (9)
C1_4—H1_4	0.9500	C2_13—H2A_13	0.9900
C2_4—C3_4	1.392 (7)	C2_13—H2B_13	0.9900
C2_4—C4B_4	1.511 (7)	C3_13—C4_13	1.462 (9)
C2_4—C4_4	1.511 (7)	C3_13—H3A_13	0.9900
C3_4—H3_4	0.9500	C3_13—H3B_13	0.9900
C4_4—C5_4	1.533 (14)	C4_13—H4A_13	0.9800
C4_4—H4A_4	0.9900	C4_13—H4B_13	0.9800
C4_4—H4B_4	0.9900	C4_13—H4C_13	0.9800
C5_4—C6_4	1.520 (15)	C5_13—C6_13	1.521 (8)
C5_4—H5A_4	0.9900	C5_13—H5A_13	0.9900
C5_4—H5B_4	0.9900	C5_13—H5B_13	0.9900
C6_4—C7_4	1.527 (14)	C6_13—C7_13	1.508 (8)
C6_4—H6A_4	0.9900	C6_13—H6A_13	0.9900
C6_4—H6B_4	0.9900	C6_13—H6B_13	0.9900
C7_4—C8_4	1.520 (14)	C7_13—C8_13	1.511 (10)
C7_4—H7A_4	0.9900	C7_13—H7A_13	0.9900
C7_4—H7B_4	0.9900	C7_13—H7B_13	0.9900
C8_4—C9_4	1.505 (15)	C8_13—H8A_13	0.9800
C8_4—H8A_4	0.9900	C8_13—H8B_13	0.9800
C8_4—H8B_4	0.9900	C8_13—H8C_13	0.9800
C9_4—C10_4	1.522 (15)	C9_13—C10_13	1.516 (7)
C9_4—H9A_4	0.9900	C9_13—H9A_13	0.9900
C9_4—H9B_4	0.9900	C9_13—H9B_13	0.9900
C10_4—C11_4	1.519 (16)	C10_13—C11_13	1.495 (8)
C10_4—H10A_4	0.9900	C10_13—H10A_13	0.9900
C10_4—H10B_4	0.9900	C10_13—H10B_13	0.9900
C11_4—H11A_4	0.9800	C11_13—C12_13	1.466 (10)
C11_4—H11B_4	0.9800	C11_13—H11A_13	0.9900
C11_4—H11C_4	0.9800	C11_13—H11B_13	0.9900
C4B_4—C5B_4	1.514 (14)	C12_13—H12A_13	0.9800
C4B_4—H4BA_4	0.9900	C12_13—H12B_13	0.9800
C4B_4—H4BB_4	0.9900	C12_13—H12C_13	0.9800
C5B_4—C6B_4	1.519 (15)	C13_13—C14_13	1.530 (7)
C5B_4—H5BA_4	0.9900	C13_13—H13A_13	0.9900
C5B_4—H5BB_4	0.9900	C13_13—H13B_13	0.9900
C6B_4—C7B_4	1.498 (14)	C14_13—C15_13	1.490 (8)
C6B_4—H6BA_4	0.9900	C14_13—H14A_13	0.9900
C6B_4—H6BB_4	0.9900	C14_13—H14B_13	0.9900
C7B_4—C8B_4	1.500 (14)	C15_13—C16_13	1.518 (8)
C7B_4—H7BA_4	0.9900	C15_13—H15A_13	0.9900
C7B_4—H7BB_4	0.9900	C15_13—H15B_13	0.9900
C8B_4—C9B_4	1.525 (15)	C16_13—H16A_13	0.9800
C8B_4—H8BA_4	0.9900	C16_13—H16B_13	0.9800
C8B_4—H8BB_4	0.9900	C16_13—H16C_13	0.9800
C9B_4—C10B_4	1.522 (15)	N1_14—C5_14	1.518 (14)
C9B_4—H9BA_4	0.9900	N1_14—C13_14	1.521 (9)
C9B_4—H9BB_4	0.9900	N1_14—C9_14	1.521 (13)
C10B_4—C11B_4	1.513 (16)	N1_14—C1_14	1.524 (13)
C10B_4—H10C_4	0.9900	C1_14—C2_14	1.512 (15)
C10B_4—H10D_4	0.9900	C1_14—H1A_14	0.9900
C11B_4—H11D_4	0.9800	C1_14—H1B_14	0.9900
C11B_4—H11E_4	0.9800	C2_14—C3_14	1.505 (13)
C11B_4—H11F_4	0.9800	C2_14—H2A_14	0.9900
N1_5—C1_5	1.346 (7)	C2_14—H2B_14	0.9900
N1_5—N2_5	1.357 (6)	C3_14—C4_14	1.502 (14)
N2_5—C3_5	1.342 (6)	C3_14—H3A_14	0.9900
C1_5—C2_5	1.373 (8)	C3_14—H3B_14	0.9900
C1_5—H1_5	0.9500	C4_14—H4A_14	0.9800
C2_5—C3_5	1.400 (8)	C4_14—H4B_14	0.9800
C2_5—C4B_5	1.516 (8)	C4_14—H4C_14	0.9800
C2_5—C4_5	1.516 (8)	C5_14—C6_14	1.507 (13)
C3_5—H3_5	0.9500	C5_14—H5A_14	0.9900
C4_5—C5_5	1.495 (13)	C5_14—H5B_14	0.9900
C4_5—H4A_5	0.9900	C6_14—C7_14	1.510 (14)
C4_5—H4B_5	0.9900	C6_14—H6A_14	0.9900
C5_5—C6_5	1.507 (15)	C6_14—H6B_14	0.9900
C5_5—H5A_5	0.9900	C7_14—C8_14	1.542 (15)
C5_5—H5B_5	0.9900	C7_14—H7A_14	0.9900
C6_5—C7_5	1.500 (16)	C7_14—H7B_14	0.9900
C6_5—H6A_5	0.9900	C8_14—H8A_14	0.9800
C6_5—H6B_5	0.9900	C8_14—H8B_14	0.9800
C7_5—C8_5	1.481 (16)	C8_14—H8C_14	0.9800
C7_5—H7A_5	0.9900	C9_14—C10_14	1.485 (13)
C7_5—H7B_5	0.9900	C9_14—H9A_14	0.9900
C8_5—C9_5	1.434 (17)	C9_14—H9B_14	0.9900
C8_5—H8A_5	0.9900	C10_14—C11_14	1.508 (15)
C8_5—H8B_5	0.9900	C10_14—H10A_14	0.9900
C9_5—C10_5	1.610 (16)	C10_14—H10B_14	0.9900
C9_5—H9A_5	0.9900	C11_14—C12_14	1.531 (15)
C9_5—H9B_5	0.9900	C11_14—H11A_14	0.9900
C10_5—C11_5	1.487 (17)	C11_14—H11B_14	0.9900
C10_5—H10A_5	0.9900	C12_14—H12A_14	0.9800
C10_5—H10B_5	0.9900	C12_14—H12B_14	0.9800
C11_5—H11A_5	0.9800	C12_14—H12C_14	0.9800
C11_5—H11B_5	0.9800	C13_14—C14_14	1.544 (16)
C11_5—H11C_5	0.9800	C13_14—H13A_14	0.9900
C4B_5—C5B_5	1.554 (15)	C13_14—H13B_14	0.9900
C4B_5—H4BA_5	0.9900	C14_14—C15_14	1.473 (15)
C4B_5—H4BB_5	0.9900	C14_14—H14A_14	0.9900
C5B_5—C6B_5	1.509 (15)	C14_14—H14B_14	0.9900
C5B_5—H5BA_5	0.9900	C15_14—C16_14	1.527 (15)
C5B_5—H5BB_5	0.9900	C15_14—H15A_14	0.9900
C6B_5—C7B_5	1.508 (17)	C15_14—H15B_14	0.9900
C6B_5—H6BA_5	0.9900	C16_14—H16A_14	0.9800
C6B_5—H6BB_5	0.9900	C16_14—H16B_14	0.9800
C7B_5—C8B_5	1.488 (16)	C16_14—H16C_14	0.9800
C7B_5—H7BA_5	0.9900	N1_15—C5_15	1.510 (16)
C7B_5—H7BB_5	0.9900	N1_15—C13_15	1.522 (16)
C8B_5—C9B_5	1.476 (17)	N1_15—C1_15	1.524 (16)
C8B_5—H8BA_5	0.9900	N1_15—C9_15	1.524 (16)
C8B_5—H8BB_5	0.9900	C1_15—C2_15	1.51 (2)
C9B_5—C10B_5	1.564 (17)	C1_15—H1A_15	0.9900
C9B_5—H9BA_5	0.9900	C1_15—H1B_15	0.9900
C9B_5—H9BB_5	0.9900	C2_15—C3_15	1.48 (2)
C10B_5—C11B_5	1.498 (17)	C2_15—H2A_15	0.9900
C10B_5—H10C_5	0.9900	C2_15—H2B_15	0.9900
C10B_5—H10D_5	0.9900	C3_15—C4_15	1.52 (2)
C11B_5—H11D_5	0.9800	C3_15—H3A_15	0.9900
C11B_5—H11E_5	0.9800	C3_15—H3B_15	0.9900
C11B_5—H11F_5	0.9800	C4_15—H4A_15	0.9800
N1_6—C1_6	1.340 (6)	C4_15—H4B_15	0.9800
N1_6—N2_6	1.350 (6)	C4_15—H4C_15	0.9800
N2_6—C3_6	1.343 (6)	C5_15—C6_15	1.52 (2)
C1_6—C2_6	1.378 (7)	C5_15—H5A_15	0.9900
C1_6—H1_6	0.9500	C5_15—H5B_15	0.9900
C2_6—C3_6	1.379 (8)	C6_15—C7_15	1.51 (2)
C2_6—C4_6	1.509 (7)	C6_15—H6A_15	0.9900
C3_6—H3_6	0.9500	C6_15—H6B_15	0.9900
C4_6—C5_6	1.513 (9)	C7_15—C8_15	1.48 (2)
C4_6—H4A_6	0.9900	C7_15—H7A_15	0.9900
C4_6—H4B_6	0.9900	C7_15—H7B_15	0.9900
C5_6—C6_6	1.533 (10)	C8_15—H8A_15	0.9800
C5_6—H5A_6	0.9900	C8_15—H8B_15	0.9800
C5_6—H5B_6	0.9900	C8_15—H8C_15	0.9800
C6_6—C7_6	1.491 (10)	C9_15—C10_15	1.48 (2)
C6_6—H6A_6	0.9900	C9_15—H9A_15	0.9900
C6_6—H6B_6	0.9900	C9_15—H9B_15	0.9900
C7_6—C8_6	1.501 (11)	C10_15—C11_15	1.48 (2)
C7_6—H7A_6	0.9900	C10_15—H10A_15	0.9900
C7_6—H7B_6	0.9900	C10_15—H10B_15	0.9900
C8_6—C9_6	1.531 (12)	C11_15—C12_15	1.53 (2)
C8_6—H8A_6	0.9900	C11_15—H11A_15	0.9900
C8_6—H8B_6	0.9900	C11_15—H11B_15	0.9900
C9_6—C10_6	1.464 (14)	C12_15—H12A_15	0.9800
C9_6—H9A_6	0.9900	C12_15—H12B_15	0.9800
C9_6—H9B_6	0.9900	C12_15—H12C_15	0.9800
C10_6—C11_6	1.527 (14)	C13_15—C14_15	1.53 (2)
C10_6—H10A_6	0.9900	C13_15—H13A_15	0.9900
C10_6—H10B_6	0.9900	C13_15—H13B_15	0.9900
C11_6—H11A_6	0.9800	C14_15—C15_15	1.46 (2)
C11_6—H11B_6	0.9800	C14_15—H14A_15	0.9900
C11_6—H11C_6	0.9800	C14_15—H14B_15	0.9900
N1_7—C1_7	1.341 (6)	C15_15—C16_15	1.53 (2)
N1_7—N2_7	1.370 (5)	C15_15—H15A_15	0.9900
N2_7—C3_7	1.342 (5)	C15_15—H15B_15	0.9900
C1_7—C2_7	1.389 (6)	C16_15—H16A_15	0.9800
C1_7—H1_7	0.9500	C16_15—H16B_15	0.9800
C2_7—C3_7	1.379 (6)	C16_15—H16C_15	0.9800
C2_7—C4_7	1.503 (6)	N1B_15—C5B_15	1.515 (16)
C3_7—H3_7	0.9500	N1B_15—C1B_15	1.516 (16)
C4_7—C5B_7	1.514 (8)	N1B_15—C13B_15	1.530 (16)
C4_7—C5_7	1.514 (8)	N1B_15—C9B_15	1.536 (16)
C4_7—H4A_7	0.9900	C1B_15—C2B_15	1.51 (2)
C4_7—H4B_7	0.9900	C1B_15—H1C_15	0.9900
C5_7—C6_7	1.539 (15)	C1B_15—H1D_15	0.9900
C5_7—H5A_7	0.9900	C2B_15—C3B_15	1.47 (2)
C5_7—H5B_7	0.9900	C2B_15—H2C_15	0.9900
C6_7—C7_7	1.504 (16)	C2B_15—H2D_15	0.9900
C6_7—H6A_7	0.9900	C3B_15—C4B_15	1.50 (2)
C6_7—H6B_7	0.9900	C3B_15—H3C_15	0.9900
C7_7—C8_7	1.509 (15)	C3B_15—H3D_15	0.9900
C7_7—H7A_7	0.9900	C4B_15—H4D_15	0.9800
C7_7—H7B_7	0.9900	C4B_15—H4E_15	0.9800
C8_7—C9_7	1.515 (15)	C4B_15—H4F_15	0.9800
C8_7—H8A_7	0.9900	C5B_15—C6B_15	1.51 (2)
C8_7—H8B_7	0.9900	C5B_15—H5C_15	0.9900
C9_7—C10_7	1.512 (16)	C5B_15—H5D_15	0.9900
C9_7—H9A_7	0.9900	C6B_15—C7B_15	1.51 (2)
C9_7—H9B_7	0.9900	C6B_15—H6C_15	0.9900
C10_7—C11_7	1.501 (16)	C6B_15—H6D_15	0.9900
C10_7—H10A_7	0.9900	C7B_15—C8B_15	1.49 (2)
C10_7—H10B_7	0.9900	C7B_15—H7C_15	0.9900
C11_7—H11A_7	0.9800	C7B_15—H7D_15	0.9900
C11_7—H11B_7	0.9800	C8B_15—H8D_15	0.9800
C11_7—H11C_7	0.9800	C8B_15—H8E_15	0.9800
C5B_7—C6B_7	1.530 (13)	C8B_15—H8F_15	0.9800
C5B_7—H5BA_7	0.9900	C9B_15—C10B_15	1.47 (2)
C5B_7—H5BB_7	0.9900	C9B_15—H9C_15	0.9900
C6B_7—C7B_7	1.510 (15)	C9B_15—H9D_15	0.9900
C6B_7—H6BA_7	0.9900	C10B_15—C11B_15	1.49 (2)
C6B_7—H6BB_7	0.9900	C10B_15—H10C_15	0.9900
C7B_7—C8B_7	1.513 (13)	C10B_15—H10D_15	0.9900
C7B_7—H7BA_7	0.9900	C11B_15—C12B_15	1.54 (2)
C7B_7—H7BB_7	0.9900	C11B_15—H11C_15	0.9900
C8B_7—C9B_7	1.510 (14)	C11B_15—H11D_15	0.9900
C8B_7—H8BA_7	0.9900	C12B_15—H12D_15	0.9800
C8B_7—H8BB_7	0.9900	C12B_15—H12E_15	0.9800
C9B_7—C10B_7	1.522 (14)	C12B_15—H12F_15	0.9800
C9B_7—H9BA_7	0.9900	C13B_15—C14B_15	1.55 (2)
C9B_7—H9BB_7	0.9900	C13B_15—H13C_15	0.9900
C10B_7—C11B_7	1.512 (16)	C13B_15—H13D_15	0.9900
C10B_7—H10C_7	0.9900	C14B_15—C15B_15	1.49 (2)
C10B_7—H10D_7	0.9900	C14B_15—H14C_15	0.9900
C11B_7—H11D_7	0.9800	C14B_15—H14D_15	0.9900
C11B_7—H11E_7	0.9800	C15B_15—C16B_15	1.52 (2)
C11B_7—H11F_7	0.9800	C15B_15—H15C_15	0.9900
N1_8—C1_8	1.336 (5)	C15B_15—H15D_15	0.9900
N1_8—N2_8	1.355 (5)	C16B_15—H16D_15	0.9800
N2_8—C3_8	1.342 (6)	C16B_15—H16E_15	0.9800
C1_8—C2_8	1.389 (6)	C16B_15—H16F_15	0.9800
			
N1_1—Ni1_16—N2_2	95.00 (18)	C5_7—C4_7—H4A_7	108.7
N1_1—Ni1_16—N2_6	97.14 (18)	C2_7—C4_7—H4B_7	108.7
N2_2—Ni1_16—N2_6	96.04 (18)	C5_7—C4_7—H4B_7	108.7
N1_1—Ni1_16—O2_16	91.57 (15)	H4A_7—C4_7—H4B_7	107.6
N2_2—Ni1_16—O2_16	170.24 (16)	C4_7—C5_7—C6_7	110.5 (11)
N2_6—Ni1_16—O2_16	90.27 (15)	C4_7—C5_7—H5A_7	109.5
N1_1—Ni1_16—O3_16^i^	89.85 (16)	C6_7—C5_7—H5A_7	109.5
N2_2—Ni1_16—O3_16^i^	91.28 (16)	C4_7—C5_7—H5B_7	109.5
N2_6—Ni1_16—O3_16^i^	169.37 (14)	C6_7—C5_7—H5B_7	109.5
O2_16—Ni1_16—O3_16^i^	81.51 (12)	H5A_7—C5_7—H5B_7	108.1
N1_1—Ni1_16—O1_16	169.68 (15)	C7_7—C6_7—C5_7	113.2 (16)
N2_2—Ni1_16—O1_16	90.71 (15)	C7_7—C6_7—H6A_7	108.9
N2_6—Ni1_16—O1_16	90.77 (15)	C5_7—C6_7—H6A_7	108.9
O2_16—Ni1_16—O1_16	81.74 (12)	C7_7—C6_7—H6B_7	108.9
O3_16^i^—Ni1_16—O1_16	81.42 (13)	C5_7—C6_7—H6B_7	108.9
N1_1—Ni1_16—Ni2_16	128.62 (15)	H6A_7—C6_7—H6B_7	107.7
N2_2—Ni1_16—Ni2_16	66.46 (13)	C6_7—C7_7—C8_7	115.6 (15)
N2_6—Ni1_16—Ni2_16	130.75 (12)	C6_7—C7_7—H7A_7	108.4
O2_16—Ni1_16—Ni2_16	103.78 (9)	C8_7—C7_7—H7A_7	108.4
O3_16^i^—Ni1_16—Ni2_16	46.30 (9)	C6_7—C7_7—H7B_7	108.4
O1_16—Ni1_16—Ni2_16	46.79 (9)	C8_7—C7_7—H7B_7	108.4
N1_1—Ni1_16—Ni3_16^i^	65.86 (12)	H7A_7—C7_7—H7B_7	107.5
N2_2—Ni1_16—Ni3_16^i^	131.11 (14)	C7_7—C8_7—C9_7	113.1 (15)
N2_6—Ni1_16—Ni3_16^i^	129.35 (13)	C7_7—C8_7—H8A_7	109.0
O2_16—Ni1_16—Ni3_16^i^	46.26 (9)	C9_7—C8_7—H8A_7	109.0
O3_16^i^—Ni1_16—Ni3_16^i^	47.12 (8)	C7_7—C8_7—H8B_7	109.0
O1_16—Ni1_16—Ni3_16^i^	103.96 (9)	C9_7—C8_7—H8B_7	109.0
Ni2_16—Ni1_16—Ni3_16^i^	90.29 (3)	H8A_7—C8_7—H8B_7	107.8
N1_1—Ni1_16—Ni4_16	132.24 (14)	C10_7—C9_7—C8_7	112.5 (15)
N2_2—Ni1_16—Ni4_16	129.54 (13)	C10_7—C9_7—H9A_7	109.1
N2_6—Ni1_16—Ni4_16	65.90 (12)	C8_7—C9_7—H9A_7	109.1
O2_16—Ni1_16—Ni4_16	47.19 (9)	C10_7—C9_7—H9B_7	109.1
O3_16^i^—Ni1_16—Ni4_16	103.48 (9)	C8_7—C9_7—H9B_7	109.1
O1_16—Ni1_16—Ni4_16	46.11 (9)	H9A_7—C9_7—H9B_7	107.8
Ni2_16—Ni1_16—Ni4_16	89.71 (4)	C11_7—C10_7—C9_7	110.0 (16)
Ni3_16^i^—Ni1_16—Ni4_16	90.21 (4)	C11_7—C10_7—H10A_7	109.7
N1_2—Ni2_16—N2_3	95.28 (17)	C9_7—C10_7—H10A_7	109.7
N1_2—Ni2_16—N2_5^i^	95.92 (19)	C11_7—C10_7—H10B_7	109.7
N2_3—Ni2_16—N2_5^i^	97.20 (18)	C9_7—C10_7—H10B_7	109.7
N1_2—Ni2_16—O3_16^i^	90.88 (15)	H10A_7—C10_7—H10B_7	108.2
N2_3—Ni2_16—O3_16^i^	169.57 (14)	C10_7—C11_7—H11A_7	109.5
N2_5^i^—Ni2_16—O3_16^i^	90.51 (15)	C10_7—C11_7—H11B_7	109.5
N1_2—Ni2_16—O1_16	91.81 (16)	H11A_7—C11_7—H11B_7	109.5
N2_3—Ni2_16—O1_16	89.10 (15)	C10_7—C11_7—H11C_7	109.5
N2_5^i^—Ni2_16—O1_16	169.52 (15)	H11A_7—C11_7—H11C_7	109.5
O3_16^i^—Ni2_16—O1_16	82.29 (12)	H11B_7—C11_7—H11C_7	109.5
N1_2—Ni2_16—O2_16^i^	170.33 (16)	C4_7—C5B_7—C6B_7	116.2 (9)
N2_3—Ni2_16—O2_16^i^	90.82 (14)	C4_7—C5B_7—H5BA_7	108.2
N2_5^i^—Ni2_16—O2_16^i^	90.75 (16)	C6B_7—C5B_7—H5BA_7	108.2
O3_16^i^—Ni2_16—O2_16^i^	82.03 (12)	C4_7—C5B_7—H5BB_7	108.2
O1_16—Ni2_16—O2_16^i^	80.76 (12)	C6B_7—C5B_7—H5BB_7	108.2
N1_2—Ni2_16—Ni1_16	66.52 (13)	H5BA_7—C5B_7—H5BB_7	107.4
N2_3—Ni2_16—Ni1_16	129.06 (13)	C7B_7—C6B_7—C5B_7	113.4 (14)
N2_5^i^—Ni2_16—Ni1_16	130.35 (13)	C7B_7—C6B_7—H6BA_7	108.9
O3_16^i^—Ni2_16—Ni1_16	46.73 (9)	C5B_7—C6B_7—H6BA_7	108.9
O1_16—Ni2_16—Ni1_16	47.43 (8)	C7B_7—C6B_7—H6BB_7	108.9
O2_16^i^—Ni2_16—Ni1_16	103.81 (9)	C5B_7—C6B_7—H6BB_7	108.9
N1_2—Ni2_16—Ni4_16^i^	130.21 (13)	H6BA_7—C6B_7—H6BB_7	107.7
N2_3—Ni2_16—Ni4_16^i^	131.35 (13)	C6B_7—C7B_7—C8B_7	114.5 (12)
N2_5^i^—Ni2_16—Ni4_16^i^	66.01 (13)	C6B_7—C7B_7—H7BA_7	108.6
O3_16^i^—Ni2_16—Ni4_16^i^	46.43 (9)	C8B_7—C7B_7—H7BA_7	108.6
O1_16—Ni2_16—Ni4_16^i^	103.56 (9)	C6B_7—C7B_7—H7BB_7	108.6
O2_16^i^—Ni2_16—Ni4_16^i^	47.12 (8)	C8B_7—C7B_7—H7BB_7	108.6
Ni1_16—Ni2_16—Ni4_16^i^	89.86 (3)	H7BA_7—C7B_7—H7BB_7	107.6
N1_3—Ni3_16—N1_4	95.82 (17)	C9B_7—C8B_7—C7B_7	115.7 (13)
N1_3—Ni3_16—N2_1^i^	98.03 (17)	C9B_7—C8B_7—H8BA_7	108.4
N1_4—Ni3_16—N2_1^i^	94.35 (17)	C7B_7—C8B_7—H8BA_7	108.4
N1_3—Ni3_16—O2_16^i^	91.85 (15)	C9B_7—C8B_7—H8BB_7	108.4
N1_4—Ni3_16—O2_16^i^	169.49 (15)	C7B_7—C8B_7—H8BB_7	108.4
N2_1^i^—Ni3_16—O2_16^i^	91.68 (15)	H8BA_7—C8B_7—H8BB_7	107.4
N1_3—Ni3_16—O1_16	89.74 (14)	C8B_7—C9B_7—C10B_7	116.7 (13)
N1_4—Ni3_16—O1_16	91.29 (15)	C8B_7—C9B_7—H9BA_7	108.1
N2_1^i^—Ni3_16—O1_16	169.88 (14)	C10B_7—C9B_7—H9BA_7	108.1
O2_16^i^—Ni3_16—O1_16	81.54 (12)	C8B_7—C9B_7—H9BB_7	108.1
N1_3—Ni3_16—O3_16	169.23 (14)	C10B_7—C9B_7—H9BB_7	108.1
N1_4—Ni3_16—O3_16	90.20 (15)	H9BA_7—C9B_7—H9BB_7	107.3
N2_1^i^—Ni3_16—O3_16	90.39 (14)	C11B_7—C10B_7—C9B_7	112.2 (14)
O2_16^i^—Ni3_16—O3_16	81.15 (12)	C11B_7—C10B_7—H10C_7	109.2
O1_16—Ni3_16—O3_16	81.18 (12)	C9B_7—C10B_7—H10C_7	109.2
N1_3—Ni3_16—Ni4_16	128.72 (13)	C11B_7—C10B_7—H10D_7	109.2
N1_4—Ni3_16—Ni4_16	66.33 (12)	C9B_7—C10B_7—H10D_7	109.2
N2_1^i^—Ni3_16—Ni4_16	129.49 (13)	H10C_7—C10B_7—H10D_7	107.9
O2_16^i^—Ni3_16—Ni4_16	103.23 (9)	C10B_7—C11B_7—H11D_7	109.5
O1_16—Ni3_16—Ni4_16	46.23 (9)	C10B_7—C11B_7—H11E_7	109.5
O3_16—Ni3_16—Ni4_16	46.48 (9)	H11D_7—C11B_7—H11E_7	109.5
N1_3—Ni3_16—Ni1_16^i^	131.72 (13)	C10B_7—C11B_7—H11F_7	109.5
N1_4—Ni3_16—Ni1_16^i^	129.31 (12)	H11D_7—C11B_7—H11F_7	109.5
N2_1^i^—Ni3_16—Ni1_16^i^	66.44 (12)	H11E_7—C11B_7—H11F_7	109.5
O2_16^i^—Ni3_16—Ni1_16^i^	46.43 (9)	C1_8—N1_8—N2_8	108.0 (3)
O1_16—Ni3_16—Ni1_16^i^	103.52 (9)	C1_8—N1_8—Ni5_16	139.0 (3)
O3_16—Ni3_16—Ni1_16^i^	46.50 (9)	N2_8—N1_8—Ni5_16	112.7 (2)
Ni4_16—Ni3_16—Ni1_16^i^	89.79 (3)	C3_8—N2_8—N1_8	107.2 (3)
N1_5—Ni4_16—N2_4	94.69 (17)	C3_8—N2_8—Ni6_16	137.9 (3)
N1_5—Ni4_16—N1_6	96.18 (19)	N1_8—N2_8—Ni6_16	114.8 (3)
N2_4—Ni4_16—N1_6	96.75 (17)	N1_8—C1_8—C2_8	111.0 (4)
N1_5—Ni4_16—O1_16	169.66 (15)	N1_8—C1_8—H1_8	124.5
N2_4—Ni4_16—O1_16	93.98 (15)	C2_8—C1_8—H1_8	124.5
N1_6—Ni4_16—O1_16	88.37 (16)	C3_8—C2_8—C1_8	102.6 (4)
N1_5—Ni4_16—O3_16	92.59 (16)	C3_8—C2_8—C4_8	126.6 (4)
N2_4—Ni4_16—O3_16	88.08 (15)	C1_8—C2_8—C4_8	130.8 (4)
N1_6—Ni4_16—O3_16	169.59 (15)	N2_8—C3_8—C2_8	111.3 (4)
O1_16—Ni4_16—O3_16	82.08 (12)	N2_8—C3_8—H3_8	124.4
N1_5—Ni4_16—O2_16	88.54 (15)	C2_8—C3_8—H3_8	124.4
N2_4—Ni4_16—O2_16	169.34 (15)	C2_8—C4_8—C5_8	116.1 (4)
N1_6—Ni4_16—O2_16	92.99 (15)	C2_8—C4_8—H4A_8	108.3
O1_16—Ni4_16—O2_16	81.94 (12)	C5_8—C4_8—H4A_8	108.3
O3_16—Ni4_16—O2_16	81.62 (12)	C2_8—C4_8—H4B_8	108.3
N1_5—Ni4_16—Ni3_16	133.37 (14)	C5_8—C4_8—H4B_8	108.3
N2_4—Ni4_16—Ni3_16	66.39 (12)	H4A_8—C4_8—H4B_8	107.4
N1_6—Ni4_16—Ni3_16	126.91 (13)	C6_8—C5_8—C4_8	116.5 (5)
O1_16—Ni4_16—Ni3_16	46.67 (9)	C6_8—C5_8—H5A_8	108.2
O3_16—Ni4_16—Ni3_16	47.15 (8)	C4_8—C5_8—H5A_8	108.2
O2_16—Ni4_16—Ni3_16	104.16 (9)	C6_8—C5_8—H5B_8	108.2
N1_5—Ni4_16—Ni1_16	126.76 (13)	C4_8—C5_8—H5B_8	108.2
N2_4—Ni4_16—Ni1_16	135.27 (12)	H5A_8—C5_8—H5B_8	107.3
N1_6—Ni4_16—Ni1_16	66.09 (13)	C5_8—C6_8—C7_8	113.2 (5)
O1_16—Ni4_16—Ni1_16	47.23 (8)	C5_8—C6_8—H6A_8	108.9
O3_16—Ni4_16—Ni1_16	104.07 (9)	C7_8—C6_8—H6A_8	108.9
O2_16—Ni4_16—Ni1_16	46.32 (8)	C5_8—C6_8—H6B_8	108.9
Ni3_16—Ni4_16—Ni1_16	90.62 (4)	C7_8—C6_8—H6B_8	108.9
N1_5—Ni4_16—Ni2_16^i^	66.19 (13)	H6A_8—C6_8—H6B_8	107.8
N2_4—Ni4_16—Ni2_16^i^	125.65 (12)	C8_8—C7_8—C6_8	115.4 (6)
N1_6—Ni4_16—Ni2_16^i^	133.89 (12)	C8_8—C7_8—H7A_8	108.4
O1_16—Ni4_16—Ni2_16^i^	104.05 (9)	C6_8—C7_8—H7A_8	108.4
O3_16—Ni4_16—Ni2_16^i^	46.05 (8)	C8_8—C7_8—H7B_8	108.4
O2_16—Ni4_16—Ni2_16^i^	47.00 (9)	C6_8—C7_8—H7B_8	108.4
Ni3_16—Ni4_16—Ni2_16^i^	90.05 (3)	H7A_8—C7_8—H7B_8	107.5
Ni1_16—Ni4_16—Ni2_16^i^	90.12 (4)	C7_8—C8_8—C9_8	113.5 (6)
N2_7—Ni5_16—N2_12^ii^	95.27 (15)	C7_8—C8_8—H8A_8	108.9
N2_7—Ni5_16—N1_8	94.94 (15)	C9_8—C8_8—H8A_8	108.9
N2_12^ii^—Ni5_16—N1_8	97.94 (15)	C7_8—C8_8—H8B_8	108.9
N2_7—Ni5_16—O6_16	91.02 (13)	C9_8—C8_8—H8B_8	108.9
N2_12^ii^—Ni5_16—O6_16	90.20 (14)	H8A_8—C8_8—H8B_8	107.7
N1_8—Ni5_16—O6_16	169.41 (12)	C10_8—C9_8—C8_8	114.5 (7)
N2_7—Ni5_16—O5_16^ii^	170.36 (13)	C10_8—C9_8—H9A_8	108.6
N2_12^ii^—Ni5_16—O5_16^ii^	90.79 (14)	C8_8—C9_8—H9A_8	108.6
N1_8—Ni5_16—O5_16^ii^	91.62 (13)	C10_8—C9_8—H9B_8	108.6
O6_16—Ni5_16—O5_16^ii^	81.46 (11)	C8_8—C9_8—H9B_8	108.6
N2_7—Ni5_16—O4_16	91.06 (13)	H9A_8—C9_8—H9B_8	107.6
N2_12^ii^—Ni5_16—O4_16	169.53 (13)	C9_8—C10_8—C11_8	112.6 (8)
N1_8—Ni5_16—O4_16	89.79 (13)	C9_8—C10_8—H10A_8	109.1
O6_16—Ni5_16—O4_16	81.35 (11)	C11_8—C10_8—H10A_8	109.1
O5_16^ii^—Ni5_16—O4_16	81.90 (11)	C9_8—C10_8—H10B_8	109.1
N2_7—Ni5_16—Ni8_16	66.19 (11)	C11_8—C10_8—H10B_8	109.1
N2_12^ii^—Ni5_16—Ni8_16	129.32 (11)	H10A_8—C10_8—H10B_8	107.8
N1_8—Ni5_16—Ni8_16	128.93 (11)	C10_8—C11_8—H11A_8	109.5
O6_16—Ni5_16—Ni8_16	46.59 (8)	C10_8—C11_8—H11B_8	109.5
O5_16^ii^—Ni5_16—Ni8_16	104.18 (8)	H11A_8—C11_8—H11B_8	109.5
O4_16—Ni5_16—Ni8_16	46.72 (8)	C10_8—C11_8—H11C_8	109.5
N2_7—Ni5_16—Ni6_16	130.75 (11)	H11A_8—C11_8—H11C_8	109.5
N2_12^ii^—Ni5_16—Ni6_16	130.84 (11)	H11B_8—C11_8—H11C_8	109.5
N1_8—Ni5_16—Ni6_16	66.27 (10)	C1_9—N1_9—N2_9	108.2 (4)
O6_16—Ni5_16—Ni6_16	103.26 (8)	C1_9—N1_9—Ni8_16	136.9 (3)
O5_16^ii^—Ni5_16—Ni6_16	46.62 (8)	N2_9—N1_9—Ni8_16	114.0 (3)
O4_16—Ni5_16—Ni6_16	46.70 (8)	C3_9—N2_9—N1_9	107.2 (4)
Ni8_16—Ni5_16—Ni6_16	90.18 (4)	C3_9—N2_9—Ni7_16	139.2 (3)
N2_8—Ni6_16—N1_11	96.21 (16)	N1_9—N2_9—Ni7_16	113.4 (3)
N2_8—Ni6_16—N1_10	96.19 (16)	N1_9—C1_9—C2_9	110.7 (4)
N1_11—Ni6_16—N1_10	94.92 (17)	N1_9—C1_9—H1_9	124.7
N2_8—Ni6_16—O6_16^ii^	169.19 (13)	C2_9—C1_9—H1_9	124.7
N1_11—Ni6_16—O6_16^ii^	92.73 (13)	C3_9—C2_9—C1_9	102.8 (4)
N1_10—Ni6_16—O6_16^ii^	89.06 (14)	C3_9—C2_9—C4_9	128.4 (5)
N2_8—Ni6_16—O5_16^ii^	92.29 (14)	C1_9—C2_9—C4_9	128.7 (4)
N1_11—Ni6_16—O5_16^ii^	89.41 (14)	N2_9—C3_9—C2_9	111.1 (4)
N1_10—Ni6_16—O5_16^ii^	170.00 (14)	N2_9—C3_9—H3_9	124.4
O6_16^ii^—Ni6_16—O5_16^ii^	81.72 (11)	C2_9—C3_9—H3_9	124.4
N2_8—Ni6_16—O4_16	89.20 (13)	C2_9—C4_9—C5_9	113.5 (4)
N1_11—Ni6_16—O4_16	170.19 (14)	C2_9—C4_9—H4A_9	108.9
N1_10—Ni6_16—O4_16	92.62 (14)	C5_9—C4_9—H4A_9	108.9
O6_16^ii^—Ni6_16—O4_16	81.11 (11)	C2_9—C4_9—H4B_9	108.9
O5_16^ii^—Ni6_16—O4_16	82.19 (11)	C5_9—C4_9—H4B_9	108.9
N2_8—Ni6_16—Ni8_16^ii^	132.80 (12)	H4A_9—C4_9—H4B_9	107.7
N1_11—Ni6_16—Ni8_16^ii^	66.24 (11)	C6_9—C5_9—C4_9	114.3 (5)
N1_10—Ni6_16—Ni8_16^ii^	127.36 (11)	C6_9—C5_9—H5A_9	108.7
O6_16^ii^—Ni6_16—Ni8_16^ii^	46.73 (8)	C4_9—C5_9—H5A_9	108.7
O5_16^ii^—Ni6_16—Ni8_16^ii^	46.90 (8)	C6_9—C5_9—H5B_9	108.7
O4_16—Ni6_16—Ni8_16^ii^	104.13 (8)	C4_9—C5_9—H5B_9	108.7
N2_8—Ni6_16—Ni5_16	66.20 (11)	H5A_9—C5_9—H5B_9	107.6
N1_11—Ni6_16—Ni5_16	128.58 (12)	C7_9—C6_9—C5_9	116.0 (5)
N1_10—Ni6_16—Ni5_16	133.13 (12)	C7_9—C6_9—H6A_9	108.3
O6_16^ii^—Ni6_16—Ni5_16	103.38 (8)	C5_9—C6_9—H6A_9	108.3
O5_16^ii^—Ni6_16—Ni5_16	46.79 (8)	C7_9—C6_9—H6B_9	108.3
O4_16—Ni6_16—Ni5_16	46.88 (8)	C5_9—C6_9—H6B_9	108.3
Ni8_16^ii^—Ni6_16—Ni5_16	90.34 (4)	H6A_9—C6_9—H6B_9	107.4
N2_8—Ni6_16—Ni7_16	127.44 (12)	C8_9—C7_9—C6_9	113.4 (5)
N1_11—Ni6_16—Ni7_16	132.64 (12)	C8_9—C7_9—H7A_9	108.9
N1_10—Ni6_16—Ni7_16	66.20 (11)	C6_9—C7_9—H7A_9	108.9
O6_16^ii^—Ni6_16—Ni7_16	46.80 (8)	C8_9—C7_9—H7B_9	108.9
O5_16^ii^—Ni6_16—Ni7_16	104.34 (8)	C6_9—C7_9—H7B_9	108.9
O4_16—Ni6_16—Ni7_16	46.13 (8)	H7A_9—C7_9—H7B_9	107.7
Ni8_16^ii^—Ni6_16—Ni7_16	90.56 (3)	C7_9—C8_9—C9_9	114.9 (6)
Ni5_16—Ni6_16—Ni7_16	89.88 (4)	C7_9—C8_9—H8A_9	108.5
N1_12—Ni7_16—N2_9	97.77 (16)	C9_9—C8_9—H8A_9	108.5
N1_12—Ni7_16—N2_10	95.08 (16)	C7_9—C8_9—H8B_9	108.5
N2_9—Ni7_16—N2_10	95.43 (16)	C9_9—C8_9—H8B_9	108.5
N1_12—Ni7_16—O4_16	169.64 (14)	H8A_9—C8_9—H8B_9	107.5
N2_9—Ni7_16—O4_16	89.17 (13)	C8_9—C9_9—C10_9	114.2 (6)
N2_10—Ni7_16—O4_16	91.89 (13)	C8_9—C9_9—H9A_9	108.7
N1_12—Ni7_16—O5_16	90.29 (13)	C10_9—C9_9—H9A_9	108.7
N2_9—Ni7_16—O5_16	92.73 (13)	C8_9—C9_9—H9B_9	108.7
N2_10—Ni7_16—O5_16	169.53 (14)	C10_9—C9_9—H9B_9	108.7
O4_16—Ni7_16—O5_16	81.67 (11)	H9A_9—C9_9—H9B_9	107.6
N1_12—Ni7_16—O6_16^ii^	90.97 (14)	C11_9—C10_9—C9_9	113.6 (8)
N2_9—Ni7_16—O6_16^ii^	169.26 (13)	C11_9—C10_9—H10A_9	108.8
N2_10—Ni7_16—O6_16^ii^	89.96 (14)	C9_9—C10_9—H10A_9	108.8
O4_16—Ni7_16—O6_16^ii^	81.36 (11)	C11_9—C10_9—H10B_9	108.8
O5_16—Ni7_16—O6_16^ii^	80.95 (11)	C9_9—C10_9—H10B_9	108.8
N1_12—Ni7_16—Ni6_16	130.36 (12)	H10A_9—C10_9—H10B_9	107.7
N2_9—Ni7_16—Ni6_16	128.11 (11)	C10_9—C11_9—H11A_9	109.5
N2_10—Ni7_16—Ni6_16	66.15 (11)	C10_9—C11_9—H11B_9	109.5
O4_16—Ni7_16—Ni6_16	46.72 (8)	H11A_9—C11_9—H11B_9	109.5
O5_16—Ni7_16—Ni6_16	103.58 (8)	C10_9—C11_9—H11C_9	109.5
O6_16^ii^—Ni7_16—Ni6_16	46.51 (8)	H11A_9—C11_9—H11C_9	109.5
N1_12—Ni7_16—Ni8_16	129.86 (12)	H11B_9—C11_9—H11C_9	109.5
N2_9—Ni7_16—Ni8_16	66.48 (10)	C1_10—N1_10—N2_10	107.6 (4)
N2_10—Ni7_16—Ni8_16	132.08 (11)	C1_10—N1_10—Ni6_16	137.6 (4)
O4_16—Ni7_16—Ni8_16	46.63 (8)	N2_10—N1_10—Ni6_16	113.6 (3)
O5_16—Ni7_16—Ni8_16	46.62 (7)	C3_10—N2_10—N1_10	107.9 (4)
O6_16^ii^—Ni7_16—Ni8_16	103.09 (8)	C3_10—N2_10—Ni7_16	136.1 (3)
Ni6_16—Ni7_16—Ni8_16	90.02 (4)	N1_10—N2_10—Ni7_16	114.0 (3)
N2_11^ii^—Ni8_16—N1_7	95.29 (16)	N1_10—C1_10—C2_10	110.7 (4)
N2_11^ii^—Ni8_16—N1_9	96.67 (16)	N1_10—C1_10—H1_10	124.7
N1_7—Ni8_16—N1_9	95.43 (15)	C2_10—C1_10—H1_10	124.7
N2_11^ii^—Ni8_16—O6_16	91.49 (14)	C1_10—C2_10—C3_10	103.4 (4)
N1_7—Ni8_16—O6_16	90.78 (13)	C1_10—C2_10—C4B_10	128.4 (5)
N1_9—Ni8_16—O6_16	169.23 (13)	C3_10—C2_10—C4B_10	127.8 (5)
N2_11^ii^—Ni8_16—O4_16	169.90 (14)	C1_10—C2_10—C4_10	128.4 (5)
N1_7—Ni8_16—O4_16	91.77 (13)	C3_10—C2_10—C4_10	127.8 (5)
N1_9—Ni8_16—O4_16	89.81 (14)	N2_10—C3_10—C2_10	110.4 (5)
O6_16—Ni8_16—O4_16	81.18 (11)	N2_10—C3_10—H3_10	124.8
N2_11^ii^—Ni8_16—O5_16	90.91 (14)	C2_10—C3_10—H3_10	124.8
N1_7—Ni8_16—O5_16	170.40 (13)	C2_10—C4_10—C5_10	112.3 (9)
N1_9—Ni8_16—O5_16	91.11 (13)	C2_10—C4_10—H4A_10	109.1
O6_16—Ni8_16—O5_16	81.72 (11)	C5_10—C4_10—H4A_10	109.1
O4_16—Ni8_16—O5_16	81.21 (11)	C2_10—C4_10—H4B_10	109.1
N2_11^ii^—Ni8_16—Ni6_16^ii^	66.39 (12)	C5_10—C4_10—H4B_10	109.1
N1_7—Ni8_16—Ni6_16^ii^	129.94 (11)	H4A_10—C4_10—H4B_10	107.9
N1_9—Ni8_16—Ni6_16^ii^	131.27 (11)	C6_10—C5_10—C4_10	112.0 (13)
O6_16—Ni8_16—Ni6_16^ii^	46.78 (8)	C6_10—C5_10—H5A_10	109.2
O4_16—Ni8_16—Ni6_16^ii^	103.53 (8)	C4_10—C5_10—H5A_10	109.2
O5_16—Ni8_16—Ni6_16^ii^	46.86 (8)	C6_10—C5_10—H5B_10	109.2
N2_11^ii^—Ni8_16—Ni5_16	130.42 (12)	C4_10—C5_10—H5B_10	109.2
N1_7—Ni8_16—Ni5_16	66.58 (11)	H5A_10—C5_10—H5B_10	107.9
N1_9—Ni8_16—Ni5_16	129.32 (11)	C7_10—C6_10—C5_10	115.0 (14)
O6_16—Ni8_16—Ni5_16	46.16 (8)	C7_10—C6_10—H6A_10	108.5
O4_16—Ni8_16—Ni5_16	46.95 (8)	C5_10—C6_10—H6A_10	108.5
O5_16—Ni8_16—Ni5_16	103.83 (8)	C7_10—C6_10—H6B_10	108.5
Ni6_16^ii^—Ni8_16—Ni5_16	89.91 (3)	C5_10—C6_10—H6B_10	108.5
N2_11^ii^—Ni8_16—Ni7_16	130.43 (12)	H6A_10—C6_10—H6B_10	107.5
N1_7—Ni8_16—Ni7_16	130.77 (11)	C6_10—C7_10—C8_10	113.2 (14)
N1_9—Ni8_16—Ni7_16	66.01 (10)	C6_10—C7_10—H7A_10	108.9
O6_16—Ni8_16—Ni7_16	103.30 (8)	C8_10—C7_10—H7A_10	108.9
O4_16—Ni8_16—Ni7_16	46.09 (8)	C6_10—C7_10—H7B_10	108.9
O5_16—Ni8_16—Ni7_16	46.59 (8)	C8_10—C7_10—H7B_10	108.9
Ni6_16^ii^—Ni8_16—Ni7_16	90.12 (4)	H7A_10—C7_10—H7B_10	107.7
Ni5_16—Ni8_16—Ni7_16	89.92 (4)	C9_10—C8_10—C7_10	110.1 (15)
Ni4_16—O1_16—Ni3_16	87.10 (12)	C9_10—C8_10—H8A_10	109.6
Ni4_16—O1_16—Ni2_16	152.37 (16)	C7_10—C8_10—H8A_10	109.6
Ni3_16—O1_16—Ni2_16	87.46 (13)	C9_10—C8_10—H8B_10	109.6
Ni4_16—O1_16—Ni1_16	86.66 (12)	C7_10—C8_10—H8B_10	109.6
Ni3_16—O1_16—Ni1_16	152.52 (16)	H8A_10—C8_10—H8B_10	108.2
Ni2_16—O1_16—Ni1_16	85.78 (12)	C8_10—C9_10—C10_10	121.4 (19)
Ni4_16—O1_16—H1_16	104.3 (11)	C8_10—C9_10—H9A_10	107.0
Ni3_16—O1_16—H1_16	104.6 (11)	C10_10—C9_10—H9A_10	107.0
Ni2_16—O1_16—H1_16	103.3 (11)	C8_10—C9_10—H9B_10	107.0
Ni1_16—O1_16—H1_16	102.9 (11)	C10_10—C9_10—H9B_10	107.0
Ni3_16^i^—O2_16—Ni1_16	87.31 (12)	H9A_10—C9_10—H9B_10	106.7
Ni3_16^i^—O2_16—Ni2_16^i^	87.38 (12)	C11_10—C10_10—C9_10	85.6 (15)
Ni1_16—O2_16—Ni2_16^i^	152.39 (16)	C11_10—C10_10—H10A_10	114.4
Ni3_16^i^—O2_16—Ni4_16	152.62 (16)	C9_10—C10_10—H10A_10	114.4
Ni1_16—O2_16—Ni4_16	86.49 (11)	C11_10—C10_10—H10B_10	114.4
Ni2_16^i^—O2_16—Ni4_16	85.87 (11)	C9_10—C10_10—H10B_10	114.4
Ni3_16^i^—O2_16—H2_16	105.3 (11)	H10A_10—C10_10—H10B_10	111.5
Ni1_16—O2_16—H2_16	105.3 (11)	C10_10—C11_10—H11A_10	109.5
Ni2_16^i^—O2_16—H2_16	102.3 (11)	C10_10—C11_10—H11B_10	109.5
Ni4_16—O2_16—H2_16	102.1 (11)	H11A_10—C11_10—H11B_10	109.5
Ni2_16^i^—O3_16—Ni4_16	87.51 (12)	C10_10—C11_10—H11C_10	109.5
Ni2_16^i^—O3_16—Ni1_16^i^	86.98 (12)	H11A_10—C11_10—H11C_10	109.5
Ni4_16—O3_16—Ni1_16^i^	152.43 (15)	H11B_10—C11_10—H11C_10	109.5
Ni2_16^i^—O3_16—Ni3_16	152.96 (15)	C5B_10—C4B_10—C2_10	112.5 (10)
Ni4_16—O3_16—Ni3_16	86.37 (12)	C5B_10—C4B_10—H4BA_10	109.1
Ni1_16^i^—O3_16—Ni3_16	86.37 (12)	C2_10—C4B_10—H4BA_10	109.1
Ni2_16^i^—O3_16—H3_16	104.5 (11)	C5B_10—C4B_10—H4BB_10	109.1
Ni4_16—O3_16—H3_16	104.1 (11)	C2_10—C4B_10—H4BB_10	109.1
Ni1_16^i^—O3_16—H3_16	103.5 (11)	H4BA_10—C4B_10—H4BB_10	107.8
Ni3_16—O3_16—H3_16	102.6 (11)	C4B_10—C5B_10—C6B_10	115.7 (14)
Ni7_16—O4_16—Ni8_16	87.27 (11)	C4B_10—C5B_10—H5BA_10	108.3
Ni7_16—O4_16—Ni6_16	87.15 (10)	C6B_10—C5B_10—H5BA_10	108.3
Ni8_16—O4_16—Ni6_16	152.34 (15)	C4B_10—C5B_10—H5BB_10	108.3
Ni7_16—O4_16—Ni5_16	152.92 (15)	C6B_10—C5B_10—H5BB_10	108.3
Ni8_16—O4_16—Ni5_16	86.33 (11)	H5BA_10—C5B_10—H5BB_10	107.4
Ni6_16—O4_16—Ni5_16	86.42 (11)	C7B_10—C6B_10—C5B_10	113.2 (14)
Ni7_16—O4_16—H4_16	104.6 (11)	C7B_10—C6B_10—H6BA_10	108.9
Ni8_16—O4_16—H4_16	103.8 (11)	C5B_10—C6B_10—H6BA_10	108.9
Ni6_16—O4_16—H4_16	103.8 (11)	C7B_10—C6B_10—H6BB_10	108.9
Ni5_16—O4_16—H4_16	102.4 (11)	C5B_10—C6B_10—H6BB_10	108.9
Ni6_16^ii^—O5_16—Ni7_16	152.08 (15)	H6BA_10—C6B_10—H6BB_10	107.7
Ni6_16^ii^—O5_16—Ni8_16	86.24 (11)	C8B_10—C7B_10—C6B_10	113.6 (15)
Ni7_16—O5_16—Ni8_16	86.79 (11)	C8B_10—C7B_10—H7BA_10	108.8
Ni6_16^ii^—O5_16—Ni5_16^ii^	86.59 (12)	C6B_10—C7B_10—H7BA_10	108.8
Ni7_16—O5_16—Ni5_16^ii^	86.99 (11)	C8B_10—C7B_10—H7BB_10	108.8
Ni8_16—O5_16—Ni5_16^ii^	151.98 (15)	C6B_10—C7B_10—H7BB_10	108.8
Ni6_16^ii^—O5_16—H5_16	104.1 (11)	H7BA_10—C7B_10—H7BB_10	107.7
Ni7_16—O5_16—H5_16	103.9 (11)	C7B_10—C8B_10—C9B_10	115.1 (16)
Ni8_16—O5_16—H5_16	104.7 (11)	C7B_10—C8B_10—H8BA_10	108.5
Ni5_16^ii^—O5_16—H5_16	103.3 (11)	C9B_10—C8B_10—H8BA_10	108.5
Ni5_16—O6_16—Ni8_16	87.25 (11)	C7B_10—C8B_10—H8BB_10	108.5
Ni5_16—O6_16—Ni6_16^ii^	153.36 (14)	C9B_10—C8B_10—H8BB_10	108.5
Ni8_16—O6_16—Ni6_16^ii^	86.49 (11)	H8BA_10—C8B_10—H8BB_10	107.5
Ni5_16—O6_16—Ni7_16^ii^	87.51 (11)	C8B_10—C9B_10—C10B_10	103.5 (16)
Ni8_16—O6_16—Ni7_16^ii^	153.60 (14)	C8B_10—C9B_10—H9BA_10	111.1
Ni6_16^ii^—O6_16—Ni7_16^ii^	86.69 (11)	C10B_10—C9B_10—H9BA_10	111.1
Ni5_16—O6_16—H6_16	103.8 (10)	C8B_10—C9B_10—H9BB_10	111.1
Ni8_16—O6_16—H6_16	103.3 (11)	C10B_10—C9B_10—H9BB_10	111.1
Ni6_16^ii^—O6_16—H6_16	102.8 (11)	H9BA_10—C9B_10—H9BB_10	109.0
Ni7_16^ii^—O6_16—H6_16	103.1 (11)	C11B_10—C10B_10—C9B_10	89.3 (16)
C1_1—N1_1—N2_1	107.9 (4)	C11B_10—C10B_10—H10C_10	113.8
C1_1—N1_1—Ni1_16	135.5 (4)	C9B_10—C10B_10—H10C_10	113.8
N2_1—N1_1—Ni1_16	115.5 (3)	C11B_10—C10B_10—H10D_10	113.8
C3_1—N2_1—N1_1	107.1 (4)	C9B_10—C10B_10—H10D_10	113.8
C3_1—N2_1—Ni3_16^i^	139.2 (4)	H10C_10—C10B_10—H10D_10	111.0
N1_1—N2_1—Ni3_16^i^	112.1 (3)	C10B_10—C11B_10—H11D_10	109.5
N1_1—C1_1—C2_1	110.8 (5)	C10B_10—C11B_10—H11E_10	109.5
N1_1—C1_1—H1_1	124.6	H11D_10—C11B_10—H11E_10	109.5
C2_1—C1_1—H1_1	124.6	C10B_10—C11B_10—H11F_10	109.5
C3_1—C2_1—C1_1	102.9 (4)	H11D_10—C11B_10—H11F_10	109.5
C3_1—C2_1—C4_1	130.0 (5)	H11E_10—C11B_10—H11F_10	109.5
C1_1—C2_1—C4_1	127.0 (5)	C1_11—N1_11—N2_11	107.5 (4)
N2_1—C3_1—C2_1	111.3 (5)	C1_11—N1_11—Ni6_16	138.5 (3)
N2_1—C3_1—H3_1	124.4	N2_11—N1_11—Ni6_16	113.3 (3)
C2_1—C3_1—H3_1	124.4	C3_11—N2_11—N1_11	107.5 (4)
C5_1—C4_1—C2_1	113.8 (5)	C3_11—N2_11—Ni8_16^ii^	138.6 (3)
C5_1—C4_1—H4A_1	108.8	N1_11—N2_11—Ni8_16^ii^	113.8 (3)
C2_1—C4_1—H4A_1	108.8	N1_11—C1_11—C2_11	110.8 (4)
C5_1—C4_1—H4B_1	108.8	N1_11—C1_11—H1_11	124.6
C2_1—C4_1—H4B_1	108.8	C2_11—C1_11—H1_11	124.6
H4A_1—C4_1—H4B_1	107.7	C3_11—C2_11—C1_11	103.6 (4)
C4_1—C5_1—C6_1	116.7 (6)	C3_11—C2_11—C4_11	128.6 (4)
C4_1—C5_1—H5A_1	108.1	C1_11—C2_11—C4_11	127.5 (5)
C6_1—C5_1—H5A_1	108.1	N2_11—C3_11—C2_11	110.6 (4)
C4_1—C5_1—H5B_1	108.1	N2_11—C3_11—H3_11	124.7
C6_1—C5_1—H5B_1	108.1	C2_11—C3_11—H3_11	124.7
H5A_1—C5_1—H5B_1	107.3	C2_11—C4_11—C5_11	112.7 (4)
C5_1—C6_1—C7_1	112.7 (7)	C2_11—C4_11—H4A_11	109.0
C5_1—C6_1—H6A_1	109.0	C5_11—C4_11—H4A_11	109.0
C7_1—C6_1—H6A_1	109.0	C2_11—C4_11—H4B_11	109.0
C5_1—C6_1—H6B_1	109.0	C5_11—C4_11—H4B_11	109.0
C7_1—C6_1—H6B_1	109.0	H4A_11—C4_11—H4B_11	107.8
H6A_1—C6_1—H6B_1	107.8	C6_11—C5_11—C4_11	113.9 (4)
C8_1—C7_1—C6_1	114.9 (8)	C6_11—C5_11—H5A_11	108.8
C8_1—C7_1—H7A_1	108.5	C4_11—C5_11—H5A_11	108.8
C6_1—C7_1—H7A_1	108.5	C6_11—C5_11—H5B_11	108.8
C8_1—C7_1—H7B_1	108.5	C4_11—C5_11—H5B_11	108.8
C6_1—C7_1—H7B_1	108.5	H5A_11—C5_11—H5B_11	107.7
H7A_1—C7_1—H7B_1	107.5	C5_11—C6_11—C7_11	113.3 (5)
C7_1—C8_1—C9_1	113.8 (9)	C5_11—C6_11—H6A_11	108.9
C7_1—C8_1—H8A_1	108.8	C7_11—C6_11—H6A_11	108.9
C9_1—C8_1—H8A_1	108.8	C5_11—C6_11—H6B_11	108.9
C7_1—C8_1—H8B_1	108.8	C7_11—C6_11—H6B_11	108.9
C9_1—C8_1—H8B_1	108.8	H6A_11—C6_11—H6B_11	107.7
H8A_1—C8_1—H8B_1	107.7	C8_11—C7_11—C6_11	114.0 (5)
C8_1—C9_1—C10_1	113.6 (10)	C8_11—C7_11—H7A_11	108.7
C8_1—C9_1—H9A_1	108.8	C6_11—C7_11—H7A_11	108.7
C10_1—C9_1—H9A_1	108.8	C8_11—C7_11—H7B_11	108.7
C8_1—C9_1—H9B_1	108.8	C6_11—C7_11—H7B_11	108.7
C10_1—C9_1—H9B_1	108.8	H7A_11—C7_11—H7B_11	107.6
H9A_1—C9_1—H9B_1	107.7	C7_11—C8_11—C9_11	115.1 (5)
C11_1—C10_1—C9_1	111.0 (12)	C7_11—C8_11—H8A_11	108.5
C11_1—C10_1—H10A_1	109.4	C9_11—C8_11—H8A_11	108.5
C9_1—C10_1—H10A_1	109.4	C7_11—C8_11—H8B_11	108.5
C11_1—C10_1—H10B_1	109.4	C9_11—C8_11—H8B_11	108.5
C9_1—C10_1—H10B_1	109.4	H8A_11—C8_11—H8B_11	107.5
H10A_1—C10_1—H10B_1	108.0	C10_11—C9_11—C8_11	113.6 (6)
C10_1—C11_1—H11A_1	109.5	C10_11—C9_11—H9A_11	108.8
C10_1—C11_1—H11B_1	109.5	C8_11—C9_11—H9A_11	108.8
H11A_1—C11_1—H11B_1	109.5	C10_11—C9_11—H9B_11	108.8
C10_1—C11_1—H11C_1	109.5	C8_11—C9_11—H9B_11	108.8
H11A_1—C11_1—H11C_1	109.5	H9A_11—C9_11—H9B_11	107.7
H11B_1—C11_1—H11C_1	109.5	C11_11—C10_11—C9_11	113.3 (7)
C1_2—N1_2—N2_2	107.9 (4)	C11_11—C10_11—H10A_11	108.9
C1_2—N1_2—Ni2_16	138.5 (4)	C9_11—C10_11—H10A_11	108.9
N2_2—N1_2—Ni2_16	113.6 (3)	C11_11—C10_11—H10B_11	108.9
C3_2—N2_2—N1_2	107.1 (5)	C9_11—C10_11—H10B_11	108.9
C3_2—N2_2—Ni1_16	139.5 (4)	H10A_11—C10_11—H10B_11	107.7
N1_2—N2_2—Ni1_16	113.3 (3)	C10_11—C11_11—H11A_11	109.5
N1_2—C1_2—C2_2	110.9 (6)	C10_11—C11_11—H11B_11	109.5
N1_2—C1_2—H1_2	124.6	H11A_11—C11_11—H11B_11	109.5
C2_2—C1_2—H1_2	124.6	C10_11—C11_11—H11C_11	109.5
C3_2—C2_2—C1_2	103.7 (5)	H11A_11—C11_11—H11C_11	109.5
C3_2—C2_2—C4_2	130.2 (7)	H11B_11—C11_11—H11C_11	109.5
C1_2—C2_2—C4_2	126.1 (7)	C1_12—N1_12—N2_12	106.7 (3)
N2_2—C3_2—C2_2	110.5 (6)	C1_12—N1_12—Ni7_16	139.2 (3)
N2_2—C3_2—H3_2	124.8	N2_12—N1_12—Ni7_16	113.1 (3)
C2_2—C3_2—H3_2	124.8	C3_12—N2_12—N1_12	107.7 (4)
C5_2—C4_2—C2_2	118.3 (6)	C3_12—N2_12—Ni5_16^ii^	137.3 (3)
C5_2—C4_2—H4A_2	107.7	N1_12—N2_12—Ni5_16^ii^	114.6 (3)
C2_2—C4_2—H4A_2	107.7	N1_12—C1_12—C2_12	111.9 (4)
C5_2—C4_2—H4B_2	107.7	N1_12—C1_12—H1_12	124.1
C2_2—C4_2—H4B_2	107.7	C2_12—C1_12—H1_12	124.1
H4A_2—C4_2—H4B_2	107.1	C1_12—C2_12—C3_12	102.4 (4)
C4_2—C5_2—C6_2	114.8 (6)	C1_12—C2_12—C4_12	129.7 (4)
C4_2—C5_2—H5A_2	108.6	C3_12—C2_12—C4_12	127.7 (4)
C6_2—C5_2—H5A_2	108.6	N2_12—C3_12—C2_12	111.3 (4)
C4_2—C5_2—H5B_2	108.6	N2_12—C3_12—H3_12	124.4
C6_2—C5_2—H5B_2	108.6	C2_12—C3_12—H3_12	124.4
H5A_2—C5_2—H5B_2	107.5	C2_12—C4_12—C5_12	113.2 (4)
C7_2—C6_2—C5_2	120.4 (6)	C2_12—C4_12—H4A_12	108.9
C7_2—C6_2—H6A_2	107.2	C5_12—C4_12—H4A_12	108.9
C5_2—C6_2—H6A_2	107.2	C2_12—C4_12—H4B_12	108.9
C7_2—C6_2—H6B_2	107.2	C5_12—C4_12—H4B_12	108.9
C5_2—C6_2—H6B_2	107.2	H4A_12—C4_12—H4B_12	107.7
H6A_2—C6_2—H6B_2	106.9	C4_12—C5_12—C6_12	113.1 (4)
C6_2—C7_2—C8_2	117.2 (6)	C4_12—C5_12—H5A_12	108.9
C6_2—C7_2—H7A_2	108.0	C6_12—C5_12—H5A_12	108.9
C8_2—C7_2—H7A_2	108.0	C4_12—C5_12—H5B_12	108.9
C6_2—C7_2—H7B_2	108.0	C6_12—C5_12—H5B_12	108.9
C8_2—C7_2—H7B_2	108.0	H5A_12—C5_12—H5B_12	107.8
H7A_2—C7_2—H7B_2	107.3	C7_12—C6_12—C5_12	113.3 (4)
C9_2—C8_2—C7_2	120.3 (6)	C7_12—C6_12—H6A_12	108.9
C9_2—C8_2—H8A_2	107.2	C5_12—C6_12—H6A_12	108.9
C7_2—C8_2—H8A_2	107.2	C7_12—C6_12—H6B_12	108.9
C9_2—C8_2—H8B_2	107.2	C5_12—C6_12—H6B_12	108.9
C7_2—C8_2—H8B_2	107.2	H6A_12—C6_12—H6B_12	107.7
H8A_2—C8_2—H8B_2	106.9	C8_12—C7_12—C6_12	112.7 (4)
C8_2—C9_2—C10_2	117.8 (6)	C8_12—C7_12—H7A_12	109.0
C8_2—C9_2—H9A_2	107.9	C6_12—C7_12—H7A_12	109.0
C10_2—C9_2—H9A_2	107.9	C8_12—C7_12—H7B_12	109.0
C8_2—C9_2—H9B_2	107.9	C6_12—C7_12—H7B_12	109.0
C10_2—C9_2—H9B_2	107.9	H7A_12—C7_12—H7B_12	107.8
H9A_2—C9_2—H9B_2	107.2	C7_12—C8_12—C9_12	115.2 (4)
C11_2—C10_2—C9_2	120.2 (7)	C7_12—C8_12—H8A_12	108.5
C11_2—C10_2—H10A_2	107.3	C9_12—C8_12—H8A_12	108.5
C9_2—C10_2—H10A_2	107.3	C7_12—C8_12—H8B_12	108.5
C11_2—C10_2—H10B_2	107.3	C9_12—C8_12—H8B_12	108.5
C9_2—C10_2—H10B_2	107.3	H8A_12—C8_12—H8B_12	107.5
H10A_2—C10_2—H10B_2	106.9	C10_12—C9_12—C8_12	112.9 (4)
C10_2—C11_2—H11A_2	109.5	C10_12—C9_12—H9A_12	109.0
C10_2—C11_2—H11B_2	109.5	C8_12—C9_12—H9A_12	109.0
H11A_2—C11_2—H11B_2	109.5	C10_12—C9_12—H9B_12	109.0
C10_2—C11_2—H11C_2	109.5	C8_12—C9_12—H9B_12	109.0
H11A_2—C11_2—H11C_2	109.5	H9A_12—C9_12—H9B_12	107.8
H11B_2—C11_2—H11C_2	109.5	C11_12—C10_12—C9_12	115.4 (5)
C1_3—N1_3—N2_3	107.3 (4)	C11_12—C10_12—H10A_12	108.4
C1_3—N1_3—Ni3_16	138.7 (3)	C9_12—C10_12—H10A_12	108.4
N2_3—N1_3—Ni3_16	113.3 (3)	C11_12—C10_12—H10B_12	108.4
C3_3—N2_3—N1_3	108.0 (4)	C9_12—C10_12—H10B_12	108.4
C3_3—N2_3—Ni2_16	136.7 (3)	H10A_12—C10_12—H10B_12	107.5
N1_3—N2_3—Ni2_16	114.8 (3)	C10_12—C11_12—H11A_12	109.5
N1_3—C1_3—C2_3	110.6 (4)	C10_12—C11_12—H11B_12	109.5
N1_3—C1_3—H1_3	124.7	H11A_12—C11_12—H11B_12	109.5
C2_3—C1_3—H1_3	124.7	C10_12—C11_12—H11C_12	109.5
C1_3—C2_3—C3_3	103.4 (4)	H11A_12—C11_12—H11C_12	109.5
C1_3—C2_3—C4B_3	127.4 (5)	H11B_12—C11_12—H11C_12	109.5
C3_3—C2_3—C4B_3	129.1 (5)	C5_13—N1_13—C9_13	112.2 (4)
C1_3—C2_3—C4_3	127.4 (5)	C5_13—N1_13—C1_13	108.4 (4)
C3_3—C2_3—C4_3	129.1 (5)	C9_13—N1_13—C1_13	107.4 (4)
N2_3—C3_3—C2_3	110.6 (5)	C5_13—N1_13—C13_13	109.1 (4)
N2_3—C3_3—H3_3	124.7	C9_13—N1_13—C13_13	108.1 (4)
C2_3—C3_3—H3_3	124.7	C1_13—N1_13—C13_13	111.6 (4)
C5_3—C4_3—C2_3	115.5 (6)	C2_13—C1_13—N1_13	116.2 (5)
C5_3—C4_3—H4A_3	108.4	C2_13—C1_13—H1A_13	108.2
C2_3—C4_3—H4A_3	108.4	N1_13—C1_13—H1A_13	108.2
C5_3—C4_3—H4B_3	108.4	C2_13—C1_13—H1B_13	108.2
C2_3—C4_3—H4B_3	108.4	N1_13—C1_13—H1B_13	108.2
H4A_3—C4_3—H4B_3	107.5	H1A_13—C1_13—H1B_13	107.4
C4_3—C5_3—C6_3	115.3 (8)	C1_13—C2_13—C3_13	113.0 (5)
C4_3—C5_3—H5A_3	108.5	C1_13—C2_13—H2A_13	109.0
C6_3—C5_3—H5A_3	108.5	C3_13—C2_13—H2A_13	109.0
C4_3—C5_3—H5B_3	108.5	C1_13—C2_13—H2B_13	109.0
C6_3—C5_3—H5B_3	108.5	C3_13—C2_13—H2B_13	109.0
H5A_3—C5_3—H5B_3	107.5	H2A_13—C2_13—H2B_13	107.8
C7_3—C6_3—C5_3	119.6 (10)	C4_13—C3_13—C2_13	116.5 (7)
C7_3—C6_3—H6A_3	107.4	C4_13—C3_13—H3A_13	108.2
C5_3—C6_3—H6A_3	107.4	C2_13—C3_13—H3A_13	108.2
C7_3—C6_3—H6B_3	107.4	C4_13—C3_13—H3B_13	108.2
C5_3—C6_3—H6B_3	107.4	C2_13—C3_13—H3B_13	108.2
H6A_3—C6_3—H6B_3	107.0	H3A_13—C3_13—H3B_13	107.3
C6_3—C7_3—C8_3	120.0 (10)	C3_13—C4_13—H4A_13	109.5
C6_3—C7_3—H7A_3	107.3	C3_13—C4_13—H4B_13	109.5
C8_3—C7_3—H7A_3	107.3	H4A_13—C4_13—H4B_13	109.5
C6_3—C7_3—H7B_3	107.3	C3_13—C4_13—H4C_13	109.5
C8_3—C7_3—H7B_3	107.3	H4A_13—C4_13—H4C_13	109.5
H7A_3—C7_3—H7B_3	106.9	H4B_13—C4_13—H4C_13	109.5
C7_3—C8_3—C9_3	114.3 (11)	N1_13—C5_13—C6_13	115.1 (5)
C7_3—C8_3—H8A_3	108.7	N1_13—C5_13—H5A_13	108.5
C9_3—C8_3—H8A_3	108.7	C6_13—C5_13—H5A_13	108.5
C7_3—C8_3—H8B_3	108.7	N1_13—C5_13—H5B_13	108.5
C9_3—C8_3—H8B_3	108.7	C6_13—C5_13—H5B_13	108.5
H8A_3—C8_3—H8B_3	107.6	H5A_13—C5_13—H5B_13	107.5
C10_3—C9_3—C8_3	109.9 (11)	C7_13—C6_13—C5_13	111.3 (6)
C10_3—C9_3—H9A_3	109.7	C7_13—C6_13—H6A_13	109.4
C8_3—C9_3—H9A_3	109.7	C5_13—C6_13—H6A_13	109.4
C10_3—C9_3—H9B_3	109.7	C7_13—C6_13—H6B_13	109.4
C8_3—C9_3—H9B_3	109.7	C5_13—C6_13—H6B_13	109.4
H9A_3—C9_3—H9B_3	108.2	H6A_13—C6_13—H6B_13	108.0
C11_3—C10_3—C9_3	113.1 (13)	C6_13—C7_13—C8_13	112.8 (6)
C11_3—C10_3—H10A_3	109.0	C6_13—C7_13—H7A_13	109.0
C9_3—C10_3—H10A_3	109.0	C8_13—C7_13—H7A_13	109.0
C11_3—C10_3—H10B_3	109.0	C6_13—C7_13—H7B_13	109.0
C9_3—C10_3—H10B_3	109.0	C8_13—C7_13—H7B_13	109.0
H10A_3—C10_3—H10B_3	107.8	H7A_13—C7_13—H7B_13	107.8
C10_3—C11_3—H11A_3	109.5	C7_13—C8_13—H8A_13	109.5
C10_3—C11_3—H11B_3	109.5	C7_13—C8_13—H8B_13	109.5
H11A_3—C11_3—H11B_3	109.5	H8A_13—C8_13—H8B_13	109.5
C10_3—C11_3—H11C_3	109.5	C7_13—C8_13—H8C_13	109.5
H11A_3—C11_3—H11C_3	109.5	H8A_13—C8_13—H8C_13	109.5
H11B_3—C11_3—H11C_3	109.5	H8B_13—C8_13—H8C_13	109.5
C5B_3—C4B_3—C2_3	117.9 (11)	C10_13—C9_13—N1_13	116.9 (4)
C5B_3—C4B_3—H4BA_3	107.8	C10_13—C9_13—H9A_13	108.1
C2_3—C4B_3—H4BA_3	107.8	N1_13—C9_13—H9A_13	108.1
C5B_3—C4B_3—H4BB_3	107.8	C10_13—C9_13—H9B_13	108.1
C2_3—C4B_3—H4BB_3	107.8	N1_13—C9_13—H9B_13	108.1
H4BA_3—C4B_3—H4BB_3	107.2	H9A_13—C9_13—H9B_13	107.3
C4B_3—C5B_3—C6B_3	126.0 (18)	C11_13—C10_13—C9_13	109.4 (5)
C4B_3—C5B_3—H5BA_3	105.8	C11_13—C10_13—H10A_13	109.8
C6B_3—C5B_3—H5BA_3	105.8	C9_13—C10_13—H10A_13	109.8
C4B_3—C5B_3—H5BB_3	105.8	C11_13—C10_13—H10B_13	109.8
C6B_3—C5B_3—H5BB_3	105.8	C9_13—C10_13—H10B_13	109.8
H5BA_3—C5B_3—H5BB_3	106.2	H10A_13—C10_13—H10B_13	108.2
C7B_3—C6B_3—C5B_3	128 (2)	C12_13—C11_13—C10_13	117.9 (6)
C7B_3—C6B_3—H6BA_3	105.2	C12_13—C11_13—H11A_13	107.8
C5B_3—C6B_3—H6BA_3	105.2	C10_13—C11_13—H11A_13	107.8
C7B_3—C6B_3—H6BB_3	105.2	C12_13—C11_13—H11B_13	107.8
C5B_3—C6B_3—H6BB_3	105.2	C10_13—C11_13—H11B_13	107.8
H6BA_3—C6B_3—H6BB_3	105.9	H11A_13—C11_13—H11B_13	107.2
C6B_3—C7B_3—C8B_3	117 (2)	C11_13—C12_13—H12A_13	109.5
C6B_3—C7B_3—H7BA_3	108.0	C11_13—C12_13—H12B_13	109.5
C8B_3—C7B_3—H7BA_3	108.0	H12A_13—C12_13—H12B_13	109.5
C6B_3—C7B_3—H7BB_3	108.0	C11_13—C12_13—H12C_13	109.5
C8B_3—C7B_3—H7BB_3	108.0	H12A_13—C12_13—H12C_13	109.5
H7BA_3—C7B_3—H7BB_3	107.2	H12B_13—C12_13—H12C_13	109.5
C7B_3—C8B_3—C9B_3	111.4 (19)	C14_13—C13_13—N1_13	117.3 (5)
C7B_3—C8B_3—H8BA_3	109.3	C14_13—C13_13—H13A_13	108.0
C9B_3—C8B_3—H8BA_3	109.3	N1_13—C13_13—H13A_13	108.0
C7B_3—C8B_3—H8BB_3	109.3	C14_13—C13_13—H13B_13	108.0
C9B_3—C8B_3—H8BB_3	109.3	N1_13—C13_13—H13B_13	108.0
H8BA_3—C8B_3—H8BB_3	108.0	H13A_13—C13_13—H13B_13	107.2
C10B_3—C9B_3—C8B_3	117 (2)	C15_13—C14_13—C13_13	114.0 (5)
C10B_3—C9B_3—H9BA_3	108.0	C15_13—C14_13—H14A_13	108.7
C8B_3—C9B_3—H9BA_3	108.0	C13_13—C14_13—H14A_13	108.7
C10B_3—C9B_3—H9BB_3	108.0	C15_13—C14_13—H14B_13	108.7
C8B_3—C9B_3—H9BB_3	108.0	C13_13—C14_13—H14B_13	108.7
H9BA_3—C9B_3—H9BB_3	107.2	H14A_13—C14_13—H14B_13	107.6
C11B_3—C10B_3—C9B_3	113 (2)	C14_13—C15_13—C16_13	115.2 (5)
C11B_3—C10B_3—H10C_3	109.1	C14_13—C15_13—H15A_13	108.5
C9B_3—C10B_3—H10C_3	109.1	C16_13—C15_13—H15A_13	108.5
C11B_3—C10B_3—H10D_3	109.1	C14_13—C15_13—H15B_13	108.5
C9B_3—C10B_3—H10D_3	109.1	C16_13—C15_13—H15B_13	108.5
H10C_3—C10B_3—H10D_3	107.8	H15A_13—C15_13—H15B_13	107.5
C10B_3—C11B_3—H11D_3	109.5	C15_13—C16_13—H16A_13	109.5
C10B_3—C11B_3—H11E_3	109.5	C15_13—C16_13—H16B_13	109.5
H11D_3—C11B_3—H11E_3	109.5	H16A_13—C16_13—H16B_13	109.5
C10B_3—C11B_3—H11F_3	109.5	C15_13—C16_13—H16C_13	109.5
H11D_3—C11B_3—H11F_3	109.5	H16A_13—C16_13—H16C_13	109.5
H11E_3—C11B_3—H11F_3	109.5	H16B_13—C16_13—H16C_13	109.5
C1_4—N1_4—N2_4	107.4 (4)	C5_14—N1_14—C13_14	109.5 (8)
C1_4—N1_4—Ni3_16	136.4 (4)	C5_14—N1_14—C9_14	108.6 (10)
N2_4—N1_4—Ni3_16	113.2 (3)	C13_14—N1_14—C9_14	110.5 (9)
C3_4—N2_4—N1_4	107.7 (4)	C5_14—N1_14—C1_14	111.7 (11)
C3_4—N2_4—Ni4_16	138.2 (4)	C13_14—N1_14—C1_14	105.7 (8)
N1_4—N2_4—Ni4_16	113.5 (3)	C9_14—N1_14—C1_14	110.8 (11)
N1_4—C1_4—C2_4	111.0 (5)	C2_14—C1_14—N1_14	113.4 (10)
N1_4—C1_4—H1_4	124.5	C2_14—C1_14—H1A_14	108.9
C2_4—C1_4—H1_4	124.5	N1_14—C1_14—H1A_14	108.9
C1_4—C2_4—C3_4	103.2 (4)	C2_14—C1_14—H1B_14	108.9
C1_4—C2_4—C4B_4	129.3 (6)	N1_14—C1_14—H1B_14	108.9
C3_4—C2_4—C4B_4	127.4 (6)	H1A_14—C1_14—H1B_14	107.7
C1_4—C2_4—C4_4	129.3 (6)	C3_14—C2_14—C1_14	114.0 (10)
C3_4—C2_4—C4_4	127.4 (6)	C3_14—C2_14—H2A_14	108.7
N2_4—C3_4—C2_4	110.8 (5)	C1_14—C2_14—H2A_14	108.7
N2_4—C3_4—H3_4	124.6	C3_14—C2_14—H2B_14	108.7
C2_4—C3_4—H3_4	124.6	C1_14—C2_14—H2B_14	108.7
C2_4—C4_4—C5_4	111.5 (13)	H2A_14—C2_14—H2B_14	107.6
C2_4—C4_4—H4A_4	109.3	C4_14—C3_14—C2_14	113.1 (10)
C5_4—C4_4—H4A_4	109.3	C4_14—C3_14—H3A_14	109.0
C2_4—C4_4—H4B_4	109.3	C2_14—C3_14—H3A_14	109.0
C5_4—C4_4—H4B_4	109.3	C4_14—C3_14—H3B_14	109.0
H4A_4—C4_4—H4B_4	108.0	C2_14—C3_14—H3B_14	109.0
C6_4—C5_4—C4_4	121.3 (14)	H3A_14—C3_14—H3B_14	107.8
C6_4—C5_4—H5A_4	107.0	C3_14—C4_14—H4A_14	109.5
C4_4—C5_4—H5A_4	107.0	C3_14—C4_14—H4B_14	109.5
C6_4—C5_4—H5B_4	107.0	H4A_14—C4_14—H4B_14	109.5
C4_4—C5_4—H5B_4	107.0	C3_14—C4_14—H4C_14	109.5
H5A_4—C5_4—H5B_4	106.7	H4A_14—C4_14—H4C_14	109.5
C5_4—C6_4—C7_4	114.1 (15)	H4B_14—C4_14—H4C_14	109.5
C5_4—C6_4—H6A_4	108.7	C6_14—C5_14—N1_14	119.1 (9)
C7_4—C6_4—H6A_4	108.7	C6_14—C5_14—H5A_14	107.6
C5_4—C6_4—H6B_4	108.7	N1_14—C5_14—H5A_14	107.6
C7_4—C6_4—H6B_4	108.7	C6_14—C5_14—H5B_14	107.6
H6A_4—C6_4—H6B_4	107.6	N1_14—C5_14—H5B_14	107.6
C8_4—C7_4—C6_4	116.2 (12)	H5A_14—C5_14—H5B_14	107.0
C8_4—C7_4—H7A_4	108.2	C5_14—C6_14—C7_14	117.8 (10)
C6_4—C7_4—H7A_4	108.2	C5_14—C6_14—H6A_14	107.9
C8_4—C7_4—H7B_4	108.2	C7_14—C6_14—H6A_14	107.9
C6_4—C7_4—H7B_4	108.2	C5_14—C6_14—H6B_14	107.9
H7A_4—C7_4—H7B_4	107.4	C7_14—C6_14—H6B_14	107.9
C9_4—C8_4—C7_4	113.0 (12)	H6A_14—C6_14—H6B_14	107.2
C9_4—C8_4—H8A_4	109.0	C6_14—C7_14—C8_14	113.7 (11)
C7_4—C8_4—H8A_4	109.0	C6_14—C7_14—H7A_14	108.8
C9_4—C8_4—H8B_4	109.0	C8_14—C7_14—H7A_14	108.8
C7_4—C8_4—H8B_4	109.0	C6_14—C7_14—H7B_14	108.8
H8A_4—C8_4—H8B_4	107.8	C8_14—C7_14—H7B_14	108.8
C8_4—C9_4—C10_4	112.4 (13)	H7A_14—C7_14—H7B_14	107.7
C8_4—C9_4—H9A_4	109.1	C7_14—C8_14—H8A_14	109.5
C10_4—C9_4—H9A_4	109.1	C7_14—C8_14—H8B_14	109.5
C8_4—C9_4—H9B_4	109.1	H8A_14—C8_14—H8B_14	109.5
C10_4—C9_4—H9B_4	109.1	C7_14—C8_14—H8C_14	109.5
H9A_4—C9_4—H9B_4	107.9	H8A_14—C8_14—H8C_14	109.5
C11_4—C10_4—C9_4	113.2 (16)	H8B_14—C8_14—H8C_14	109.5
C11_4—C10_4—H10A_4	108.9	C10_14—C9_14—N1_14	118.3 (9)
C9_4—C10_4—H10A_4	108.9	C10_14—C9_14—H9A_14	107.7
C11_4—C10_4—H10B_4	108.9	N1_14—C9_14—H9A_14	107.7
C9_4—C10_4—H10B_4	108.9	C10_14—C9_14—H9B_14	107.7
H10A_4—C10_4—H10B_4	107.8	N1_14—C9_14—H9B_14	107.7
C10_4—C11_4—H11A_4	109.5	H9A_14—C9_14—H9B_14	107.1
C10_4—C11_4—H11B_4	109.5	C9_14—C10_14—C11_14	119.2 (10)
H11A_4—C11_4—H11B_4	109.5	C9_14—C10_14—H10A_14	107.5
C10_4—C11_4—H11C_4	109.5	C11_14—C10_14—H10A_14	107.5
H11A_4—C11_4—H11C_4	109.5	C9_14—C10_14—H10B_14	107.5
H11B_4—C11_4—H11C_4	109.5	C11_14—C10_14—H10B_14	107.5
C2_4—C4B_4—C5B_4	116.4 (15)	H10A_14—C10_14—H10B_14	107.0
C2_4—C4B_4—H4BA_4	108.2	C10_14—C11_14—C12_14	112.6 (12)
C5B_4—C4B_4—H4BA_4	108.2	C10_14—C11_14—H11A_14	109.1
C2_4—C4B_4—H4BB_4	108.2	C12_14—C11_14—H11A_14	109.1
C5B_4—C4B_4—H4BB_4	108.2	C10_14—C11_14—H11B_14	109.1
H4BA_4—C4B_4—H4BB_4	107.3	C12_14—C11_14—H11B_14	109.1
C4B_4—C5B_4—C6B_4	106.4 (12)	H11A_14—C11_14—H11B_14	107.8
C4B_4—C5B_4—H5BA_4	110.5	C11_14—C12_14—H12A_14	109.5
C6B_4—C5B_4—H5BA_4	110.5	C11_14—C12_14—H12B_14	109.5
C4B_4—C5B_4—H5BB_4	110.5	H12A_14—C12_14—H12B_14	109.5
C6B_4—C5B_4—H5BB_4	110.5	C11_14—C12_14—H12C_14	109.5
H5BA_4—C5B_4—H5BB_4	108.6	H12A_14—C12_14—H12C_14	109.5
C7B_4—C6B_4—C5B_4	113.0 (12)	H12B_14—C12_14—H12C_14	109.5
C7B_4—C6B_4—H6BA_4	109.0	N1_14—C13_14—C14_14	115.5 (11)
C5B_4—C6B_4—H6BA_4	109.0	N1_14—C13_14—H13A_14	108.4
C7B_4—C6B_4—H6BB_4	109.0	C14_14—C13_14—H13A_14	108.4
C5B_4—C6B_4—H6BB_4	109.0	N1_14—C13_14—H13B_14	108.4
H6BA_4—C6B_4—H6BB_4	107.8	C14_14—C13_14—H13B_14	108.4
C6B_4—C7B_4—C8B_4	111.9 (12)	H13A_14—C13_14—H13B_14	107.5
C6B_4—C7B_4—H7BA_4	109.2	C15_14—C14_14—C13_14	110.7 (12)
C8B_4—C7B_4—H7BA_4	109.2	C15_14—C14_14—H14A_14	109.5
C6B_4—C7B_4—H7BB_4	109.2	C13_14—C14_14—H14A_14	109.5
C8B_4—C7B_4—H7BB_4	109.2	C15_14—C14_14—H14B_14	109.5
H7BA_4—C7B_4—H7BB_4	107.9	C13_14—C14_14—H14B_14	109.5
C7B_4—C8B_4—C9B_4	110.7 (12)	H14A_14—C14_14—H14B_14	108.1
C7B_4—C8B_4—H8BA_4	109.5	C14_14—C15_14—C16_14	112.0 (11)
C9B_4—C8B_4—H8BA_4	109.5	C14_14—C15_14—H15A_14	109.2
C7B_4—C8B_4—H8BB_4	109.5	C16_14—C15_14—H15A_14	109.2
C9B_4—C8B_4—H8BB_4	109.5	C14_14—C15_14—H15B_14	109.2
H8BA_4—C8B_4—H8BB_4	108.1	C16_14—C15_14—H15B_14	109.2
C10B_4—C9B_4—C8B_4	111.9 (14)	H15A_14—C15_14—H15B_14	107.9
C10B_4—C9B_4—H9BA_4	109.2	C15_14—C16_14—H16A_14	109.5
C8B_4—C9B_4—H9BA_4	109.2	C15_14—C16_14—H16B_14	109.5
C10B_4—C9B_4—H9BB_4	109.2	H16A_14—C16_14—H16B_14	109.5
C8B_4—C9B_4—H9BB_4	109.2	C15_14—C16_14—H16C_14	109.5
H9BA_4—C9B_4—H9BB_4	107.9	H16A_14—C16_14—H16C_14	109.5
C11B_4—C10B_4—C9B_4	112.2 (16)	H16B_14—C16_14—H16C_14	109.5
C11B_4—C10B_4—H10C_4	109.2	C5_15—N1_15—C13_15	109.5 (18)
C9B_4—C10B_4—H10C_4	109.2	C5_15—N1_15—C1_15	114.2 (18)
C11B_4—C10B_4—H10D_4	109.2	C13_15—N1_15—C1_15	103.8 (16)
C9B_4—C10B_4—H10D_4	109.2	C5_15—N1_15—C9_15	108.7 (17)
H10C_4—C10B_4—H10D_4	107.9	C13_15—N1_15—C9_15	111.2 (16)
C10B_4—C11B_4—H11D_4	109.5	C1_15—N1_15—C9_15	109.4 (18)
C10B_4—C11B_4—H11E_4	109.5	C2_15—C1_15—N1_15	113.9 (17)
H11D_4—C11B_4—H11E_4	109.5	C2_15—C1_15—H1A_15	108.8
C10B_4—C11B_4—H11F_4	109.5	N1_15—C1_15—H1A_15	108.8
H11D_4—C11B_4—H11F_4	109.5	C2_15—C1_15—H1B_15	108.8
H11E_4—C11B_4—H11F_4	109.5	N1_15—C1_15—H1B_15	108.8
C1_5—N1_5—N2_5	107.3 (4)	H1A_15—C1_15—H1B_15	107.7
C1_5—N1_5—Ni4_16	137.8 (4)	C3_15—C2_15—C1_15	113 (2)
N2_5—N1_5—Ni4_16	113.9 (3)	C3_15—C2_15—H2A_15	108.9
C3_5—N2_5—N1_5	108.2 (5)	C1_15—C2_15—H2A_15	108.9
C3_5—N2_5—Ni2_16^i^	138.5 (4)	C3_15—C2_15—H2B_15	108.9
N1_5—N2_5—Ni2_16^i^	113.3 (3)	C1_15—C2_15—H2B_15	108.9
N1_5—C1_5—C2_5	111.2 (5)	H2A_15—C2_15—H2B_15	107.7
N1_5—C1_5—H1_5	124.4	C2_15—C3_15—C4_15	117 (2)
C2_5—C1_5—H1_5	124.4	C2_15—C3_15—H3A_15	108.0
C1_5—C2_5—C3_5	103.4 (5)	C4_15—C3_15—H3A_15	108.0
C1_5—C2_5—C4B_5	129.1 (6)	C2_15—C3_15—H3B_15	108.0
C3_5—C2_5—C4B_5	127.4 (6)	C4_15—C3_15—H3B_15	108.0
C1_5—C2_5—C4_5	129.1 (6)	H3A_15—C3_15—H3B_15	107.3
C3_5—C2_5—C4_5	127.4 (6)	C3_15—C4_15—H4A_15	109.5
N2_5—C3_5—C2_5	110.0 (5)	C3_15—C4_15—H4B_15	109.5
N2_5—C3_5—H3_5	125.0	H4A_15—C4_15—H4B_15	109.5
C2_5—C3_5—H3_5	125.0	C3_15—C4_15—H4C_15	109.5
C5_5—C4_5—C2_5	114.3 (9)	H4A_15—C4_15—H4C_15	109.5
C5_5—C4_5—H4A_5	108.7	H4B_15—C4_15—H4C_15	109.5
C2_5—C4_5—H4A_5	108.7	N1_15—C5_15—C6_15	115.8 (18)
C5_5—C4_5—H4B_5	108.7	N1_15—C5_15—H5A_15	108.3
C2_5—C4_5—H4B_5	108.7	C6_15—C5_15—H5A_15	108.3
H4A_5—C4_5—H4B_5	107.6	N1_15—C5_15—H5B_15	108.3
C4_5—C5_5—C6_5	115.1 (13)	C6_15—C5_15—H5B_15	108.3
C4_5—C5_5—H5A_5	108.5	H5A_15—C5_15—H5B_15	107.4
C6_5—C5_5—H5A_5	108.5	C7_15—C6_15—C5_15	112 (2)
C4_5—C5_5—H5B_5	108.5	C7_15—C6_15—H6A_15	109.1
C6_5—C5_5—H5B_5	108.5	C5_15—C6_15—H6A_15	109.1
H5A_5—C5_5—H5B_5	107.5	C7_15—C6_15—H6B_15	109.1
C7_5—C6_5—C5_5	108.7 (14)	C5_15—C6_15—H6B_15	109.1
C7_5—C6_5—H6A_5	109.9	H6A_15—C6_15—H6B_15	107.9
C5_5—C6_5—H6A_5	109.9	C8_15—C7_15—C6_15	115 (2)
C7_5—C6_5—H6B_5	109.9	C8_15—C7_15—H7A_15	108.4
C5_5—C6_5—H6B_5	109.9	C6_15—C7_15—H7A_15	108.4
H6A_5—C6_5—H6B_5	108.3	C8_15—C7_15—H7B_15	108.4
C8_5—C7_5—C6_5	117.0 (17)	C6_15—C7_15—H7B_15	108.4
C8_5—C7_5—H7A_5	108.1	H7A_15—C7_15—H7B_15	107.5
C6_5—C7_5—H7A_5	108.1	C7_15—C8_15—H8A_15	109.5
C8_5—C7_5—H7B_5	108.1	C7_15—C8_15—H8B_15	109.5
C6_5—C7_5—H7B_5	108.1	H8A_15—C8_15—H8B_15	109.5
H7A_5—C7_5—H7B_5	107.3	C7_15—C8_15—H8C_15	109.5
C9_5—C8_5—C7_5	125.7 (19)	H8A_15—C8_15—H8C_15	109.5
C9_5—C8_5—H8A_5	105.9	H8B_15—C8_15—H8C_15	109.5
C7_5—C8_5—H8A_5	105.9	C10_15—C9_15—N1_15	123 (2)
C9_5—C8_5—H8B_5	105.9	C10_15—C9_15—H9A_15	106.7
C7_5—C8_5—H8B_5	105.9	N1_15—C9_15—H9A_15	106.7
H8A_5—C8_5—H8B_5	106.2	C10_15—C9_15—H9B_15	106.7
C8_5—C9_5—C10_5	113.3 (18)	N1_15—C9_15—H9B_15	106.7
C8_5—C9_5—H9A_5	108.9	H9A_15—C9_15—H9B_15	106.6
C10_5—C9_5—H9A_5	108.9	C9_15—C10_15—C11_15	120 (2)
C8_5—C9_5—H9B_5	108.9	C9_15—C10_15—H10A_15	107.3
C10_5—C9_5—H9B_5	108.9	C11_15—C10_15—H10A_15	107.3
H9A_5—C9_5—H9B_5	107.7	C9_15—C10_15—H10B_15	107.3
C11_5—C10_5—C9_5	103.1 (17)	C11_15—C10_15—H10B_15	107.3
C11_5—C10_5—H10A_5	111.2	H10A_15—C10_15—H10B_15	106.9
C9_5—C10_5—H10A_5	111.2	C10_15—C11_15—C12_15	117 (3)
C11_5—C10_5—H10B_5	111.2	C10_15—C11_15—H11A_15	108.1
C9_5—C10_5—H10B_5	111.2	C12_15—C11_15—H11A_15	108.1
H10A_5—C10_5—H10B_5	109.1	C10_15—C11_15—H11B_15	108.1
C10_5—C11_5—H11A_5	109.5	C12_15—C11_15—H11B_15	108.1
C10_5—C11_5—H11B_5	109.5	H11A_15—C11_15—H11B_15	107.3
H11A_5—C11_5—H11B_5	109.5	C11_15—C12_15—H12A_15	109.5
C10_5—C11_5—H11C_5	109.5	C11_15—C12_15—H12B_15	109.5
H11A_5—C11_5—H11C_5	109.5	H12A_15—C12_15—H12B_15	109.5
H11B_5—C11_5—H11C_5	109.5	C11_15—C12_15—H12C_15	109.5
C2_5—C4B_5—C5B_5	112.3 (10)	H12A_15—C12_15—H12C_15	109.5
C2_5—C4B_5—H4BA_5	109.2	H12B_15—C12_15—H12C_15	109.5
C5B_5—C4B_5—H4BA_5	109.2	N1_15—C13_15—C14_15	114.3 (19)
C2_5—C4B_5—H4BB_5	109.2	N1_15—C13_15—H13A_15	108.7
C5B_5—C4B_5—H4BB_5	109.2	C14_15—C13_15—H13A_15	108.7
H4BA_5—C4B_5—H4BB_5	107.9	N1_15—C13_15—H13B_15	108.7
C6B_5—C5B_5—C4B_5	115.9 (13)	C14_15—C13_15—H13B_15	108.7
C6B_5—C5B_5—H5BA_5	108.3	H13A_15—C13_15—H13B_15	107.6
C4B_5—C5B_5—H5BA_5	108.3	C15_15—C14_15—C13_15	112 (2)
C6B_5—C5B_5—H5BB_5	108.3	C15_15—C14_15—H14A_15	109.3
C4B_5—C5B_5—H5BB_5	108.3	C13_15—C14_15—H14A_15	109.3
H5BA_5—C5B_5—H5BB_5	107.4	C15_15—C14_15—H14B_15	109.3
C7B_5—C6B_5—C5B_5	107.5 (15)	C13_15—C14_15—H14B_15	109.3
C7B_5—C6B_5—H6BA_5	110.2	H14A_15—C14_15—H14B_15	108.0
C5B_5—C6B_5—H6BA_5	110.2	C14_15—C15_15—C16_15	120 (3)
C7B_5—C6B_5—H6BB_5	110.2	C14_15—C15_15—H15A_15	107.3
C5B_5—C6B_5—H6BB_5	110.2	C16_15—C15_15—H15A_15	107.3
H6BA_5—C6B_5—H6BB_5	108.5	C14_15—C15_15—H15B_15	107.3
C8B_5—C7B_5—C6B_5	103.7 (16)	C16_15—C15_15—H15B_15	107.3
C8B_5—C7B_5—H7BA_5	111.0	H15A_15—C15_15—H15B_15	106.9
C6B_5—C7B_5—H7BA_5	111.0	C15_15—C16_15—H16A_15	109.5
C8B_5—C7B_5—H7BB_5	111.0	C15_15—C16_15—H16B_15	109.5
C6B_5—C7B_5—H7BB_5	111.0	H16A_15—C16_15—H16B_15	109.5
H7BA_5—C7B_5—H7BB_5	109.0	C15_15—C16_15—H16C_15	109.5
C9B_5—C8B_5—C7B_5	109.7 (16)	H16A_15—C16_15—H16C_15	109.5
C9B_5—C8B_5—H8BA_5	109.7	H16B_15—C16_15—H16C_15	109.5
C7B_5—C8B_5—H8BA_5	109.7	C5B_15—N1B_15—C1B_15	113.3 (19)
C9B_5—C8B_5—H8BB_5	109.7	C5B_15—N1B_15—C13B_15	113.2 (19)
C7B_5—C8B_5—H8BB_5	109.7	C1B_15—N1B_15—C13B_15	106.6 (18)
H8BA_5—C8B_5—H8BB_5	108.2	C5B_15—N1B_15—C9B_15	107.0 (17)
C8B_5—C9B_5—C10B_5	101.6 (16)	C1B_15—N1B_15—C9B_15	110.7 (16)
C8B_5—C9B_5—H9BA_5	111.5	C13B_15—N1B_15—C9B_15	105.9 (17)
C10B_5—C9B_5—H9BA_5	111.5	C2B_15—C1B_15—N1B_15	121 (2)
C8B_5—C9B_5—H9BB_5	111.5	C2B_15—C1B_15—H1C_15	107.0
C10B_5—C9B_5—H9BB_5	111.5	N1B_15—C1B_15—H1C_15	107.0
H9BA_5—C9B_5—H9BB_5	109.3	C2B_15—C1B_15—H1D_15	107.0
C11B_5—C10B_5—C9B_5	100.2 (19)	N1B_15—C1B_15—H1D_15	107.0
C11B_5—C10B_5—H10C_5	111.7	H1C_15—C1B_15—H1D_15	106.7
C9B_5—C10B_5—H10C_5	111.7	C3B_15—C2B_15—C1B_15	112 (2)
C11B_5—C10B_5—H10D_5	111.7	C3B_15—C2B_15—H2C_15	109.3
C9B_5—C10B_5—H10D_5	111.7	C1B_15—C2B_15—H2C_15	109.3
H10C_5—C10B_5—H10D_5	109.5	C3B_15—C2B_15—H2D_15	109.3
C10B_5—C11B_5—H11D_5	109.5	C1B_15—C2B_15—H2D_15	109.3
C10B_5—C11B_5—H11E_5	109.5	H2C_15—C2B_15—H2D_15	107.9
H11D_5—C11B_5—H11E_5	109.5	C2B_15—C3B_15—C4B_15	120 (3)
C10B_5—C11B_5—H11F_5	109.5	C2B_15—C3B_15—H3C_15	107.2
H11D_5—C11B_5—H11F_5	109.5	C4B_15—C3B_15—H3C_15	107.2
H11E_5—C11B_5—H11F_5	109.5	C2B_15—C3B_15—H3D_15	107.2
C1_6—N1_6—N2_6	107.6 (4)	C4B_15—C3B_15—H3D_15	107.2
C1_6—N1_6—Ni4_16	138.9 (4)	H3C_15—C3B_15—H3D_15	106.9
N2_6—N1_6—Ni4_16	113.3 (3)	C3B_15—C4B_15—H4D_15	109.5
C3_6—N2_6—N1_6	108.0 (4)	C3B_15—C4B_15—H4E_15	109.5
C3_6—N2_6—Ni1_16	137.8 (4)	H4D_15—C4B_15—H4E_15	109.5
N1_6—N2_6—Ni1_16	113.6 (3)	C3B_15—C4B_15—H4F_15	109.5
N1_6—C1_6—C2_6	110.5 (5)	H4D_15—C4B_15—H4F_15	109.5
N1_6—C1_6—H1_6	124.8	H4E_15—C4B_15—H4F_15	109.5
C2_6—C1_6—H1_6	124.8	C6B_15—C5B_15—N1B_15	117.4 (19)
C1_6—C2_6—C3_6	103.8 (5)	C6B_15—C5B_15—H5C_15	108.0
C1_6—C2_6—C4_6	127.6 (6)	N1B_15—C5B_15—H5C_15	108.0
C3_6—C2_6—C4_6	128.6 (6)	C6B_15—C5B_15—H5D_15	108.0
N2_6—C3_6—C2_6	110.1 (5)	N1B_15—C5B_15—H5D_15	108.0
N2_6—C3_6—H3_6	125.0	H5C_15—C5B_15—H5D_15	107.2
C2_6—C3_6—H3_6	125.0	C7B_15—C6B_15—C5B_15	113 (2)
C2_6—C4_6—C5_6	112.5 (6)	C7B_15—C6B_15—H6C_15	109.0
C2_6—C4_6—H4A_6	109.1	C5B_15—C6B_15—H6C_15	109.0
C5_6—C4_6—H4A_6	109.1	C7B_15—C6B_15—H6D_15	109.0
C2_6—C4_6—H4B_6	109.1	C5B_15—C6B_15—H6D_15	109.0
C5_6—C4_6—H4B_6	109.1	H6C_15—C6B_15—H6D_15	107.8
H4A_6—C4_6—H4B_6	107.8	C8B_15—C7B_15—C6B_15	114 (2)
C4_6—C5_6—C6_6	116.1 (7)	C8B_15—C7B_15—H7C_15	108.6
C4_6—C5_6—H5A_6	108.3	C6B_15—C7B_15—H7C_15	108.6
C6_6—C5_6—H5A_6	108.3	C8B_15—C7B_15—H7D_15	108.6
C4_6—C5_6—H5B_6	108.3	C6B_15—C7B_15—H7D_15	108.6
C6_6—C5_6—H5B_6	108.3	H7C_15—C7B_15—H7D_15	107.6
H5A_6—C5_6—H5B_6	107.4	C7B_15—C8B_15—H8D_15	109.5
C7_6—C6_6—C5_6	116.4 (8)	C7B_15—C8B_15—H8E_15	109.5
C7_6—C6_6—H6A_6	108.2	H8D_15—C8B_15—H8E_15	109.5
C5_6—C6_6—H6A_6	108.2	C7B_15—C8B_15—H8F_15	109.5
C7_6—C6_6—H6B_6	108.2	H8D_15—C8B_15—H8F_15	109.5
C5_6—C6_6—H6B_6	108.2	H8E_15—C8B_15—H8F_15	109.5
H6A_6—C6_6—H6B_6	107.3	C10B_15—C9B_15—N1B_15	118.5 (19)
C6_6—C7_6—C8_6	115.5 (9)	C10B_15—C9B_15—H9C_15	107.7
C6_6—C7_6—H7A_6	108.4	N1B_15—C9B_15—H9C_15	107.7
C8_6—C7_6—H7A_6	108.4	C10B_15—C9B_15—H9D_15	107.7
C6_6—C7_6—H7B_6	108.4	N1B_15—C9B_15—H9D_15	107.7
C8_6—C7_6—H7B_6	108.4	H9C_15—C9B_15—H9D_15	107.1
H7A_6—C7_6—H7B_6	107.5	C9B_15—C10B_15—C11B_15	123 (2)
C7_6—C8_6—C9_6	113.5 (10)	C9B_15—C10B_15—H10C_15	106.7
C7_6—C8_6—H8A_6	108.9	C11B_15—C10B_15—H10C_15	106.7
C9_6—C8_6—H8A_6	108.9	C9B_15—C10B_15—H10D_15	106.7
C7_6—C8_6—H8B_6	108.9	C11B_15—C10B_15—H10D_15	106.7
C9_6—C8_6—H8B_6	108.9	H10C_15—C10B_15—H10D_15	106.6
H8A_6—C8_6—H8B_6	107.7	C10B_15—C11B_15—C12B_15	113 (3)
C10_6—C9_6—C8_6	117.3 (13)	C10B_15—C11B_15—H11C_15	109.0
C10_6—C9_6—H9A_6	108.0	C12B_15—C11B_15—H11C_15	109.0
C8_6—C9_6—H9A_6	108.0	C10B_15—C11B_15—H11D_15	109.0
C10_6—C9_6—H9B_6	108.0	C12B_15—C11B_15—H11D_15	109.0
C8_6—C9_6—H9B_6	108.0	H11C_15—C11B_15—H11D_15	107.8
H9A_6—C9_6—H9B_6	107.2	C11B_15—C12B_15—H12D_15	109.5
C9_6—C10_6—C11_6	114.8 (15)	C11B_15—C12B_15—H12E_15	109.5
C9_6—C10_6—H10A_6	108.6	H12D_15—C12B_15—H12E_15	109.5
C11_6—C10_6—H10A_6	108.6	C11B_15—C12B_15—H12F_15	109.5
C9_6—C10_6—H10B_6	108.6	H12D_15—C12B_15—H12F_15	109.5
C11_6—C10_6—H10B_6	108.6	H12E_15—C12B_15—H12F_15	109.5
H10A_6—C10_6—H10B_6	107.5	N1B_15—C13B_15—C14B_15	121.0 (18)
C10_6—C11_6—H11A_6	109.5	N1B_15—C13B_15—H13C_15	107.1
C10_6—C11_6—H11B_6	109.5	C14B_15—C13B_15—H13C_15	107.1
H11A_6—C11_6—H11B_6	109.5	N1B_15—C13B_15—H13D_15	107.1
C10_6—C11_6—H11C_6	109.5	C14B_15—C13B_15—H13D_15	107.1
H11A_6—C11_6—H11C_6	109.5	H13C_15—C13B_15—H13D_15	106.8
H11B_6—C11_6—H11C_6	109.5	C15B_15—C14B_15—C13B_15	105.3 (19)
C1_7—N1_7—N2_7	107.3 (4)	C15B_15—C14B_15—H14C_15	110.7
C1_7—N1_7—Ni8_16	139.4 (3)	C13B_15—C14B_15—H14C_15	110.7
N2_7—N1_7—Ni8_16	113.3 (3)	C15B_15—C14B_15—H14D_15	110.7
C3_7—N2_7—N1_7	107.6 (4)	C13B_15—C14B_15—H14D_15	110.7
C3_7—N2_7—Ni5_16	138.4 (3)	H14C_15—C14B_15—H14D_15	108.8
N1_7—N2_7—Ni5_16	114.0 (3)	C14B_15—C15B_15—C16B_15	119 (2)
N1_7—C1_7—C2_7	110.8 (4)	C14B_15—C15B_15—H15C_15	107.6
N1_7—C1_7—H1_7	124.6	C16B_15—C15B_15—H15C_15	107.6
C2_7—C1_7—H1_7	124.6	C14B_15—C15B_15—H15D_15	107.6
C3_7—C2_7—C1_7	103.5 (4)	C16B_15—C15B_15—H15D_15	107.6
C3_7—C2_7—C4_7	127.2 (5)	H15C_15—C15B_15—H15D_15	107.0
C1_7—C2_7—C4_7	129.2 (5)	C15B_15—C16B_15—H16D_15	109.5
N2_7—C3_7—C2_7	110.9 (4)	C15B_15—C16B_15—H16E_15	109.5
N2_7—C3_7—H3_7	124.6	H16D_15—C16B_15—H16E_15	109.5
C2_7—C3_7—H3_7	124.6	C15B_15—C16B_15—H16F_15	109.5
C2_7—C4_7—C5B_7	114.2 (5)	H16D_15—C16B_15—H16F_15	109.5
C2_7—C4_7—C5_7	114.2 (5)	H16E_15—C16B_15—H16F_15	109.5
C2_7—C4_7—H4A_7	108.7		
			
C1_1—N1_1—N2_1—C3_1	−0.4 (7)	N1_8—C1_8—C2_8—C3_8	−0.1 (5)
Ni1_16—N1_1—N2_1—C3_1	170.0 (4)	N1_8—C1_8—C2_8—C4_8	−178.8 (5)
C1_1—N1_1—N2_1—Ni3_16^i^	−168.8 (4)	N1_8—N2_8—C3_8—C2_8	0.2 (6)
Ni1_16—N1_1—N2_1—Ni3_16^i^	1.7 (5)	Ni6_16—N2_8—C3_8—C2_8	175.7 (4)
N2_1—N1_1—C1_1—C2_1	0.7 (7)	C1_8—C2_8—C3_8—N2_8	−0.1 (6)
Ni1_16—N1_1—C1_1—C2_1	−167.0 (5)	C4_8—C2_8—C3_8—N2_8	178.8 (5)
N1_1—C1_1—C2_1—C3_1	−0.6 (8)	C3_8—C2_8—C4_8—C5_8	−166.9 (5)
N1_1—C1_1—C2_1—C4_1	176.5 (7)	C1_8—C2_8—C4_8—C5_8	11.6 (8)
N1_1—N2_1—C3_1—C2_1	0.0 (7)	C2_8—C4_8—C5_8—C6_8	−75.8 (6)
Ni3_16^i^—N2_1—C3_1—C2_1	163.4 (5)	C4_8—C5_8—C6_8—C7_8	−171.9 (5)
C1_1—C2_1—C3_1—N2_1	0.3 (7)	C5_8—C6_8—C7_8—C8_8	176.3 (6)
C4_1—C2_1—C3_1—N2_1	−176.7 (7)	C6_8—C7_8—C8_8—C9_8	179.2 (6)
C3_1—C2_1—C4_1—C5_1	169.1 (7)	C7_8—C8_8—C9_8—C10_8	−179.4 (7)
C1_1—C2_1—C4_1—C5_1	−7.3 (11)	C8_8—C9_8—C10_8—C11_8	177.7 (8)
C2_1—C4_1—C5_1—C6_1	−177.7 (7)	C1_9—N1_9—N2_9—C3_9	0.2 (5)
C4_1—C5_1—C6_1—C7_1	−174.6 (8)	Ni8_16—N1_9—N2_9—C3_9	−171.0 (3)
C5_1—C6_1—C7_1—C8_1	−179.2 (8)	C1_9—N1_9—N2_9—Ni7_16	175.8 (3)
C6_1—C7_1—C8_1—C9_1	−175.8 (10)	Ni8_16—N1_9—N2_9—Ni7_16	4.7 (3)
C7_1—C8_1—C9_1—C10_1	177.0 (11)	N2_9—N1_9—C1_9—C2_9	−0.1 (5)
C8_1—C9_1—C10_1—C11_1	−178.1 (13)	Ni8_16—N1_9—C1_9—C2_9	168.0 (3)
C1_2—N1_2—N2_2—C3_2	−0.1 (6)	N1_9—C1_9—C2_9—C3_9	0.0 (5)
Ni2_16—N1_2—N2_2—C3_2	179.9 (4)	N1_9—C1_9—C2_9—C4_9	−176.8 (5)
C1_2—N1_2—N2_2—Ni1_16	−177.5 (4)	N1_9—N2_9—C3_9—C2_9	−0.2 (5)
Ni2_16—N1_2—N2_2—Ni1_16	2.5 (4)	Ni7_16—N2_9—C3_9—C2_9	−174.1 (3)
N2_2—N1_2—C1_2—C2_2	0.1 (7)	C1_9—C2_9—C3_9—N2_9	0.1 (5)
Ni2_16—N1_2—C1_2—C2_2	−180.0 (5)	C4_9—C2_9—C3_9—N2_9	176.9 (5)
N1_2—C1_2—C2_2—C3_2	0.0 (8)	C3_9—C2_9—C4_9—C5_9	−93.0 (7)
N1_2—C1_2—C2_2—C4_2	−177.3 (7)	C1_9—C2_9—C4_9—C5_9	83.0 (7)
N1_2—N2_2—C3_2—C2_2	0.1 (7)	C2_9—C4_9—C5_9—C6_9	−63.3 (6)
Ni1_16—N2_2—C3_2—C2_2	176.5 (5)	C4_9—C5_9—C6_9—C7_9	−70.7 (7)
C1_2—C2_2—C3_2—N2_2	0.0 (8)	C5_9—C6_9—C7_9—C8_9	178.5 (5)
C4_2—C2_2—C3_2—N2_2	177.1 (8)	C6_9—C7_9—C8_9—C9_9	175.0 (6)
C3_2—C2_2—C4_2—C5_2	29.1 (15)	C7_9—C8_9—C9_9—C10_9	179.8 (7)
C1_2—C2_2—C4_2—C5_2	−154.3 (8)	C8_9—C9_9—C10_9—C11_9	−178.2 (8)
C2_2—C4_2—C5_2—C6_2	−178.1 (8)	C1_10—N1_10—N2_10—C3_10	−0.7 (5)
C4_2—C5_2—C6_2—C7_2	−174.9 (9)	Ni6_16—N1_10—N2_10—C3_10	−170.4 (3)
C5_2—C6_2—C7_2—C8_2	−179.5 (8)	C1_10—N1_10—N2_10—Ni7_16	165.7 (3)
C6_2—C7_2—C8_2—C9_2	−176.5 (8)	Ni6_16—N1_10—N2_10—Ni7_16	−4.0 (4)
C7_2—C8_2—C9_2—C10_2	−179.0 (8)	N2_10—N1_10—C1_10—C2_10	0.4 (5)
C8_2—C9_2—C10_2—C11_2	−176.0 (9)	Ni6_16—N1_10—C1_10—C2_10	166.3 (4)
C1_3—N1_3—N2_3—C3_3	−0.8 (6)	N1_10—C1_10—C2_10—C3_10	0.1 (6)
Ni3_16—N1_3—N2_3—C3_3	−173.0 (4)	N1_10—C1_10—C2_10—C4B_10	−173.3 (5)
C1_3—N1_3—N2_3—Ni2_16	172.5 (3)	N1_10—C1_10—C2_10—C4_10	−173.3 (5)
Ni3_16—N1_3—N2_3—Ni2_16	0.3 (4)	N1_10—N2_10—C3_10—C2_10	0.8 (5)
N2_3—N1_3—C1_3—C2_3	1.1 (6)	Ni7_16—N2_10—C3_10—C2_10	−161.2 (4)
Ni3_16—N1_3—C1_3—C2_3	170.3 (4)	C1_10—C2_10—C3_10—N2_10	−0.5 (6)
N1_3—C1_3—C2_3—C3_3	−1.0 (7)	C4B_10—C2_10—C3_10—N2_10	172.9 (5)
N1_3—C1_3—C2_3—C4B_3	179.6 (6)	C4_10—C2_10—C3_10—N2_10	172.9 (5)
N1_3—C1_3—C2_3—C4_3	179.6 (6)	C1_10—C2_10—C4_10—C5_10	94.2 (12)
N1_3—N2_3—C3_3—C2_3	0.2 (7)	C3_10—C2_10—C4_10—C5_10	−77.7 (13)
Ni2_16—N2_3—C3_3—C2_3	−170.9 (4)	C2_10—C4_10—C5_10—C6_10	176.6 (13)
C1_3—C2_3—C3_3—N2_3	0.5 (7)	C4_10—C5_10—C6_10—C7_10	172.2 (18)
C4B_3—C2_3—C3_3—N2_3	179.9 (6)	C5_10—C6_10—C7_10—C8_10	−177.1 (19)
C4_3—C2_3—C3_3—N2_3	179.9 (6)	C6_10—C7_10—C8_10—C9_10	178 (2)
C1_3—C2_3—C4_3—C5_3	166.7 (7)	C7_10—C8_10—C9_10—C10_10	−115 (3)
C3_3—C2_3—C4_3—C5_3	−12.6 (11)	C8_10—C9_10—C10_10—C11_10	−136 (3)
C2_3—C4_3—C5_3—C6_3	−64.9 (11)	C1_10—C2_10—C4B_10—C5B_10	77.6 (13)
C4_3—C5_3—C6_3—C7_3	−178.9 (9)	C3_10—C2_10—C4B_10—C5B_10	−94.2 (13)
C5_3—C6_3—C7_3—C8_3	179.5 (11)	C2_10—C4B_10—C5B_10—C6B_10	−179.6 (15)
C6_3—C7_3—C8_3—C9_3	174.8 (11)	C4B_10—C5B_10—C6B_10—C7B_10	177 (2)
C7_3—C8_3—C9_3—C10_3	178.8 (11)	C5B_10—C6B_10—C7B_10—C8B_10	−175 (2)
C8_3—C9_3—C10_3—C11_3	−79.8 (17)	C6B_10—C7B_10—C8B_10—C9B_10	−175 (2)
C1_3—C2_3—C4B_3—C5B_3	134.3 (14)	C7B_10—C8B_10—C9B_10—C10B_10	151 (3)
C3_3—C2_3—C4B_3—C5B_3	−44.9 (16)	C8B_10—C9B_10—C10B_10—C11B_10	162 (3)
C2_3—C4B_3—C5B_3—C6B_3	46 (3)	C1_11—N1_11—N2_11—C3_11	−1.0 (5)
C4B_3—C5B_3—C6B_3—C7B_3	148 (3)	Ni6_16—N1_11—N2_11—C3_11	171.6 (3)
C5B_3—C6B_3—C7B_3—C8B_3	138 (3)	C1_11—N1_11—N2_11—Ni8_16^ii^	−177.6 (3)
C6B_3—C7B_3—C8B_3—C9B_3	−100 (3)	Ni6_16—N1_11—N2_11—Ni8_16^ii^	−5.1 (4)
C7B_3—C8B_3—C9B_3—C10B_3	160 (3)	N2_11—N1_11—C1_11—C2_11	−0.1 (6)
C8B_3—C9B_3—C10B_3—C11B_3	167 (4)	Ni6_16—N1_11—C1_11—C2_11	−169.7 (4)
C1_4—N1_4—N2_4—C3_4	0.2 (5)	N1_11—C1_11—C2_11—C3_11	1.0 (6)
Ni3_16—N1_4—N2_4—C3_4	−163.5 (3)	N1_11—C1_11—C2_11—C4_11	175.2 (5)
C1_4—N1_4—N2_4—Ni4_16	172.7 (3)	N1_11—N2_11—C3_11—C2_11	1.7 (6)
Ni3_16—N1_4—N2_4—Ni4_16	8.9 (4)	Ni8_16^ii^—N2_11—C3_11—C2_11	177.1 (4)
N2_4—N1_4—C1_4—C2_4	−0.1 (6)	C1_11—C2_11—C3_11—N2_11	−1.6 (6)
Ni3_16—N1_4—C1_4—C2_4	157.9 (4)	C4_11—C2_11—C3_11—N2_11	−175.8 (5)
N1_4—C1_4—C2_4—C3_4	−0.1 (6)	C3_11—C2_11—C4_11—C5_11	86.0 (7)
N1_4—C1_4—C2_4—C4B_4	−177.4 (5)	C1_11—C2_11—C4_11—C5_11	−86.8 (7)
N1_4—C1_4—C2_4—C4_4	−177.4 (5)	C2_11—C4_11—C5_11—C6_11	174.0 (5)
N1_4—N2_4—C3_4—C2_4	−0.3 (6)	C4_11—C5_11—C6_11—C7_11	−178.9 (5)
Ni4_16—N2_4—C3_4—C2_4	−169.8 (4)	C5_11—C6_11—C7_11—C8_11	177.6 (5)
C1_4—C2_4—C3_4—N2_4	0.2 (6)	C6_11—C7_11—C8_11—C9_11	179.8 (5)
C4B_4—C2_4—C3_4—N2_4	177.6 (5)	C7_11—C8_11—C9_11—C10_11	−178.8 (6)
C4_4—C2_4—C3_4—N2_4	177.6 (5)	C8_11—C9_11—C10_11—C11_11	178.7 (6)
C1_4—C2_4—C4_4—C5_4	73.2 (11)	C1_12—N1_12—N2_12—C3_12	0.2 (5)
C3_4—C2_4—C4_4—C5_4	−103.5 (11)	Ni7_16—N1_12—N2_12—C3_12	−170.8 (3)
C2_4—C4_4—C5_4—C6_4	−64 (3)	C1_12—N1_12—N2_12—Ni5_16^ii^	174.4 (3)
C4_4—C5_4—C6_4—C7_4	−62 (3)	Ni7_16—N1_12—N2_12—Ni5_16^ii^	3.3 (4)
C5_4—C6_4—C7_4—C8_4	−176.8 (14)	N2_12—N1_12—C1_12—C2_12	0.2 (6)
C6_4—C7_4—C8_4—C9_4	67.4 (18)	Ni7_16—N1_12—C1_12—C2_12	167.5 (4)
C7_4—C8_4—C9_4—C10_4	169.6 (17)	N1_12—C1_12—C2_12—C3_12	−0.5 (6)
C8_4—C9_4—C10_4—C11_4	−178.4 (19)	N1_12—C1_12—C2_12—C4_12	−175.7 (5)
C1_4—C2_4—C4B_4—C5B_4	73.1 (12)	N1_12—N2_12—C3_12—C2_12	−0.6 (5)
C3_4—C2_4—C4B_4—C5B_4	−103.6 (11)	Ni5_16^ii^—N2_12—C3_12—C2_12	−172.7 (3)
C2_4—C4B_4—C5B_4—C6B_4	−79 (2)	C1_12—C2_12—C3_12—N2_12	0.7 (6)
C4B_4—C5B_4—C6B_4—C7B_4	−174.0 (17)	C4_12—C2_12—C3_12—N2_12	176.0 (4)
C5B_4—C6B_4—C7B_4—C8B_4	178.3 (19)	C1_12—C2_12—C4_12—C5_12	100.0 (6)
C6B_4—C7B_4—C8B_4—C9B_4	172.4 (15)	C3_12—C2_12—C4_12—C5_12	−74.1 (6)
C7B_4—C8B_4—C9B_4—C10B_4	−172.2 (18)	C2_12—C4_12—C5_12—C6_12	175.9 (4)
C8B_4—C9B_4—C10B_4—C11B_4	177 (2)	C4_12—C5_12—C6_12—C7_12	−179.8 (5)
C1_5—N1_5—N2_5—C3_5	0.4 (6)	C5_12—C6_12—C7_12—C8_12	177.3 (5)
Ni4_16—N1_5—N2_5—C3_5	−170.5 (4)	C6_12—C7_12—C8_12—C9_12	178.9 (5)
C1_5—N1_5—N2_5—Ni2_16^i^	179.0 (4)	C7_12—C8_12—C9_12—C10_12	177.7 (6)
Ni4_16—N1_5—N2_5—Ni2_16^i^	8.2 (4)	C8_12—C9_12—C10_12—C11_12	179.0 (6)
N2_5—N1_5—C1_5—C2_5	−0.2 (7)	C5_13—N1_13—C1_13—C2_13	69.3 (6)
Ni4_16—N1_5—C1_5—C2_5	167.4 (5)	C9_13—N1_13—C1_13—C2_13	−169.2 (5)
N1_5—C1_5—C2_5—C3_5	−0.1 (8)	C13_13—N1_13—C1_13—C2_13	−50.9 (6)
N1_5—C1_5—C2_5—C4B_5	−175.5 (7)	N1_13—C1_13—C2_13—C3_13	−179.0 (6)
N1_5—C1_5—C2_5—C4_5	−175.5 (7)	C1_13—C2_13—C3_13—C4_13	−171.7 (7)
N1_5—N2_5—C3_5—C2_5	−0.4 (7)	C9_13—N1_13—C5_13—C6_13	47.0 (7)
Ni2_16^i^—N2_5—C3_5—C2_5	−178.6 (5)	C1_13—N1_13—C5_13—C6_13	165.4 (5)
C1_5—C2_5—C3_5—N2_5	0.3 (8)	C13_13—N1_13—C5_13—C6_13	−72.8 (6)
C4B_5—C2_5—C3_5—N2_5	175.9 (7)	N1_13—C5_13—C6_13—C7_13	169.8 (5)
C4_5—C2_5—C3_5—N2_5	175.9 (7)	C5_13—C6_13—C7_13—C8_13	173.3 (6)
C1_5—C2_5—C4_5—C5_5	68.8 (14)	C5_13—N1_13—C9_13—C10_13	60.8 (7)
C3_5—C2_5—C4_5—C5_5	−105.7 (11)	C1_13—N1_13—C9_13—C10_13	−58.3 (6)
C2_5—C4_5—C5_5—C6_5	−68.8 (19)	C13_13—N1_13—C9_13—C10_13	−178.9 (5)
C4_5—C5_5—C6_5—C7_5	−177.9 (15)	N1_13—C9_13—C10_13—C11_13	−163.2 (6)
C5_5—C6_5—C7_5—C8_5	−174.6 (18)	C9_13—C10_13—C11_13—C12_13	−48.4 (10)
C6_5—C7_5—C8_5—C9_5	−77 (3)	C5_13—N1_13—C13_13—C14_13	−170.5 (5)
C7_5—C8_5—C9_5—C10_5	−149 (2)	C9_13—N1_13—C13_13—C14_13	67.2 (6)
C8_5—C9_5—C10_5—C11_5	−121 (2)	C1_13—N1_13—C13_13—C14_13	−50.7 (6)
C1_5—C2_5—C4B_5—C5B_5	99.0 (12)	N1_13—C13_13—C14_13—C15_13	−126.9 (5)
C3_5—C2_5—C4B_5—C5B_5	−75.4 (11)	C13_13—C14_13—C15_13—C16_13	76.9 (7)
C2_5—C4B_5—C5B_5—C6B_5	−63.3 (17)	C5_14—N1_14—C1_14—C2_14	67.1 (17)
C4B_5—C5B_5—C6B_5—C7B_5	−57 (2)	C13_14—N1_14—C1_14—C2_14	−173.9 (12)
C5B_5—C6B_5—C7B_5—C8B_5	−168.5 (17)	C9_14—N1_14—C1_14—C2_14	−54.1 (17)
C6B_5—C7B_5—C8B_5—C9B_5	−180.0 (18)	N1_14—C1_14—C2_14—C3_14	−165.9 (12)
C7B_5—C8B_5—C9B_5—C10B_5	−169 (2)	C1_14—C2_14—C3_14—C4_14	−176.0 (13)
C8B_5—C9B_5—C10B_5—C11B_5	−144 (2)	C13_14—N1_14—C5_14—C6_14	−70.0 (12)
C1_6—N1_6—N2_6—C3_6	0.6 (6)	C9_14—N1_14—C5_14—C6_14	169.3 (10)
Ni4_16—N1_6—N2_6—C3_6	−175.6 (3)	C1_14—N1_14—C5_14—C6_14	46.8 (15)
C1_6—N1_6—N2_6—Ni1_16	−172.2 (4)	N1_14—C5_14—C6_14—C7_14	76.3 (14)
Ni4_16—N1_6—N2_6—Ni1_16	11.5 (4)	C5_14—C6_14—C7_14—C8_14	155.4 (11)
N2_6—N1_6—C1_6—C2_6	0.0 (6)	C5_14—N1_14—C9_14—C10_14	−176.6 (10)
Ni4_16—N1_6—C1_6—C2_6	174.7 (4)	C13_14—N1_14—C9_14—C10_14	63.3 (12)
N1_6—C1_6—C2_6—C3_6	−0.6 (7)	C1_14—N1_14—C9_14—C10_14	−53.6 (15)
N1_6—C1_6—C2_6—C4_6	−179.3 (6)	N1_14—C9_14—C10_14—C11_14	−79.3 (14)
N1_6—N2_6—C3_6—C2_6	−1.0 (6)	C9_14—C10_14—C11_14—C12_14	−156.1 (11)
Ni1_16—N2_6—C3_6—C2_6	169.2 (4)	C5_14—N1_14—C13_14—C14_14	−57.8 (14)
C1_6—C2_6—C3_6—N2_6	0.9 (7)	C9_14—N1_14—C13_14—C14_14	61.7 (13)
C4_6—C2_6—C3_6—N2_6	179.7 (6)	C1_14—N1_14—C13_14—C14_14	−178.3 (18)
C1_6—C2_6—C4_6—C5_6	−101.4 (9)	N1_14—C13_14—C14_14—C15_14	−172.3 (11)
C3_6—C2_6—C4_6—C5_6	80.2 (9)	C13_14—C14_14—C15_14—C16_14	−175.9 (13)
C2_6—C4_6—C5_6—C6_6	−68.8 (9)	C5_15—N1_15—C1_15—C2_15	56 (3)
C4_6—C5_6—C6_6—C7_6	−74.6 (9)	C13_15—N1_15—C1_15—C2_15	175 (3)
C5_6—C6_6—C7_6—C8_6	179.2 (7)	C9_15—N1_15—C1_15—C2_15	−66 (3)
C6_6—C7_6—C8_6—C9_6	171.0 (9)	N1_15—C1_15—C2_15—C3_15	166 (3)
C7_6—C8_6—C9_6—C10_6	−179.0 (12)	C1_15—C2_15—C3_15—C4_15	−176 (3)
C8_6—C9_6—C10_6—C11_6	173.1 (12)	C13_15—N1_15—C5_15—C6_15	−72 (3)
C1_7—N1_7—N2_7—C3_7	0.2 (5)	C1_15—N1_15—C5_15—C6_15	43 (4)
Ni8_16—N1_7—N2_7—C3_7	−179.5 (3)	C9_15—N1_15—C5_15—C6_15	166 (3)
C1_7—N1_7—N2_7—Ni5_16	178.6 (3)	N1_15—C5_15—C6_15—C7_15	178 (3)
Ni8_16—N1_7—N2_7—Ni5_16	−1.1 (4)	C5_15—C6_15—C7_15—C8_15	−159 (4)
N2_7—N1_7—C1_7—C2_7	0.4 (5)	C5_15—N1_15—C9_15—C10_15	151 (3)
Ni8_16—N1_7—C1_7—C2_7	180.0 (4)	C13_15—N1_15—C9_15—C10_15	30 (3)
N1_7—C1_7—C2_7—C3_7	−0.8 (6)	C1_15—N1_15—C9_15—C10_15	−84 (3)
N1_7—C1_7—C2_7—C4_7	−177.7 (5)	N1_15—C9_15—C10_15—C11_15	68 (4)
N1_7—N2_7—C3_7—C2_7	−0.7 (5)	C9_15—C10_15—C11_15—C12_15	170 (4)
Ni5_16—N2_7—C3_7—C2_7	−178.6 (4)	C5_15—N1_15—C13_15—C14_15	−40 (3)
C1_7—C2_7—C3_7—N2_7	0.9 (6)	C1_15—N1_15—C13_15—C14_15	−162 (2)
C4_7—C2_7—C3_7—N2_7	177.9 (5)	C9_15—N1_15—C13_15—C14_15	80 (3)
C3_7—C2_7—C4_7—C5B_7	−87.4 (7)	N1_15—C13_15—C14_15—C15_15	−90 (3)
C1_7—C2_7—C4_7—C5B_7	88.7 (7)	C13_15—C14_15—C15_15—C16_15	−161 (4)
C3_7—C2_7—C4_7—C5_7	−87.4 (7)	C5B_15—N1B_15—C1B_15—C2B_15	93 (3)
C1_7—C2_7—C4_7—C5_7	88.7 (7)	C13B_15—N1B_15—C1B_15—C2B_15	−142 (3)
C2_7—C4_7—C5_7—C6_7	−179 (3)	C9B_15—N1B_15—C1B_15—C2B_15	−27 (3)
C4_7—C5_7—C6_7—C7_7	170 (3)	N1B_15—C1B_15—C2B_15—C3B_15	−76 (4)
C5_7—C6_7—C7_7—C8_7	−180 (3)	C1B_15—C2B_15—C3B_15—C4B_15	−155 (4)
C6_7—C7_7—C8_7—C9_7	−178 (3)	C1B_15—N1B_15—C5B_15—C6B_15	64 (4)
C7_7—C8_7—C9_7—C10_7	178 (2)	C13B_15—N1B_15—C5B_15—C6B_15	−58 (4)
C8_7—C9_7—C10_7—C11_7	175 (3)	C9B_15—N1B_15—C5B_15—C6B_15	−174 (3)
C2_7—C4_7—C5B_7—C6B_7	−179 (2)	N1B_15—C5B_15—C6B_15—C7B_15	−170 (4)
C4_7—C5B_7—C6B_7—C7B_7	−173 (2)	C5B_15—C6B_15—C7B_15—C8B_15	−171 (4)
C5B_7—C6B_7—C7B_7—C8B_7	−177 (2)	C5B_15—N1B_15—C9B_15—C10B_15	−160 (3)
C6B_7—C7B_7—C8B_7—C9B_7	177 (3)	C1B_15—N1B_15—C9B_15—C10B_15	−36 (3)
C7B_7—C8B_7—C9B_7—C10B_7	−176.5 (18)	C13B_15—N1B_15—C9B_15—C10B_15	79 (3)
C8B_7—C9B_7—C10B_7—C11B_7	64 (3)	N1B_15—C9B_15—C10B_15—C11B_15	−57 (5)
C1_8—N1_8—N2_8—C3_8	−0.2 (5)	C9B_15—C10B_15—C11B_15—C12B_15	−164 (4)
Ni5_16—N1_8—N2_8—C3_8	174.3 (3)	C5B_15—N1B_15—C13B_15—C14B_15	−93 (3)
C1_8—N1_8—N2_8—Ni6_16	−176.9 (3)	C1B_15—N1B_15—C13B_15—C14B_15	142 (3)
Ni5_16—N1_8—N2_8—Ni6_16	−2.3 (4)	C9B_15—N1B_15—C13B_15—C14B_15	24 (3)
N2_8—N1_8—C1_8—C2_8	0.2 (5)	N1B_15—C13B_15—C14B_15—C15B_15	152 (3)
Ni5_16—N1_8—C1_8—C2_8	−172.2 (4)	C13B_15—C14B_15—C15B_15—C16B_15	−161 (3)

Symmetry codes: (i) −*x*+1, −*y*+1, −*z*+1; (ii) −*x*+1, −*y*+1, −*z*.
